# Comparing the Effectiveness of Multimodal Learning Using Computer-Based and Immersive Virtual Reality Simulation–Based Interprofessional Education With Co-Debriefing, Medical Movies, and Massive Online Open Courses for Mitigating Stress and Long-Term Burnout in Medical Training: Quasi-Experimental Study

**DOI:** 10.2196/70726

**Published:** 2025-09-24

**Authors:** Sirikanyawan Srikasem, Sunisa Seephom, Atthaphon Viriyopase, Phanupong Phutrakool, Sirhavich Khowinthaseth, Khuansiri Narajeenron, ER-VIPE Study Group

**Affiliations:** 1 Adult and Gerontological Nursing Department Srisavarindhira Thai Red Cross Institute of Nursing Bangkok Thailand; 2 Department of Emergency Medicine Faculty of Medicine Chulalongkorn University Bangkok Thailand; 3 Chula Data Management Center Faculty of Medicine Chulalongkorn University Bangkok Thailand; 4 Department of Emergency Medicine King Chulalongkorn Memorial Hospital The Thai Red Cross Society Bangkok Thailand; 5 See Acknowledgments

**Keywords:** emergency medicine, stress, anxiety, burnout, interprofessional education, virtual reality, simulation, medical movies, MOOCs, simulation-based interprofessional education, emergency room virtual interprofessional education

## Abstract

**Background:**

Burnout among emergency room health care workers (HCWs) has reached critical levels, affecting up to 43% of HCWs and 35% of emergency medicine personnel during the COVID-19 pandemic. Nurses were most affected, followed by physicians, leading to absenteeism, reduced care quality, and turnover rates as high as 78% in some settings such as Thailand. Beyond workforce instability, burnout compromises patient safety. Each 1-unit increase in emotional exhaustion has been linked to a 2.63-fold rise in reports of poor care quality, 30% increase in patient falls, 47% increase in medication errors, and 32% increase in health care–associated infections. Burnout is also associated with lower job satisfaction, worsening mental health, and increased intent to leave the profession. These findings underscore the urgent need for effective strategies to reduce stress and burnout in emergency care.

**Objective:**

This study aimed to evaluate the effectiveness and effect size of a multimodal learning approach—Emergency Room Virtual Simulation Interprofessional Education (ER-VIPE)—that integrates medical movies, massive online open courses (MOOCs), and computer- or virtual reality (VR)–based simulations with co-debriefing for reducing burnout and stress among future health care professionals compared with approaches lacking co-debriefing or using only movies and MOOCs.

**Methods:**

A single-blind, quasi-experimental study was conducted at a university hospital from August 2022 to September 2023 using a 3-group treatment design. Group A (control) participated in a 3D computer-based, simulation-based interprofessional education (SIMBIE) without debriefing. Group B received the ER-VIPE intervention. Group C received the same as Group B, but the computer-based SIMBIE was replaced with 3D VR-SIMBIE. SIMBIE activities simulated a COVID-19 pneumonia crisis. Outcomes included the Dundee Stress State Questionnaire (DSSQ) and the Copenhagen Burnout Inventory, with trait anxiety as a behavioral control. Stress and burnout were measured at baseline, pre-intervention, postintervention, and 1-month follow-up. Generalized estimating equations were used to analyze group differences, with statistical significance set at *P*<.05.

**Results:**

We randomized 87 undergraduate students from various health programs into the 3 groups (n=29 each). Participants’ mean age was 22 years, with 71% (62/87) as women. After the 1-month post-SIMBIE follow-up, adjusted analyses revealed positive trends in DSSQ-engagement across all groups, with Group B showing a significant increase compared with Group A (mean difference=3.93; *P*=.001). DSSQ-worry and DSSQ-distress scores decreased nonsignificantly across all groups. Burnout scores also improved across groups, with Group B having a significantly lower score than Group A (mean difference=–2.02; *P*=.02). No significant burnout differences were found between Group C and Groups A or B.

**Conclusions:**

A multimodal learning approach combining medical movies, MOOCs, and 3D computer-based SIMBIE with co-debriefing effectively improved engagement, reduced stress, and lowered burnout among future health care professionals. This scalable educational framework may help enhance well-being and resilience in high-pressure clinical environments.

## Introduction

### Background

Prior to the onset of the COVID-19 pandemic, burnout was already a significant concern among emergency health care professionals. For example, 60% of emergency medicine (EM) physicians [1] and 26% of emergency nurses [2] reported experiencing burnout on a regular basis. Although no quantitative, emergency department–specific studies from Thailand exist for the pre-COVID-19 period, broader national surveys have documented substantial levels of burnout among Thai nurses. In a study of more than 2000 nurses working in community hospitals, 32% reported high emotional exhaustion, 18% reported high depersonalization, and 35% experienced low personal accomplishment [3]. Notably, Nantsupawat et al [3] found that each 1-unit increase in emotional exhaustion score was associated with a 2.63-fold increase in the likelihood of reporting fair or poor quality of care, 30% increase in patient falls, 47% increase in medication errors, and 32% increase in infections. Supplementing this quantitative evidence, a qualitative study by Yuwanich et al [4] identified unique emergency department–specific stressors, such as patients’ and their relatives’ behaviors as well as power imbalances, affecting nurses in Thailand. Together, these baseline findings underscore that emergency health care workers (HCWs) were already highly vulnerable to burnout prior to the COVID-19 pandemic.

Working in an emergency room during crises or pandemics under tight time constraints, limited experience, and with newly formed teams is highly stressful. HCWs face heavy workloads, interpersonal conflicts, limited social support, and high-acuity patients [5]. The need for rapid, complex decision-making in this unpredictable environment intensifies stress, contributing to high burnout rates, especially during COVID-19 [6-8]. A systematic review reported that the prevalence of overall burnout among HCWs during the COVID-19 pandemic was high, reaching 43%, with 35% of EM HCWs identified as being at high risk for burnout [9]. The profession type—such as nurse, physician, or resident—was a significant factor influencing the rate of burnout and its domains, whereas gender did not show a consistent association. Among EM professionals, nurses were the most affected, followed by physicians [9]. The psychological impact of the COVID-19 pandemic extended beyond burnout, encompassing a wide range of adverse mental health outcomes, including stress, anxiety, depression, inadequate sleep, posttraumatic stress disorder symptoms, and secondary trauma [10]. Anxiety, depression, stress, and burnout levels were notably higher among physicians and nurses than in other health care roles, consistent with findings from other systematic reviews [9,11]. Gualano et al [11] further identified several pandemic-related factors associated with increased burnout risk, such as resource shortages, fear of COVID-19 infection, and social stigma. Consequently, the prevalence of turnover intention among emergency nurses has been reported at 45% globally [12], with a notably higher rate of 78% observed in Thailand [13]. Thailand is currently facing a critical nursing workforce shortage, reflected in a nurse-to-population ratio of approximately 1:400. Projections estimate an annual attrition of around 7000 nurses [13].

The COVID-19 health care crisis has profoundly impacted frontline health care professionals, exposing them to cumulative traumatic experiences that elevate stress and burnout levels. Frontline workers consistently report higher emotional exhaustion and depersonalization compared with those in non-COVID-19 units. Resilience and adaptive defense mechanisms are crucial for mitigating these effects, while younger age, female gender, increased COVID-19 exposure, and less resilient coping strategies predict greater vulnerability to stress and burnout. These findings highlight the urgent need for support programs focused on resilience-building and stress management for HCWs directly involved in COVID-19 patient care [14].

Health care workforce retention is a growing global concern, particularly in the postpandemic context. Depression and anxiety also positively correlate with higher absenteeism rates [15], reduced job commitment [16], lower job satisfaction [17], more medical leave [18], and poorer quality of life [19]. A 2023 study in Thailand reported that the revised model fit the data and accounted for 45% of the variance in nurses’ intention to leave [20]. Burnout was the strongest factor, affecting intention both directly and indirectly via job satisfaction and professional commitment. Work-family conflict and the nursing practice environment influenced intention indirectly through these pathways. Thailand also faces a physician shortage, with only 0.5 to 0.8 doctors per 1000 people—below the World Health Organization (WHO) standard of 1:1000. The pandemic exacerbated these challenges, leaving many frontline providers with burnout and limited mental health support [21]. These findings highlight the urgent need for targeted retention strategies. Thailand’s experience offers insights for global health systems aiming to reduce burnout and strengthen workforce resilience.

Prolonged stress and burnout among HCWs have serious consequences for both individual well-being and the health care system. These conditions contribute to a range of physical and mental health issues, including fatigue, anxiety, social isolation, depression, and an elevated risk of suicide [22,23]. They also adversely affect patient care, leading to decreased quality, increased medical errors, patient dissatisfaction, and reduced job satisfaction [22,24-26]. Furthermore, stress and burnout are linked to higher turnover intentions and career disengagement [22,27]. Consistent with these findings, a study in Thailand reported that job stress significantly influences physicians’ intentions to resign from their roles in health care [28]. These findings underscore the critical need for effective strategies to protect HCWs’ well-being and maintain high-quality patient care. The COVID-19 pandemic underscored the critical importance of strengthening preparedness for future pandemics and health crises. A key lesson involves the need to enhance the effectiveness and efficiency of HCWs, which aligns with the first objective of the *Global Strategy on Human Resources for Health: Workforce 2030*, issued by WHO in 2016 [29].

### Prior Work

#### Overview

A comprehensive literature review identified and analyzed existing studies on burnout and its prevention strategies, characterizing burnout as a chronic response to prolonged stress in work or academic settings. The review emphasized online learning as a key approach to mitigating burnout, highlighting the importance of providing emotional support; enhancing educator training; and using diverse, interactive tools to foster student engagement and motivation [30]. In addition, several strategies aimed at enhancing collaboration to combat stress and burnout in health care, including interprofessional education (IPE), medical movies, massive open online courses (MOOCs), and simulation-based interprofessional education (SIMBIE) incorporating co-debriefing and psychosocial support, will be discussed in detail in the following sections.

#### IPE to Enhance Collaboration as a Strategy to Combat Stress and Burnout in Health Care

Among various methodologies, such as mentorship and supervision [31,32], improving collaborative practice has demonstrated success for boosting HCW effectiveness and efficiency. Collaborative practice is when “multiple health workers from different professional backgrounds provide comprehensive services by working with patients, their families, careers and communities to deliver the highest quality of care across settings” [33]. It enhances effectiveness and efficiency by focusing on 4 key competencies: values and ethics, roles and responsibilities, communication, and teams and teamwork [34]. These competencies foster effective communication, satisfaction with high-quality teamwork, interprofessionalism, and a positive attitude with mutual respect for diverse health care disciplines [35,36]. Therefore, collaborative practice is crucial for enhancing patient safety in emergency departments [37-40]. Studies suggest that collaborative practice skills should be developed early in professional education [41-44].

IPE, where “two or more professions learn about, from and with each other to enable effective collaboration and improve health outcomes” [33], is more effective for teaching collaborative practices than other methods, such as traditional lectures [45], meeting discussions [42], video-based education [46], or simulation-based education involving only one profession [46]. IPE fosters positive perceptions of interprofessionalism through experiential learning, helping HCWs develop the 4 key competencies essential for collaborative practices. For over a decade, IPE has been integrated into university studies to promote teamwork [47,48], enhance understanding of professional roles and responsibilities [49], strengthen communication skills [50,51], and expand interprofessional knowledge [52,53].

A meta-analysis review study by Sezgin and Bektas [54] found IPE significantly improves communication competency (n=7; 95% CI 0.26 to 0.82; *P*<.001) and teams-and-teamwork competency (n=4; 95% CI 0.25 to 0.56; *P*<.001) among HCWs. However, the analysis included only 8 randomized controlled trials (RCTs)—too few for subgroup analysis of key intervention characteristics. Similarly, the meta-analysis by Marion-Martins and Pinho [55] showed IPE enhances values-and-ethics competency by fostering cross-professional mutual respect (*P*=.007; n=1) and improves roles-and-responsibilities competency (n=2; *P*=.004), yet the limited number of studies in each analysis restricts the generalizability of these findings. Overall, meta-analyses have shown that IPE interventions significantly improve health care systems (12 studies: standardized mean difference [SMD]=1.37, 95% CI 0.92 to 1.82 [56]; 6 studies: mean difference 7.19, 95% CI 2.61 to 11.77 [55]). A recent scoping review also highlighted IPE’s positive impact on organizational culture, climate, and staff attachment [57]. Additionally, IPE initiatives improve health care professionals’ work environments and strengthen multidisciplinary team effectiveness [58].

Based on previous evidence-based studies, we can conclude that IPE is an effective methodology for fostering collaborative practices, indirectly alleviating workplace stress, reducing burnout, and decreasing turnover intentions while enhancing HCWs’ well-being, career satisfaction, and perceived service quality. Despite these benefits, Frenk et al, for The Lancet Commissions [59], criticized health education curricula for being fragmented and focused on a single profession, failing to prepare students for team-based clinical practice [59]. Traditional siloed education persists, hindering collaboration and patient safety [60].

#### Medical Movies to Enhance Collaboration as a Strategy to Combat Stress and Burnout in Health Care

The integration of films and television series into medical education, a practice known as cinemeducation [61], has been demonstrated to effectively enhance learning outcomes [62,63]. Cinemeducation has been widely implemented across various disciplines, including medical diagnostics [64], nursing [65], pharmacology [66,67], psychiatry [68-70], and psychology [71,72]. Importantly, cinemeducation extends beyond the mere screening of films in classrooms; it is grounded in a structured pedagogical framework that involves a sequence of carefully designed steps before, during, and after the educational activity [73,74]. Surprisingly, to the best of our knowledge, no study has examined the direct relationship between cinemeducation and stress or burnout among HCWs. Most studies investigated the effectiveness of cinemeducation for alleviating stigma, one of the greatest barriers to mental health treatment [75-79] that indirectly contribute to levels of stress [80,81] and burnout [82,83].

The study by Zeppegno et al [84] indicated that cinemeducation has the potential to reduce stigma, foster positive attitudes toward psychiatry, and enhance students’ ability to manage anxiety when confronted with others’ distress, with effects lasting up to 6 months. The pilot study by Rehl et al [85] demonstrated the effectiveness of cinematic virtual reality (cine-VR) training—a combination of cinemeducation and VR—for reducing stigmatizing attitudes among osteopathic medical students toward patients with opioid use disorder. The study also reported increased empathy, aligning with Vygotsky’s theory of learning, which emphasizes learning through collaboration with others using reflection and authentic activities in real-life situations [86]. Similarly, the study by Kontos et al [87] used a filmed version of a research-based theatrical production to reduce caregivers’ stigmatizing toward dementia. The study by Hawke et al [88] demonstrated that film-based interventions could reduce stigma among health care service providers toward individuals with bipolar disorder, with effects persisting for 1 month. Additionally, the pilot study by Linton et al [89] found that the film “Wounded Healer” significantly reduced stigma toward mental illness among health care students. Based on the reviewed studies, there is no doubt that cinemeducation is an effective approach for reducing stigma among HCWs and students.

#### MOOCs to Enhance Collaboration as a Strategy to Combat Stress and Burnout in Health Care

MOOCs are characterized as (1) “massive,” enabling access to thousands of learners; (2) “open,” with no enrollment fees; (3) “online,” delivered via the web; and (4) “courses,” offering structured content aligned with specific learning objectives [90]. They were first introduced in 2008 by Stephen Downes and George Siemens [91]. MOOCs have since revolutionized distance learning, driven by growing demand for flexible, accessible education [92,93]. The COVID-19 pandemic further accelerated their adoption, making MOOCs a central focus in education, with over 16,300 courses offered by 950 universities and more than 180 million enrollments globally [94] on platforms such as Coursera, edX, FutureLearn, Thai MOOCs, and Udacity [95,96].

Pedagogically, MOOCs fall into 3 categories: cMOOCs (connectivist), xMOOCs (extension of something else), and iMOOCs (integrated) [97,98]. cMOOCs, based on Downes’ connectivist principles, emphasize networked learning; peer interaction; and evolving, learner-driven content [99-101]. In contrast, xMOOCs prioritize instructor-led, content-centered learning with limited interaction [102]. iMOOCs, developed by Universidade Aberta, blend the collaborative, flexible features of cMOOCs with the structured, assessed nature of xMOOCs, encouraging self-directed learning, peer engagement, and artifact-based assessment (eg, presentations, videos, mind maps) [98]. Due to their scalability and accessibility, MOOCs are highly effective for educational interventions across diverse global audiences [103]. In health care education, MOOCs serve various roles—from promoting health literacy among the public (eg, dementia education [104]) to supporting just-in-time training during health crises [105] and enhancing HCWs’ well-being and resilience against stress and burnout [106].

MOOCs are an effective platform for delivering evidence-based interventions—such as stress and burnout education and mindfulness training—to HCWs, improving resilience while reducing stress and burnout. Recently, Ricker et al [107] conducted a single-group cohort study evaluating the Physician Well-being Course, a 4.5-hour online program covering well-being fundamentals (sleep, nutrition, exercise, resilience, and mindfulness), followed by a 2-week self-selected resiliency activity (10 minutes daily). The course was offered to postgraduate year-1 residents who could join voluntarily. Among 87 enrollees, 53 (61%) completed the course, with meditation being the most frequently selected resiliency activity (36/53, 68%) and sleep being the most frequently reported wellness behavior (22/36, 61%). Postintervention assessments showed statistically significant improvements in emotional exhaustion, depersonalization, and resilience (*P*<.05, paired *t* test). The authors suggested a key strategy to obtain a relatively high completion rate was attributed to offering the course during the preresidency timing, when residents had additional time and energy. Limitations of the study were the lack of follow-up assessing long-term effects and a control group.

Peterson et al [106] conducted a single-group cohort study to evaluate a pilot program supporting nursing students with coping with stress and burnout during the COVID-19 pandemic. The intervention consisted of an 8-hour self-paced online course and a 1-hour Zoom support group addressing 7 objectives including stress recognition, crisis response, self-care planning, and psychological safety. Psychoeducational resources such as mindfulness and breathing exercises were embedded, and participants were encouraged to choose a “battle buddy” for peer connection. Of 360 enrollees, 224 completed both pre- and postcourse surveys. Significant improvements were observed in calmness, connectedness, coping capacity, and hopefulness (*P*<.001, paired *t* test), reflecting gains in psychological flexibility [108], a protective factor against mental health deterioration [109-111]. The program also enhanced a sense of agency [65] through shared experiences and personalized resilience strategies, contributing to reduced acute stress [112]. Notably, burnout risk decreased significantly (OR 0.58, 95% CI 0.4-0.9; *P*<.006), which the authors linked to reduced isolation and increased social connectedness—consistent with theoretical models that identify social support and community belonging as key protective factors [113]. However, the study lacked a control group and follow-up data to assess long-term effects.

The asynchronous nature of MOOCs enables participants to engage with content at their own convenience and pace, thereby accommodating demanding clinical schedules. Additionally, MOOCs can support virtual peer groups, which promote shared coping strategies, social connectedness, and a sense of community. These elements are critical mechanisms for fostering psychological resilience and enhancing HCWs’ sense of agency, ultimately contributing to a reduction in acute stress—even under challenging circumstances [112].

#### SIMBIE With Co-Debriefing and Emotional Psychosocial Support to Enhance Collaboration as a Strategy to Combat Stress and Burnout in Health Care

As elaborated in the IPE section, IPE is an effective method for fostering the 4 competencies essential for collaborative practices, resulting in the mitigation of stress and burnout among health care professionals. Among other educational strategies [114-118], a recent scoping review and related studies indicate that SIMBIE, defined as “when participants and facilitators from two or more professions are engaged in a simulated health care experience to achieve shared or linked objectives and outcomes” [119], is an effective method for teaching the 4 interprofessional competencies in the emergency department [120-123]. In addition to improving technical skills [118,124,125], evidence-based studies have also demonstrated that SIMBIE enhances nontechnical skills such as communication and teamwork [54,118,124-130], with effects sustained for up to 6 months [124]. This is particularly significant given the increasing recognition that nontechnical skills are critical determinants of patient safety and quality of care [131-133].

Additionally, studies demonstrated unique advantages of SIMBIE over other educational strategies for learning IPE. First, SIMBIE offers a safe [134,135], controlled [127,136], and realistic [121,137] environment where learners from various health care professions can practice collaboration and teamwork without posing any risk to real patients [44,127]. Second, SIMBIE enables repetitive and deliberate practice, allowing learners to refine their skills by learning from mistakes in a risk-free setting—something not always feasible in real clinical environments [138-143]. Third, SIMBIE facilitates active authentic experiential learning by immersing learners in realistic clinical situations that prepare them for real-world practice [137,144,145]. Unlike passive formats, substantial research in adult education arguably emphasizes that active participation significantly enhances learning effectiveness [118,146-152]. Authentic experiential learning of SIMBIE benefits its uses in stress inoculation (SIT) training. Couarraze et al [153] reported positive effects on stress, anxiety, and burnout of anesthesia and critical care workers after attending simulation training based on critical situation exposure: The effects lasted for at least 1 week. Fourth, SIMBIE can expose learners to rare and complex medical conditions that they may not encounter during typical clinical rotations [145,154], thereby equipping them to manage a broader range of clinical scenarios more effectively. Studies have shown that using simulation as a teaching strategy significantly reduces State-Trait Anxiety Inventory (STAI) scores, with this reduction persisting at a 1-week posttraining follow-up. These findings underscore the benefits of simulation-based education, particularly for residents in anesthesia and intensive care [153]. Similarly, Shamputa et al [155] highlighted the effectiveness of virtual IPE initiatives for fostering interprofessional collaboration (IPC), particularly during the COVID-19 pandemic.

Beyond economical [156], logistical [157], scalability [158], and resilience-related [159] benefits, virtual SIMBIE has shown potential for enhancing educational outcomes. A recent meta-analysis reported that virtual SIMBIE significantly improves students’ clinical reasoning and performance [39]. A systematic review and meta-analysis by Jiang et al [151] found no significant differences between virtual and real-world SIMBIE in terms of improving knowledge, procedural skills, clinical reasoning, or communication skills. Liaw et al [160] reported that desktop VR–induced stress levels of medical and nursing students, measured using blood pressure and heart rate, were comparable to the level induced by face-to-face simulation with during simulated rapidly deteriorating patient situations. Similarly, Shamputa et al [155] highlighted the effectiveness of virtual IPE initiatives at fostering IPC, particularly during the COVID-19 pandemic. Although virtual SIMBIE offers standardized and immersive practice scenarios, development of tacit knowledge for clinical practice is facilitated through debriefing [161-164]. Students consistently rated debriefing as essential to their learning experience, a finding supported by prior research [165-167]. Therefore, it is imperative that facilitators of virtual SIMBIE receive appropriate debriefing training to effectively guide these critical reflective processes [93].

Debriefing in the context of simulation-based health care education refers to “the facilitated discussion between two or more individuals in order to guide reflection and review performance, with the intent of gaining insight and understanding such that future performance is improved” [168]. Debriefing is regarded as an interactive, bidirectional, and reflective conversation that involves some degree of facilitation—whether by a facilitator, multiple facilitators (co-debriefing [169]), or the learners themselves (self-guided debriefing [170])—to support the reflective learning process [168,171,172]. When conducted effectively, debriefing thus serves as a critical component of learning in SIMBIE [163,173-175]. Its importance lies in its role as a process of reflection-on-action [176,177], which is a central element of Kolb’s [178] experiential learning cycle. Experience alone during simulation is insufficient to produce learning; rather, it is the intentional reflection on that experience that facilitates deeper understanding [164,173,174,179-182].

Attending debriefing sessions has been studied as a potential strategy for reducing stress and preventing burnout. Although a small study reported no significant effect on burnout scores, most participants appreciated the emotional and social support offered through these sessions and recommended debriefing as a beneficial approach for junior medical residents [183]. Attending debriefing sessions has been shown to significantly reduce the risk of burnout and alleviate work-related posttraumatic stress among intensive care staff, even after accounting for resilience and other contributing factors [184].

Several studies demonstrated that team debriefing after critical events provides various aspects of job and personal resources [185]: for example, (1) psychosocial support [186-193], (2) improvement in teamwork and interprofessional relationships [190,191,194-197], (3) learning and performance improvement [191,193,196-198], and (4) team-based cultural enhancement [189,190,199]. The Job Demands-Resources (JD-R) model [185] hypothesizes that the resources—both job-related (eg, knowledge, skills, abilities, social support) and personal (eg, self-efficacy)—function as a protective “buffer” that mitigates the adverse impact of job demands on individual strain, thereby reducing the risk of burnout [185,200,201].

This hypothesis is supported by both qualitative and quantitative evidence. Qualitative studies highlight the benefits of SIMBIE and debriefing interventions for enhancing emotional support and team reflection [140,186,187,190,191,193,195,202-206]. Quantitative studies further support these findings. For example, although Gunasingam et al [183] found no statistically significant reduction in burnout scores, participants valued the emotional and social support gained from debriefing sessions. Similarly, Colville et al [184] reported that debriefing was significantly associated with reduced burnout and work-related posttraumatic stress among intensive care staff, even after adjusting for resilience and other factors. These findings underscore debriefing as a time-efficient, evidence-based strategy for mitigating burnout in both simulation-based and real-world clinical environments.

### Theoretical Frameworks

#### Stress and Burnout

Stress experienced within teams engaged in collaborative practice can be broadly categorized into 2 types, based on the differential impact of stressors: individual stress and team stress [207-209]. One of the most widely accepted definitions of stress is provided by Lazarus and Folkman [210], who conceptualize individual stress as “a particular relationship between the person and the environment that is appraised by the person as taxing or exceeding his or her resources and endangering his or her well-being” [211]. Weaver et al [208] extended the definition to the team context, defining team stress as “a particular relationship between the team and its environment, including other team members, that is appraised by the team members as taxing or exceeding their resources and/or endangering their well-being.” Fundamentally, team stress represents a distinct collective psychological construct, whereas individual stress reflects a psychophysiological phenomenon experienced at the individual level [207].

Although individual stress and team stress occur at different levels, they can exert reciprocal and cross-level influences on performance at both levels [209]. Cross-level studies have revealed how specific team-level stressors—such as role ambiguity [212], team climate [213], and team conflict [214]—affect individual team members’ attitudes, behaviors, and emotional states in ways that are relevant to their performance. Conversely, studies have also demonstrated that individual-level stressors, including workload [215] and time pressure [216], can significantly impact team performance. For instance, Savelsbergh et al [217] found that excessive team-level quantitative workload negatively affects both individual and team performance by inhibiting team learning behaviors and further impairs individual performance indirectly through elevated individual workload [217]. Additionally, Kamphuis et al [218] reported that team-level intervention strategies can modulate the effectiveness of individual-level interventions aimed at enhancing individual performance. Collectively, these findings underscore that both individual performance and team performance are shaped by complex interactions between team-level stressors—reflecting characteristics of the team environment—and individual-level stressors—reflecting the attributes and experiences of individual team members.

Several studies have reported that prolonged exposure to occupational distress can ultimately lead to burnout [187,219-229]. This burnout induction process can be explained by the JD-R model [185], which is 1 of the 2 leading theoretical frameworks on burnout [230]. According to the JD-R model, burnout occurs when the availability of resources—both job-related (eg, knowledge, skills, abilities, social support) and personal (eg, self-efficacy)—is insufficient over time to buffer the negative effects of job demands on individual strain [185,200,201]. The model hypothesizes that increasing access to resources—physical (eg, assistance with wearing personal protective equipment [PPE]), psychological (eg, positive feedback from peers or supervisors), social (eg, peer support), or organizational (eg, stress-coping programs)—can help mitigate the risk of burnout.

#### Cinemeducation

Our cinemeducation was created on the basis of vicarious learning, which is the modification of an observer’s behavior that is similar to that of a model by watching the model’s behavior be reinforced or punished [231]. The effectiveness of cinemeducation stems from its ability to simplify the comprehension of complex situations by presenting real-life role-playing scenarios that illustrate both effective and ineffective practices. This approach enhances the understanding of human behaviors and their impact on colleagues and patients. Movies in cinemeducation serve as a unique and engaging learning tool [232,233], capable of stimulating debate [234] and providing insights into students’ perspectives on topics that might otherwise remain unexpressed [235-237] or be overshadowed by technical considerations [238]. Cinemeducation thus transforms implicit aspects of human behavior, teamwork, and professional interactions into explicit, tangible learning experiences, fostering deeper comprehension, critical reflection, and meaningful discussions.

Furthermore, movies effectively evoke emotions that foster active participation and deeper learning [239]. They leverage vicarious learning by allowing viewers to emotionally connect with characters [240]. Emotional experiences tend to be more vividly remembered [241-243], facilitating vicarious learning among students, a process supported by the Yerkes-Dodson law [244]. Our arguments on the effectiveness of cinemeducation have been further supported by empirical evidence suggesting that movies are effective at reducing stigma by fostering active learning through emotional engagement [245-247] and by imparting values centered on lived experiences [88]. Moreover, movies could represent a highly complex form of symbolic content that conveys nuanced and richly layered audiovisual information to students through storytelling. Beyond merely delivering content, cinema functions as both a tool and a reflective space, akin to psychotherapeutic sessions, allowing viewers to project aspects of their own psyche onto movies [248]. This process enables viewers to immerse themselves in the narrative, fostering self-reflection and deeper engagement. Additionally, cinema is believed to provide a contemporary experiential medium that momentarily detaches viewers from their daily lives, engaging their unconscious mind in a manner comparable to hypnosis and dreaming [249]. These immersive experiences contribute to the formation of subjective world views, a concept theoretically grounded in Jungian [250] and Hillmanian [251] perspectives on the relationship between images and archetypes. According to this framework, archetypal experiences—facilitated through engagement with cinematic imagery—support learning from both cognitive and emotional perspectives [252]. Such experiences encourage students to engage in discovery and self-reflection, aligning with the principles of inductive learning [253].

Cinemeducation for teaching professionalism as well as stress and burnout education was delivered to HCWs using xMOOCs. xMOOCs represent a practical and scalable solution to deliver interventions that mitigate stress and prevent burnout to health care professionals. The asynchronous nature of xMOOCs enables participants to engage with content at their own convenience and pace, thereby accommodating demanding clinical schedules.

#### SIMBIE and Co-Debriefing

##### Experiential and Phase Learning

In this study, the learning facilitated by SIMBIE and co-debriefing is grounded in experiential learning theory [178]. The experiential learning theory conceptualizes learning as an ongoing, cyclical process comprising 4 stages “whereby knowledge is created through the transformation of experience” [178]. The cycle begins with a concrete experience, which, in this context, is generated through SIMBIE, which provides a safe and controlled environment for repetitive and deliberate practice. This is followed by reflective observation and abstract conceptualization, both of which primarily happen during co-debriefing sessions. In the reflective observation stage, facilitators guide learners to examine their experiences from multiple perspectives, fostering deeper insight into the significance of their actions and decisions. These insights then serve as the foundation for abstract conceptualization, where learners attempt to synthesize and generalize their experiences into broader theories or hypotheses. The final phase, active experimentation, occurs when learners are encouraged to test their emerging understandings by engaging in subsequent simulated scenarios in SIMBIE. At this stage, learners apply their knowledge of “good” and “bad” responses, decisions, and actions to analogous situations. These new experiences then initiate another cycle of learning. Experiential learning theory underscores that meaningful learning does not arise from experience alone but rather from deliberate reflection on those experiences.

Additionally, the intervention program was developed based on the 3 sequential stages of phase learning [254], which are rooted in experiential learning theory [178] and principles of instructional scaffolding [255]: (1) prebriefing, (2) participation, and (3) co-debriefing. The prebriefing phase is defined as “a time when the facilitator illustrates the purpose of the simulation, the learning objectives, the process of debriefing, and what it entails” [172]. This phase aims to enhance learners’ engagement and involvement [256]. Effective prebriefing must communicate to learners that they are entering a controlled and purposeful environment for reflective practice, where making errors is not only accepted but expected as part of the learning process [256]. Critically, psychological safety should be established from the outset—ideally during the very first interaction between facilitators and learners [135,168,257]. In the participation phase, the virtual SIMBIE environment enables learners to acquire concrete experiences by enacting prior knowledge about social and medical skills, clinical roles, and IPC. This occurs within a setting that is safe [134,135], controlled [127,136], and realistic [121,137]. These conditions allow for repetitive and deliberate practice, which supports skill refinement through learning from mistakes in a low-risk environment—an opportunity often not feasible in actual clinical practice [138-143]. Following participation, the co-debriefing phase facilitates learners’ reflective learning through a bidirectional, interactive dialogue, often cofacilitated by multiple instructors [168,171,172]. When implemented effectively, co-debriefing serves as a critical component of learning through SIMBIE [163,173-175]. The concrete experiences gained during simulation, while necessary, are not sufficient for learning; it is the intentional reflection on those experiences that enables learners to derive deeper understanding [173]. Through this reflective process, learners engage in observation and abstract conceptualization, promoting active experimentation and eventual behavioral change—core mechanisms described in experiential learning theory [164,174,179-182].

##### Theoretical Framework for SIMBIE

In addition to constructivism, experiential learning theory, situated learning theory, and andragogy, as outlined in [258], our SIMBIE approach is also grounded in resilience theory. The term “resilience” is derived from the Latin word resilire, meaning “to leap back” [259]. Resilience has been explored across a wide range of scientific disciplines—including engineering [260], ecology [261], psychology [262], and health care [263]—resulting in varied definitions and conceptualizations [259,262]. Even within the field of psychology, perspectives differ: Some researchers define resilience as the capacity for positive adaptation in the face of significant adversity [264], whereas others view it as the ability to maintain a stable equilibrium under stress [265]. Despite these variations, most definitions converge on 2 core elements: positive adaptation and the presence of adversity [266]. Given the conceptual ambiguity, our study does not aim to redefine resilience but instead focuses on evaluating whether proposed resilience factors (such as the 4 competencies of collaborative practice) demonstrably confer resilience. To guide this inquiry, we adopted the bidimensional framework for resilience research [267], which categorizes influencing factors into 2 domains: intrinsic factors (resilience and risk factors internal to the individual or team) and external factors (protective and environmental risk factors).

One of the most compelling findings in resilience research, which informs our SIMBIE design, is the concept of the steeling effect—the idea that resilience can be cultivated through exposure to manageable risk rather than through avoidance of all adversity. There is increasing recognition that an appropriate level of exposure may be essential for organizing, calibrating, or “tuning” the adaptive systems of a unit, such as an individual [268,269] or a team [270], in preparation for future, more intense, unpredictable adversity. Crucially, the type, degree, and duration of the exposure must remain within a range that is manageable for the unit. If the exposure exceeds the unit’s adaptive capacity, the resulting impact differs depending on the nature of the unit. In individuals, unmanageable exposure may lead to the sensitizing effect, increasing vulnerability to future stressors [271]. In contrast, teams subjected to overwhelming adversity may experience a breakdown in collective identity, with members becoming increasingly individualistic and self-protective, thereby eroding team cohesion and effectiveness [272]. The steeling effect is consistent with the broader understanding that coping with, engaging in, and confronting challenges—rather than avoiding them—are fundamental to adult growth, learning, and development [273]. Persistent avoidance of adversity in individuals can result in reduced self-efficacy, low adaptive flexibility, and the stagnation of higher-order cognitive and emotional development, ultimately limiting self-actualization [273]. At the team level, avoidance may lead to increased brittleness and decreased tolerance to disruption, which can compromise safety and elevate the risk of harm during future crises [274]. Furthermore, such avoidance can hinder a team’s ability to identify and revise latent systemic threats, which, when accumulated, can significantly impair team performance [275].

##### Theoretical Framework for Co-Debriefing

Learners’ engagement in co-debriefing within this study was grounded in 3 interrelated theoretical frameworks: (1) social constructivism [276], (2) zone of proximal development [277], and (3) transformative learning theory [278]. The theory of social constructivism emphasizes the centrality of social interaction in collaborative learning. It conceptualizes learning as an active, meaning-making process shaped by learners’ engagement with lived experiences [276,279]. Meaning, in this view, is coconstructed through dialogue and shared reflection with peers. Learning is thereby characterized as active, constructive, self-controlled, social, and situational [280]. Complementing this perspective, Vygotsky’s [277] zone of proximal development further explains that learning is optimized when learners are supported in tasks slightly beyond their independent abilities, guided by a more knowledgeable other such as a facilitator. In co-debriefing, facilitators assume this role by guiding reflective discussions that help learners bridge knowledge gaps and deepen clinical reasoning and team collaboration.

Transformative learning theory emphasizes the transformation of learners’ “frames of reference”—the existing ready-made meanings for interpreting experiences [281]. These frames consist of deeply held assumptions that guide how learners perceive, understand, and respond to situations. Frenk et al [59] identified transformative learning as the most advanced of 3 successive learning levels—informative, formative, and transformative—with the latter aiming to fundamentally shift perspectives, rather than merely convey knowledge or instill professional behaviors [59]. According to Mezirow [278], transformation occurs when learners revise their frames of reference to become “more inclusive, discriminating, open, emotionally capable of change, and reflective so that they may generate beliefs and opinions that will prove more true or justified to guide action.” This transformation unfolds through 3 key processes. First, disorienting dilemmas, such as challenging simulated scenarios in SIMBIE, disrupt learners’ assumptions [278]. Second, critical reflection enables them to question and evaluate these assumptions [282], going beyond technical methods to examine underlying reasoning. Third, reflective discourse involves dialogic engagement, allowing learners to explore alternative perspectives and collaboratively seek mutual understanding in a psychologically safe environment [278]. In co-debriefing, facilitators foster this safety, encouraging open dialogue that supports both individual and collective transformation through shared reflection of their peers, making the transformation both individual and collective.

##### Psychological Safety

Co-debriefing in this study was guided by the principle of psychological safety to enhance its effectiveness and efficiency [283]. Psychological safety is recognized as a foundational—and even essential—condition for effective debriefing [120,171,284]. It fosters open dialogue by creating an environment where participants feel safe to share experiences and emotions without fear of judgment, embarrassment, or punishment [173,256,285,286]. This environment supports creativity, engagement, speaking up, and learning [283,287,288] while reducing face-saving behaviors such as withdrawal or avoidance of critique [164,284].

To foster psychological safety, facilitators use both explicit strategies, such as setting clear objectives, ensuring confidentiality, and choosing appropriate settings [289], and implicit cues, including respectful tone and open body language [284]. Quiet, private simulation spaces and thoughtful physical arrangements further support this environment [164,290]. However, co-debriefing interprofessional groups poses unique challenges due to differences in participants’ backgrounds, experiences, professional identities, and learning goals [164,291,292]. These complexities extend to co-debriefers themselves, who may also differ in training, experience, and facilitation styles [163,169,293]. Maintaining emotional and psychological safety amid such diversity can be difficult, especially when power imbalances are present [284,286].

Power dynamics—how authority and influence affect interpersonal interactions [294-296]—exist in both clinical settings [297,298] and simulations like SIMBIE [120]. If unaddressed, power imbalances may suppress participation and hinder the development of interprofessional competencies [292,299]. Despite their importance, power dynamics are often avoided in co-debriefings, possibly because the topic is perceived as taboo [164,289]. Given SIMBIE’s goal of enhancing real-world collaborative practice [33], explicitly addressing power imbalances during debriefing may strengthen psychological safety and improve learning outcomes [299]. Power imbalances may also arise among co-debriefers. Poorly managed dynamics can lead to tension, miscommunication, and perceived hierarchies [169,299]. These issues may stem from positional power (based on role) or expert power (based on knowledge) [300,301]. In practice, one debriefer may dominate discussions, interrupt others, or address only one profession, undermining the value of co-debriefing and diminishing diverse perspectives [163,302]. To fulfill SIMBIE’s interprofessional goals, co-debriefers must be mindful of these dynamics and intentionally foster equitable, respectful collaboration that models the competencies they aim to teach.

### Goal of This Study

Although prior research supports the individual benefits of cinemeducation, SIMBIE with co-debriefing, and MOOCs for emergency health care professionals, these modalities have primarily been studied in isolation. A few studies have examined integrated approaches—such as combining simulation with cinemeducation—but focused on different contexts and outcomes [74,85,303]. To the best of our knowledge, no study has integrated medical movies, MOOCs, and virtual SIMBIE with co-debriefing as a combined strategy. This study aimed to explore their integration as an IPE approach to enhance collaborative practice, mitigate stress, and prevent burnout among health care professionals. This study was intended for medical educators and other health care education professionals—including nurses, pharmacies, and allied health educators—as well as policymakers involved in health professions education and curriculum development. However, a quasi-experimental design was adopted due to practical constraints in randomizing participants within a clinical setting during the COVID-19 pandemic.

Therefore, to address these gaps in the literature and build upon the promising effects of individual strategies, the goal of this study was to evaluate the effectiveness and effect size of a multimodal learning approach—grounded in the aforementioned theoretical frameworks—that integrates medical movies, MOOCs, and either computer-based or VR-based simulation with co-debriefing, collectively referred to as Emergency Room Virtual Simulation Interprofessional Education (ER-VIPE), at improving self-reported stress levels and reducing burnout among future health care professionals. Stress and burnout were measured using the Dundee Stress State Questionnaire (DSSQ) and the Copenhagen Burnout Inventory (CBI), respectively. This quasi-experimental study compared the outcomes of the ER-VIPE intervention with alternative approaches, including (1) computer-based simulation without co-debriefing, (2) VR-based simulation without co-debriefing, and (3) medical movies and MOOCs alone. We hypothesized that the ER-VIPE multimodal learning model would be more effective at reducing stress and preventing burnout than any single-component or nondebriefing method.

## Methods

### Research Design and Setting

A quasi-experimental study with a single-blinded statistician was conducted to compare stress and burnout levels among health care professional students who were divided into 3 groups: 2 groups received novel educational interventions, and 1 group served as the control group with traditional learning methods. The study was conducted at a 1500-bed university-affiliated hospital in Bangkok, Thailand, which serves as a training site for multiple health care professional programs. A quasi-experimental design was used due to the practical challenges of randomization in a clinical setting during the COVID-19 pandemic.

### Participants and Sampling

After institutional review board approval, undergraduate clinical students from 5 health care disciplines (medicine, nursing, pharmacy, radiologic technology, and medical technology) were recruited via announcements, Line, and posters and provided informed consent. Interested students enrolled through a QR-linked Google Form. The principal investigator’s contact was provided for inquiries. Participation was voluntary and scheduled outside of regular academic activities to avoid disruption.

**Figure 1:**
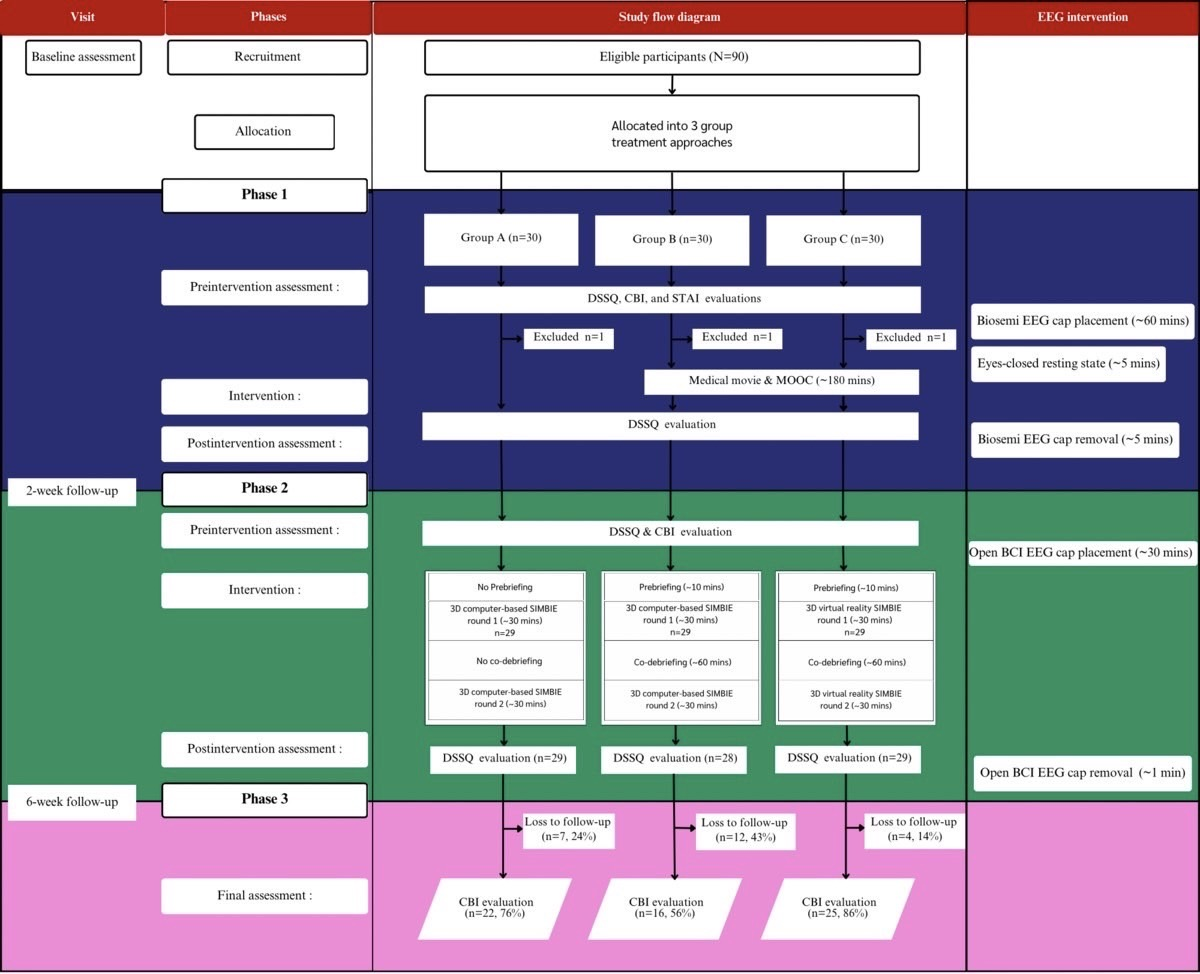
CONSORT (Consolidated Standards of Reporting Trials) diagram [305] and participant flow throughout the study, which used a 3-group treatment design. BCI: brain-computer interface; CBI: Copenhagen Burnout Inventory; DSSQ: Dundee Stress State Questionnaire; EEG: electroencephalography; MOOC: massive open online course; PHQ-9: Patient Health Questionnaire-9; RT: radiological technology student SIMBIE: Simulation-Based Interprofessional Education; STAI: State-Trait Anxiety Inventory.

Eligibility criteria included healthy individuals aged 18 years to 25 years who were enrolled as 5th-year medical students, 5th- or 6th-year pharmacy students, 4th-year medical technologist students, or 3rd- or 4th-year nursing students or radiological technologist students. The population size included approximately 533 undergraduate clinical students across 5 health care professions: medicine, nursing, pharmacy, medical technology, and radiological technology. A total of 147 students expressed interest and registered to participate in the study. Exclusion criteria included substance use (eg, smoking), a history of neurological or psychiatric disorders, Patient Health Questionnaire-9 (PHQ-9) score ≥9 [304], regular use of antidepressants, and belonging to vulnerable groups, such as pregnant individuals or students with severe illnesses. Moreover, since the potential impact of SIMBIE-induced stress was uncertain during this initial phase of the study conducted amid the COVID-19 pandemic, we prioritized participant safety. There was a concern that SIMBIE could induce stress levels exceeding the optimal arousal threshold for learning, as described by the Yerkes-Dodson law [244]. Participants were also excluded if they missed 2 scheduled appointments or failed to comply with study preparation requirements on 2 occasions (see Figure 1).

The study recruited undergraduate clinical-level students from the faculty of medicine, the institute of nursing, the faculty of pharmaceutical sciences, and the faculty of allied health sciences. Of the 90 students who initially met the inclusion criteria, 3 radiological technology students were excluded due to PHQ-9 scores exceeding the clinical threshold for depression, resulting in a final sample of 87 participants. The sample included 15 medical students, 30 nursing students, 15 pharmacy students, 15 medical technologist students, and 12 radiological technologist students. These exclusions were made to minimize potential confounding effects and ensure that observed outcomes could be more accurately attributed to the intervention.

Participants were allocated into 3 groups (A, B, and C) using a stratified convenience sampling method to ensure balance in baseline characteristics such as age, education level, and prior clinical experience. Each group was further divided into 5 interprofessional subgroups, with each subgroup consisting of 1 medical student, 2 nursing students, 1 pharmacy student, 1 medical technologist student, and 1 radiological technologist student.

Group A (control) participated in a 3D computer-based SIMBIE without oral debriefing. Group B received a medical movie, a MOOC, a 3D computer-based SIMBIE, and an oral co-debriefing session. Group C received a medical movie, a MOOC, a 3D VR SIMBIE, and an oral co-debriefing session. Stress levels were assessed using the DSSQ at 4 time points: before and after the medical movie and MOOC sessions (intervention phase 1) and before and after the SIMBIE sessions (intervention phase 2). Burnout levels were measured using the CBI at 3 time points: prior to the medical movie and MOOC sessions (intervention phase 1), prior to the SIMBIE sessions (intervention phase 2), and during the follow-up assessment conducted 4 weeks after completing intervention phase 2 (phase 3). The flow of electroencephalogram procedures is also shown in Figure 1 (also see Multimedia Appendix 1), explaining how this potential confounding factor varied across groups. All times reported were approximate.

### Data Collection

Data were collected between August 2022 and September 2023. Baseline assessments included demographic information, the DSSQ to measure stress, and the CBI to evaluate burnout as the primary outcomes. Trait anxiety was assessed once at baseline using the STAI. All questionnaires were administered online via Qualtrics. Data were collected in 3 phases. Phase 1 included DSSQ assessments before and after participants watched a medical movie and completed a MOOC, with only a pre-intervention CBI assessment conducted. Phase 2 involved DSSQ assessments before and after the SIMBIE activity, conducted 2 weeks later, along with a pre-activity CBI assessment. Phase 3 was a follow-up burnout assessment using the CBI, conducted 4 weeks after the completion of all activities. To minimize bias, data collection and analysis were performed by a single-blinded statistician. The timeline and details of the study are summarized in Figure 1.

In addition, the online survey has been reported in accordance with CHERRIES (Checklist for Reporting Results of Internet E-Surveys), which is provided as Multimedia Appendix 2. Although this study is not an RCT and thus does not require a trial registration number, we completed the CONSORT-EHEALTH (Consolidated Standards of Reporting Trials of Electronic and Mobile Health Applications and Online Telehealth; V1.6.1) submission/publication form to ensure transparency and completeness in reporting. This form is included as Multimedia Appendix 3.

### Traditional Learning Approaches

The traditional learning approach involved online, lecture-based instruction provided to health care students during the COVID-19 pandemic within a uniprofessional educational framework. These methods did not include any curriculum nor intervention specifically designed to reduce stress, build resilience, or promote interprofessional team training. Group A, serving as the control group, received no IPE exposure nor preparatory learning activities beyond the traditional lecture format. Specifically, they did not participate in lectures or cinemeducation on interprofessional collaboration, role-play activities, or pair-and-share exercises. Additionally, Group A did not undergo any preparatory learning before the simulation. Instead, they virtually engaged in a 3D computer-based SIMBIE scenario with other participants in group A and without oral co-debriefing, which served as the traditional learning condition in this study.

### Experimental Groups and Interventions

This study used a 2-arm design with Groups B and C as the experimental groups. Both groups underwent a series of interventions, including a combination of medical movie, MOOC, and 3D SIMBIE simulation, followed by a co-debriefing session, collectively referred to as ER-VIPE. Group B used a computer-based 3D SIMBIE for approximately 30 minutes, while Group C used a 3D VR SIMBIE for the same duration. Both groups participated in a 60-minute oral co-debriefing session (see Figure 1). The medical movies used in the simulations were standardized across all participants to ensure consistency in content. Similarly, the MOOC materials were delivered and maintained uniformly throughout the study to ensure consistent learning experiences. In addition, the simulation scenarios were standardized so that all participants were exposed to the same conditions and challenges, thereby minimizing potential variability.

#### Multimodal IPE Design Based on the SIT Framework

For the experimental groups, we implemented SIT [194] to induce the steeling effect through the integration of cinemeducation, coping-with-stress strategies via MOOC, SIMBIE, and co-debriefing. SIT is a structured cognitive behavioral intervention aimed at fostering resilience by equipping individuals with the tools needed to effectively manage stress. The approach consists of 3 interrelated and overlapping phases: conceptualization, skills acquisition and consolidation, and application and follow-through.

In the conceptualization phase, participants were introduced to the cognitive, emotional, and behavioral manifestations of stress using cinemeducation through medical movies and coping-with-stress strategies. A flipped classroom approach via cinemeducation and MOOC were used to optimize preparedness for the coming SIMBIE sessions [306]. Presession engagement with medical content via xMOOC platforms supported the development of interprofessional knowledge and encouraged positive attitudes IPE, Team Strategies and Tools to Enhance Performance and Patient Safety (TeamSTEPPS), and stress coping strategies [307]. This model enhanced learner engagement and emotional investment, thereby maximizing the effectiveness of in-session SIMBIE activities [308]. The integration of stress management into clinical education has been widely recognized as essential to the development of professional competencies [309].

In the skills acquisition and consolidation phase, learners developed a repertoire of coping mechanisms—including relaxation techniques; cognitive restructuring; Identify, Situation, Background, Assessment, and Recommendation (ISBAR) [49]; closed-loop communication; and collaborative communication strategies—within SIMBIE sessions that approximate real-world clinical stressors, such as time constraints and multisensory demands (visual and auditory stimuli). Skills consolidation was reinforced through co-debriefing, which provided structured opportunities for critical reflection and feedback.

In the final application and follow-through phase, learners applied these skills during a second round of SIMBIE and were progressively intensified to balance cognitive load and emotional challenge while maintaining learners within their zone of proximal development. SIT not only supports the development of self-regulation and problem-solving capabilities but also enhances self-efficacy by helping participants reframe stressors as challenges rather than threats. Moreover, it fosters a psychologically safe learning environment in which learners can engage with high-stakes situations without fear of failure—ultimately promoting more adaptive coping strategies, reduced anxiety, and improved performance under pressure.

#### Medical Movie (Cinemeducation)

Participants viewed a 75-minute medical movie developed in-house that depicted an interprofessional emergency team managing high-stress scenarios. The film emphasized communication, shared mental models, team reasoning, team support, and collaborative problem-solving for stress mitigation. Viewing was conducted individually and asynchronously, with no postfilm group discussion. Although cinemeducation is evidence-based for reducing stigma [84,85,88], its impact on stress or burnout has not been previously studied.

#### MOOCs

The MOOCs comprised 7 lessons covering essential topics, including Interprofessional Education Collaborative core competencies for IPC practice, TeamSTEPPS principles, team-based clinical reasoning for diagnosis, stress management and coping strategies, IPE for patient safety, and ethical principles for collaboration. A 15-minute segment focused specifically on stress management and coping strategies, addressing significant stressors faced by emergency department personnel, such as disease outbreaks, heavy workloads, and interpersonal challenges. These stressors were categorized as external (eg, workplace pressures and unexpected events) or internal (eg, personality traits like perfectionism and difficulties with work-life balance). The module emphasized problem-solving approaches over emotional reactions to challenges, aiming to mitigate the long-term effects of stress, including burnout symptoms like emotional exhaustion, depersonalization, and reduced personal accomplishment. All participants were required to complete both the medical movie and MOOCs as well as pass the pre- and posttest exams to receive a certificate and meet the eligibility criteria for the flipped classroom before attending the SIMBIE session.

#### Prebriefing Design

To promote psychological safety, we implemented structured prebriefing strategies based on best practices [135,168,257]. Facilitators and learners introduced themselves, shared prior experiences, and established ground rules emphasizing confidentiality, active participation, and a focus on performance improvement rather than individual critique [162,171,172,284]. Effective prebriefing must communicate to learners that they are entering a unique, controlled environment for reflective practice where making errors is acceptable and expected as part of the learning process [256].

A “fiction contract” was cocreated to encourage engagement and suspend disbelief despite simulation limitations [310-312]. Learners were oriented to the simulation space, equipment, and environment to reduce anxiety and enhance participation [182,313-315].

For co-debriefing, facilitators conducted prebriefing discussions to align objectives, align areas of expertise, clarify roles, agree on debriefing methods, clarify who will lead different elements, and address potential challenges [168,169]. This precoordination ensured a smooth, cohesive, and collaborative debriefing process and helped establish a shared mental model between co-debriefers [169]. Facilitators also attended to implicit cues—such as tone, expressions, and body language—to foster trust and a positive psychological climate [316-318].

#### 3D SIMBIE Design

The 3D SIMBIE simulations, available in both computer-based and VR formats, were designed to progressively increase in complexity and stress, replicating high-acuity emergency scenarios reflective of real-world challenges during the COVID-19 pandemic. Each simulation integrated both technical and TeamSTEPPS-based nontechnical learning objectives in the context of a multidisciplinary emergency department encounter. Participants worked as a 6-member health care team to diagnose and manage a complex case involving a 70-year-old male patient with COVID-19, chronic obstructive pulmonary disease, hypertension, diabetes mellitus, and a documented allergy to ceftriaxone. The presence of a distressed spouse added an emotionally charged layer to the scenario, requiring participants to apply both clinical and interpersonal skills.

**Figure 2:**
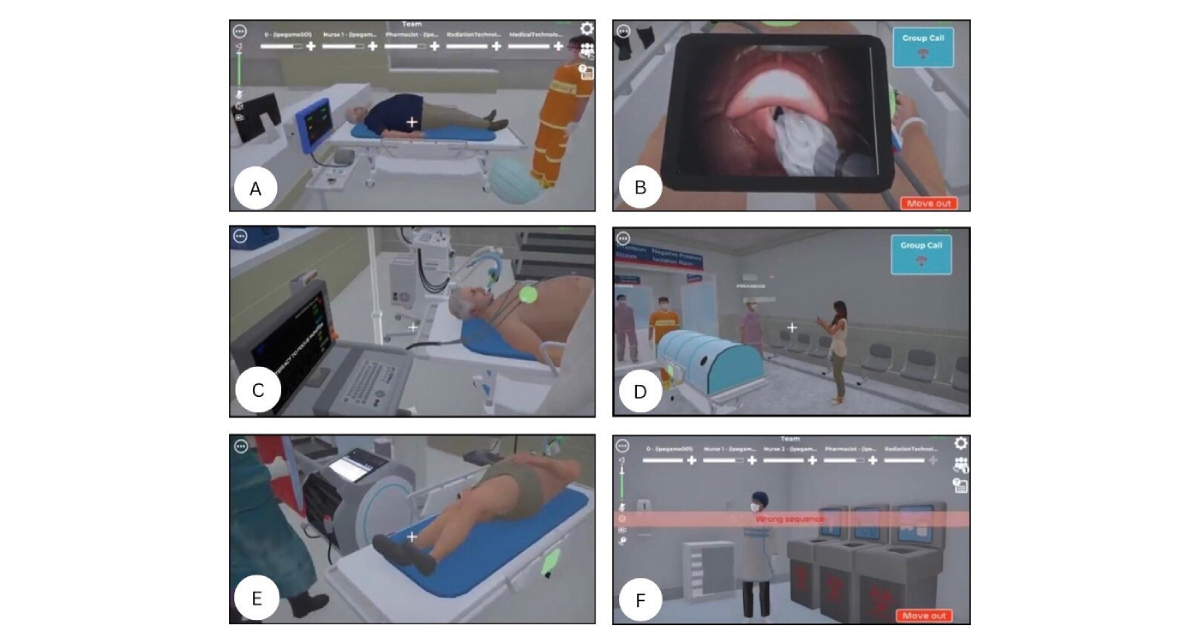
Health point (HP) bar's features, functions, and examples of stressful situations encountered by health care professionals while practicing stress coping strategies in 3D computer-based simulation-based interprofessional education (SIMBIE): (A) triage nursing student entering for patient assessment, walking in, putting on a mask, assessing the patient, and measuring vital signs; (B) medical student struggling with unsuccessful endotracheal tube insertion due to a difficult airway; (C) nursing student connecting a patient to a ventilator; (D) a pharmacy student advising a patient’s family against taking photos, emphasizing patient privacy and ethical confidentiality; (E) radiological technologist and nursing students collaborating to reposition the patient for a chest X-ray while monitoring for accidental dislodgment of the endotracheal tube; and (F) medical technologist student improperly removing PPE after working in a lab where COVID-19 was detected, triggering a system warning for the wrong sequence.

**Figure 3:**
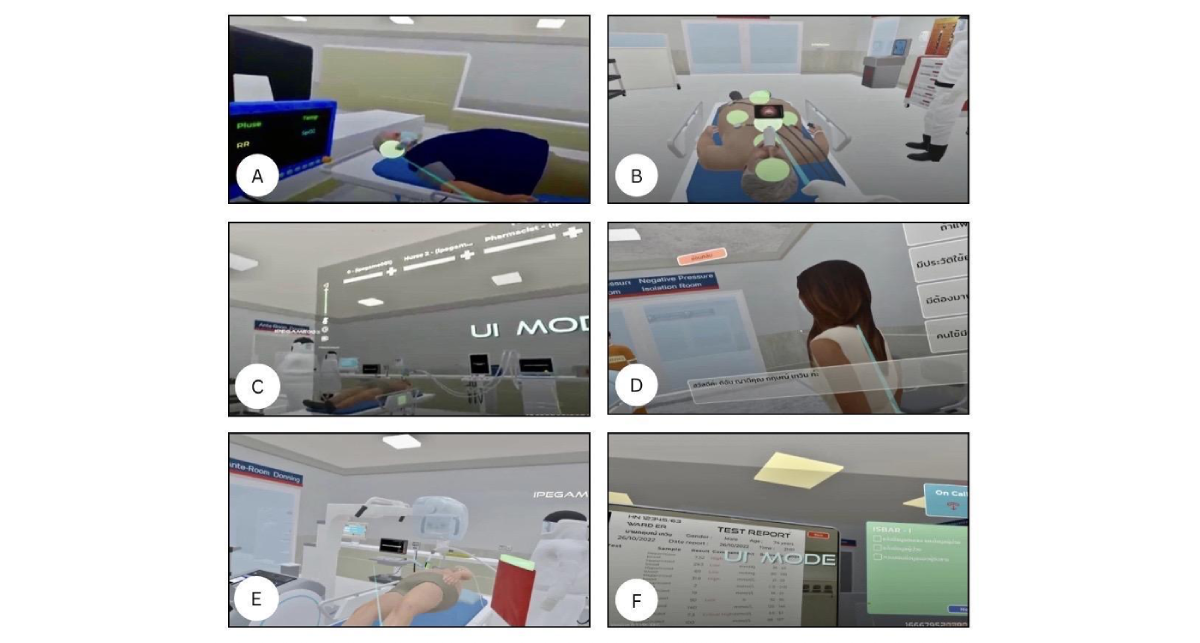
Health point (HP) bar’s features and functions and examples of stressful situations and professional perspectives encountered by 6 health care professionals while practicing Team Strategies and Tools to Enhance Performance and Patient Safety (TeamSTEPPS) and stress-coping strategies in a 3D virtual reality (VR)–based simulation-based interprofessional education (SIMBIE) scenario: (A) nursing student perspective during initial triage, including vital sign measurements, such as temperature using a green laser pointing at the ear; (B) medical student perspective while performing endotracheal intubation using a video laryngoscope; (C) nursing student perspective while receiving a phone call from a pharmacy student alerting the team to a drug allergy and each profession’s HP bar; (D) pharmacy student perspective while politely warning a patient’s relative taking a photo of the patient and clinical staff; (E) radiologic technologist student view while assisting the nursing student with repositioning the patient to safely remove a lead X-ray plate, ensuring proper side rail placement to prevent accidental extubation or falls; and (F) medical technologist student perspective while urgently communicating a critical potassium level using the Identify, Situation, Background, Assessment, and Recommendation (ISBAR) communication framework to the team via phone.

Technical training included time-sensitive interventions such as intubation, ventilator management, laryngeal mask airway insertion, cricothyroidotomy, hyperkalemic crisis, use of PPE, and coordination of portable chest imaging. Clinical reasoning was guided toward formulating an accurate diagnosis and implementing a safe treatment plan under pressure, closely mirroring the demands of real-world emergency care clinical environments and emphasizing decision-making under pressure, as illustrated by the multiple stressor events shown in Figures 2 and 3. Nontechnical training focused explicitly on the 5 TeamSTEPPS domains—team structure, communication, leadership, situation monitoring with the STEP framework, and mutual support—using strategies such as ISBAR, closed-loop communication, and real-time psychosocial support. Communication occurred via open microphones to simulate authentic interprofessional interaction, and learners engaged with the patient’s spouse through a branching dialogue system that enabled them to select empathetic responses in real time.

An interactive game-based interface further reinforced TeamSTEPPS competencies as our main learning objectives. Each participant was equipped with a HP bar, a symbolic “health point” indicator representing emotional resilience under pressure. The HP bar depletes over time, particularly during high-stakes decision points or delays in clinical action such as managing a “can’t intubate, can’t ventilate” situation, video laryngoscope battery failure, or sudden hypotension**.** Mutual support was emphasized as teammates could replenish each other’s HP by pressing a “plus” button—an interactive metaphor for peer support and collaborative coping. The HP bar thus served both as a visual representation of stress load and as a positive reinforcement mechanism for TeamSTEPPS-aligned behaviors. These features were designed to enhance psychological fidelity and simulate cognitive load realism by integrating both clinical complexity and emotional dynamics.

This study was conducted during the COVID-19 pandemic, during which all learners were limited to online lectures without clinical exposure. Given these constraints, along with the logistical challenges of assembling multiprofessional student teams and facilitators, we designed the study to ensure both research feasibility and standardization. To address safety concerns for both participants and researchers, a control group (Group A) was exposed to a 3D computer-based SIMBIE simulation without debriefing. All participants provided informed consent after being fully briefed on the study’s potential risks and benefits. Inclusion criteria required that participants have a PHQ-9 score within the normal range, to minimize psychological risk. Trained research assistants were present throughout the simulation to observe and provide support as needed. Participants were also informed of their right to withdraw from the study at any time without penalty. These measures were implemented to uphold ethical standards and ensure fairness, psychological safety, and relevance to the context of the pandemic.

The 3D SIMBIE and 3D VR SIMBIE platforms were developed by our ER-VIPE team, which comprises professionals from various disciplines including emergency physicians, nurses, pharmacists, medical technologists, radiologic technologists, communication arts specialists, instructional designers, architects, psychologists, and experts in the humanities and education. This interdisciplinary team collaborated with a specialized group of engineers in immersive learning technologies for health care education. Both platforms use advanced simulation software to create realistic, interactive environments that enable participants to engage in high-stakes medical scenarios.

The key distinction lay in the level of immersion: The 3D version used standard computer input devices (eg, screen, keyboard, and mouse), while VR SIMBIE used headsets and controllers to deepen emotional engagement and presence. This allowed participants to physically move and interact within the environment in a fully immersive and realistic manner. The VR format enhances the sense of presence, making participants feel as though they are “inside” the clinical scenario, which may result in greater cognitive and emotional stress compared with the 3D desktop version. Figures 2 and 3 illustrate the user interface, visual comparisons, and experiential differences between both platforms. In addition, they show the health point (HP) bar, which can reach a maximum of 50 points, with the ability to restore a teammate’s energy up to 5 times, adding 10 points each time. Each simulation concluded with a structured co-debriefing session, enabling participants to reflect on clinical decision-making and TeamSTEPPS performance, consolidating targeted nontechnical teamwork competencies.

#### Co-Debriefing Design

Following the initial 30-minute SIMBIE session, participants engaged in a 60-minute oral co-debriefing session, guided by TeamSTEPPS as the primary learning framework. This session provided a safe, nonjudgmental environment for team-based problem-solving and crisis management. Facilitated by instructors, it aimed to enhance experiential learning, reinforce team dynamics, and foster a no-blame culture. After the co-debriefing, participants in Group B replayed the 3D computer-based SIMBIE for a final 30-minute session, while participants in Group C replayed the 3D VR SIMBIE for their concluding 30-minute session.

In this study, co-debriefing was conducted using two complementary conceptual frameworks designed to optimize critically reflective learning: (1) the PEARLS (Promoting Excellence and Reflective Learning in Simulation) framework [169] and (2) the “divide and conquer” approach [169]. The PEARLS framework offers a straightforward and structured method for facilitating consistent, reliable exchanges of information during co-debriefing. It involves 4 sequential phases: reactions, during which learners can voice emotionally salient concerns or choose discussion points; description, during which facilitators and learners establish a shared, factual account of what happened; analysis, which explores performance gaps and encourages critical discussion; and summary, which consolidates the discussion into key learning points.

The reaction phase helps to establish a shared and psychologically safe foundation for learning and reflection by allowing learners to express their immediate thoughts and emotional responses [315]. For instance, facilitators began with questions such as, “What emotions are you experiencing right now?” to validate participants’ initial reactions and set a supportive tone for reflection. The inclusion of this phase not only supports psychosocial well-being but also may enhance learner engagement by increasing the relevance and resonance of subsequent discussions. In the description phase, debriefers introduce observations coupled with evaluative judgments and may shorten the phase if learners appear to have a shared understanding of the case. This is followed by the analysis phase, in which facilitators guide group reflection using structured prompts. Examples of such prompts included: “Can you describe what was on your mind during the most intense moments?” “In what ways did your team interact and support each other under pressure?” “What specific actions did you take to address the issue, and how successful were they?” and “Are there any other methods you think might have worked in that situation?” Facilitators also applied the plus/delta technique, asking questions such as “What aspects of the scenario went smoothly?” and “What elements could be improved next time?” In addition, the advocacy-inquiry model was used to promote deeper insight into decision-making. For example, a facilitator might say “I’d like to talk about mutual support in TeamSTEPPS. I observed three unsuccessful intubation attempts. I was concerned about the effect of prolonged hypoxia. What was going through your mind as your team worked to establish the airway?”—encouraging participants to share their thought processes and mental models. Finally, the summary phase concluded the session with a review of critical insights. Facilitators asked learners to identify key lessons they planned to apply in future clinical practice, such as, “What are the main takeaways from this experience that you would carry into real-life situations?”

Additionally, we implemented the “divide and conquer” approach during co-debriefing, assigning a lead role—typically the medical debriefer—to guide the session [59,163,319]. Under this model, co-debriefers conveniently divide thematic responsibilities based on the TeamSTEPPS framework, thereby minimizing redundant cognitive processing. This division of labor helps reduce the intrinsic and extraneous cognitive load experienced by co-debriefers—a key consideration in accordance with cognitive load theory, which posits that reducing unnecessary mental burden enhances co-debriefers’ performance and consequently learning outcomes of learners [311,320].

To prevent power imbalances among co-debriefers, we followed strategies suggested by Oriot et al [293] on agreeing in advance about the techniques, approaches, and educational strategies we will use—including our use of nonverbal communication. This is important because, as Holmes and Mellanby [164] noted, “debriefers debrief slightly differently, everybody has a slightly different focus, different speeds and different process for how you do it.” Establishing nonverbal communication allows co-debriefers to “authorize” each other to speak using pre-agreed signals. This approach helps prevent interruptions or conflicts, ensuring that one debriefer does not undermine the other’s process. Additionally, we also conducted a “post-debriefing huddle,” which includes peer coaching and provides an opportunity for co-debriefers to review challenges, resolve misunderstandings, and collaboratively develop strategies for future sessions [168,290].

### Outcome Assessments

To assess outcomes, DSSQ and CBI were administered. Additionally, Anxiety Trait scores from the STAI were measured as a control variable. Anxiety Trait scores were assessed prior to each intervention. Participants self-reported on the DSSQ before and after each intervention (4 total measurements per participant). Burnout assessments were conducted before each intervention and followed up 1 month later (3 measurements per participant). The e-survey was developed and administered using the Qualtrics platform (see Multimedia Appendix 4).

DSSQ and CBI were selected based on their strong psychometric properties and suitability for use with health care professionals in short-term interventions. The DSSQ provides a multidimensional assessment of psychological stress, capturing emotional, cognitive, and motivational states, and is particularly suited for evaluating acute stress responses. We selected the CBI because it is more applicable to the context of our study population—health care students undergoing both clinical and academic training. The CBI is particularly relevant for assessing 3 core dimensions of burnout: work-related, personal, and client-related. Unlike the Maslach Burnout Inventory, which was originally developed for use with long-term professionals in workplace settings, the CBI offers a broader conceptualization of burnout that includes personal exhaustion—an important consideration for student populations. Designed specifically for health care contexts, the CBI has demonstrated high reliability and validity across diverse clinical settings. Furthermore, it has shown greater sensitivity than the Maslach Burnout Inventory, with studies reporting higher burnout detection rates (53% vs 35%) [321]. This suggests that the CBI may be more effective at identifying early or less overt symptoms of burnout, aligning well with our goal of preventive intervention among students. It is considered a reliable tool across cultures and has been associated with future risks such as absenteeism, sleep issues, painkiller use, and intention to leave. Its subscales also reflect meaningful changes in burnout over time [322].

These instruments were chosen over other tools due to their brevity, sensitivity to short-term changes, and practical applicability within the study’s timeframe. Both scales are concise, enabling efficient administration without overburdening participants—an important consideration during pandemic-related disruptions and online learning contexts. Although both tools are self-reported and may be subject to response bias, they offer a balanced trade-off between feasibility and diagnostic utility. The DSSQ may not fully capture stress under extreme crisis conditions, and the CBI may not reflect long-term burnout trajectories; however, their capacity to assess immediate changes in stress and burnout levels makes them appropriate for evaluating the short-term impact of the ER-VIPE intervention.

#### A Measure of Anxiety Trait

The subscale of trait anxiety from the short version of the Spielberger STAI [323] was ordered and paid for with the necessary permissions obtained. It is used to assess an individual propensity to anxiety. Trait anxiety data were collected and included as a control variable in the analysis. There are 5 items, and the items use a 4-point Likert scale (1=not at all to 4=very much so). An example is, “In general, I feel that difficulties are piling up so that I cannot overcome them.” The Cronbach α of the original scale was found to be acceptable (α=0.91). This measure was translated into Thai using a back-translation method by J Chavanovanich, PhD (email, August 21, 2024; see Multimedia Appendix 4).

#### A Measure of State Stress

The short-version DSSQ [324] was used to assess the level of subjective stress state. Professor Helton granted us permission for its use. The DSSQ is a 24-item multidimensional measurement comprising task engagement, distress, and worry. The items use a 5-point Likert scale (1=not at all to 5=extremely). An example item is, “I was motivated to do the task.” DSSQ is a well-established tool for assessing acute stress, recognized for its reliability and validity. It consistently achieves high internal consistency, with Cronbach α for the original scale found to be acceptable, exceeding 0.80 across all dimensions. This measure was translated into Thai using a back-translation method by J Chavanovanich, PhD (email, August 21, 2024). We chose psychological tools, such as the DSSQ, instead of physiological measures like heart rate and heart rate variability because they allow for more detailed differentiation between engagement (items: 2, 5, 11, 12, 13, 17, 21, and 22), distress (items: 1, 3, 4, 6, 7, 8, 9, and 10), and worry (items: 14, 15, 16, 18, 19, 20, 23, and 24), offering a comprehensive understanding of stress states (see Multimedia Appendix 4).

#### A Measure of Burnout

A 6-item personal burnout subscale from the CBI [322] was used to assess the level of prolonged physical and psychological exhaustion. The items use a 5-point Likert scale (1=never/almost never to 5=always). An example item is, “How often are you physically exhausted?” The measure was translated into Thai using a back-translation method by J Chavanovanich, PhD (email, August 21, 2024). Permission to translate and use the CBI for this research was granted by the National Research Centre for the Working Environment by T Clausen, PhD (email, June 28, 2022). The CBI has demonstrated strong psychometric properties across various studies. It exhibits high internal consistency, with Cronbach α coefficients typically exceeding 0.90 for both the overall scale and its subscales (personal, work, and patient burnout). Construct validity was supported by confirmatory factor analysis, and convergent validity was demonstrated through strong correlations with a previously validated measurement [322,325-327]. We focused exclusively on personal burnout, as the participants were students who were neither working nor patients. Concentrating on personal burnout was expected to provide a more direct response to the research question and increase the likelihood of successful study completion, although this may reduce validity (see Multimedia Appendix 4).

### Statistical Analysis

#### Differences and Changes Over Time

Descriptive statistics were summarized using means (SDs) and medians (IQRs). Frequencies and percentages were used for categorical data. Comparisons between groups in demographic characteristics at baseline were conducted using the Wilcoxon Mann-Whitney *U* test, Kruskal-Wallis *H* test with the Dunn post hoc test, chi-square test, or Fisher exact test, as appropriate. Generalized estimating equations (GEEs) were used to analyze changes over time and assess differences in improvement scores for burnout and DSSQ between groups, with adjustments for anxiety. DSSQ engagement, distress, and worry scores were measured using specific items from the DSSQ. Engagement scores among Groups A, B, and C were obtained from items 2, 5, 11, 12, 13, 17, 21, and 22. Distress scores were derived from items 1, 3, 4, 6, 7, 8, 9, and 10, while worry scores were obtained from items 14, 15, 16, 18, 19, 20, 23, and 24. To assess changes over time and compare improvements in burnout and DSSQ scores between groups, GEEs were used, with adjustments for anxiety as a covariate. Both an intention-to-treat (ITT) analysis and a per-protocol (PP) analysis were performed. Missing data were imputed using the last observation carried forward method. Statistical significance was set at a 2-tailed *P* value of <.05 for all analyses. Stata version 15 (Stata Corp) [328] was used for data analyses.

#### Effect Size Calculation for Burnout Reduction

To assess the magnitude of intervention effects on burnout reduction, Cohen *d* was calculated for each of the 5 intervention conditions using mean differences and pooled SDs. Five effect sizes were computed according to the structure of the study. A negative effect size indicated a reduction in burnout compared with baseline, while a positive effect size indicated an increase in burnout.

##### I. Effect Size of Medical Movies and MOOCs

To evaluate the effect of medical movies and MOOCs on burnout after 2 weeks, we calculated the mean difference in burnout scores between phase 2 (pre-SIMBIE) and phase 1 (before medical movies and MOOCs) within Group B and C, using pooled SDs. An alternative comparison using Group A as a control group was also considered to assess whether burnout reduction occurred over 2 weeks without movie and MOOC exposure, accounting for potential external factors.

##### II. Effect Size of Computer-Based SIMBIE

To evaluate the effect of the computer-based SIMBIE simulation after 4 weeks, we calculated the mean difference in burnout scores between phase 3 (4 weeks post-SIMBIE) and phase 2 (pre-SIMBIE) within Group A using pooled SDs.

##### III. Effect Size of VR-Based SIMBIE

To evaluate the effect of VR-based SIMBIE simulation after 4 weeks, we calculated the mean difference in burnout scores between phase 3 and phase 2 within Group C (after follow-up losses) using pooled SDs.

##### IV. Effect Size of ER-VIPE (Computer-Based Version)

To evaluate the full ER-VIPE program (medical movies + MOOCs + computer-based SIMBIE with co-debriefing) after 6 weeks, we calculated the mean difference in burnout scores between phase 3 (6 weeks post-SIMBIE) and phase 1 (before medical movies and MOOCs) within Group B using pooled SDs.

##### V. Effect Size of ER-VIPE (VR-Based Version)

To evaluate the VR-based version of ER-VIPE (medical movies + MOOCs + VR-based SIMBIE with co-debriefing), the same approach was used. Burnout scores at phase 3 and phase 1 were compared within Group C using pooled SDs.

#### Sample Size Calculation

A power analysis for a repeated measures multivariate analysis of variance (MANOVA) conducted in G*Power (version 3.1.9.4) with a within-between interaction indicated a required sample size of 82 participants to detect an effect size of 0.35, with an α level of .05 and a power of .80. To account for potential dropout, 10% was added, resulting in a total sample size of 87 participants.

### Ethical Considerations

The study protocol was approved by the Ethics Committee for Research in Human Subjects of the Faculty of Medicine, Chulalongkorn University, Thailand (IRB number 0366/65). Participants were informed about the study’s objectives, procedures, potential risks, and benefits. Each participant received US $30 in recognition of their time and contribution, in accordance with institutional review board approval. This information was provided both orally and in writing before obtaining informed consent. Participants were assured of their right to make voluntary decisions and withdraw from the study at any time. Data were anonymized to maintain confidentiality.

## Results

### Demographics

A total of 87 undergraduate clinical students from various professional programs participated in the study, with 29 students in each group (A, B, and C). The sample was predominantly female (62/87, 71%) with a mean age of 21.87 (SD 1.13) years. No significant differences were found in demographic characteristics between the groups. However, analysis of the debriefing duration revealed significant differences between Group B (mean 50.93, SD 12.61) and Group C (mean 60.33, SD 9.75), as shown in Table 1.

**Table 1:** Demographics by treatment group.

Factor			Total sample		Group A^a^		Group B^b^		Group C^c^		*P* value	
**Gender, n (%)**												.80^d^
	Female	62 (71)		22 (76)		20 (69)		20 (69)				
	Male	25 (29)		7 (24)		9 (31)		9 (31)				
Age (years), mean (SD)			21.87 (1.13)		21.83 (0.93)		21.86 (1.03)		21.93 (1.41)		—^e^	
Age (years), median (IQR)			22 (21-22)		22 (21-22)		22 (21-22)		21 (21-22)		.90^f^	
**Academic year, n (%)**												.63^g^
	Third year	12 (14)		3 (10)		7 (24)		2 (7)				
	Fourth year	47 (54)		17 (59)		13 (45)		17 (59)				
	Fifth year	18 (21)		6 (21)		5 (17)		7 (24)				
	Sixth year	10 (11)		3 (10)		4 (14)		3 (10)				
Academic grade, mean (SD)			3.23 (0.38)		3.24 (0.40)		3.23 (0.43)		3.22 (0.31)		—	
Academic grade, median (IQR)			3.24 (3-3.50)		3.25 (3-3.53)		3.23 (3.03-3.50)		3.24 (3-3.48)		.92^f^	
Debriefing duration (minutes), mean (SD)			54.96 (12.29)		—		50.93 (12.61)		60.33 (9.75)		—	
Debriefing duration (minutes), median (IQR)			57 (45-63)		—		49 (45-58.50)		63 (60-69)		.01^h^	
Debriefing staff, mean (SD)			5.33 (1.75)		—		5.69 (1.85)		4.97 (1.59)		—	
Debriefing staff, median (IQR)			6 (4-6)		—		6 (4-7)		6 (5-6)		.06^h^	

^a^Group A (control) participated in 3D computer-based simulation-based interprofessional education (SIMBIE) without oral debriefing.

^b^Group B received a medical movie, a massive open online course (MOOC), a 3D computer-based SIMBIE, and an oral co-debriefing session.

^c^Group C received a medical movie, a MOOC, a 3D virtual reality SIMBIE, and an oral co-debriefing session.

^d^Chi-square test.

^e^Not applicable.

^f^Kruskal-Wallis *H* test.

^g^Fisher exact test.

^h^Wilcoxon Mann-Whitney *U* test.

### Baseline Outcomes and Time Interval Assessments Across Groups

Baseline assessments of burnout and DSSQ measures (engagement, distress, and worry) showed no significant differences among Groups A, B, and C (see Table S1 in Multimedia Appendix 5). Similarly, the mean interval between the postintervention DSSQ assessment in phase 1 and the pre-intervention DSSQ assessment in phase 2 did not differ significantly across groups. However, a significant difference emerged in the mean burnout assessment interval between the postintervention assessment in phase 2 and the final assessment in phase 3: Group A (mean 33.82, SD 12.90 days) differed significantly from Group B (mean 48.20, SD 23.48 days) and Group C (mean 37.20, SD 6.52 days). See Table S2 in Multimedia Appendix 6.

### Overall Assessment Outcomes Among Groups

**Figure 4:**
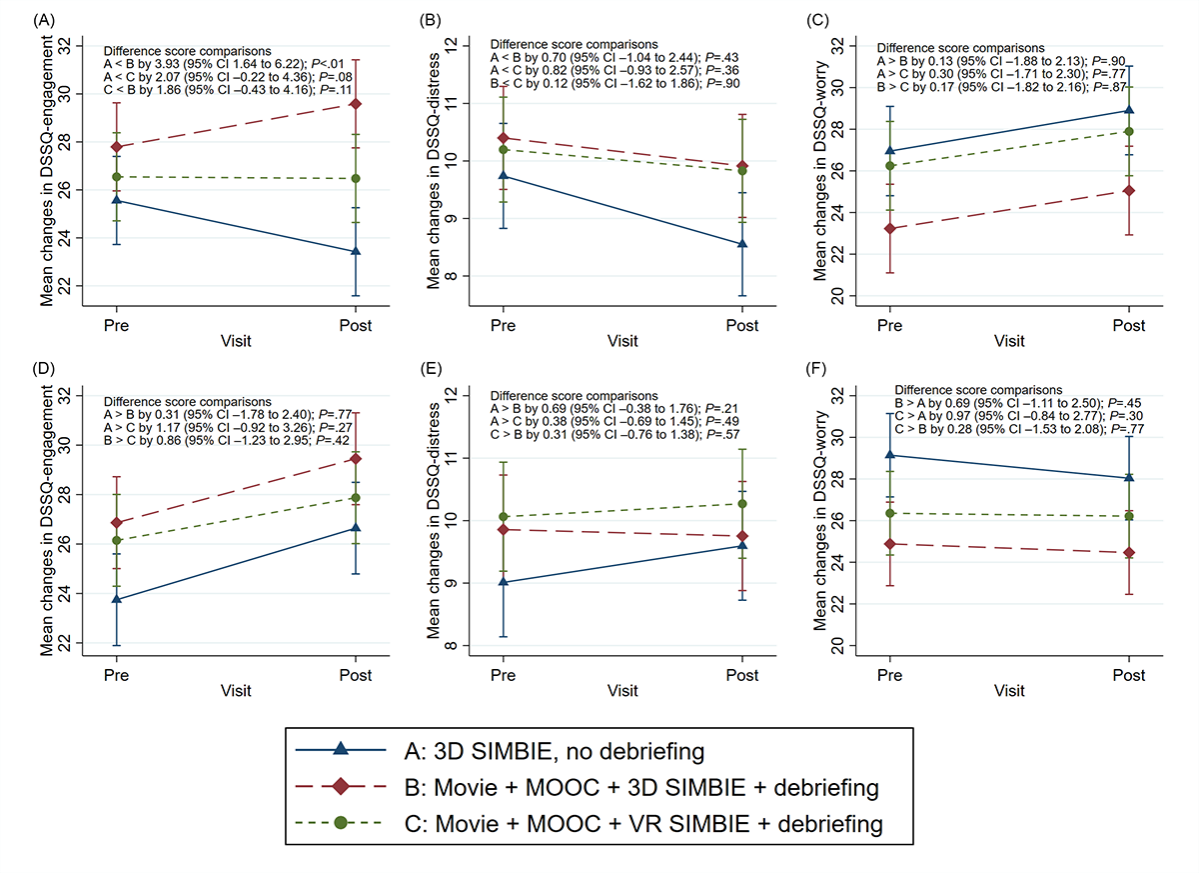
Based on intention-to-treat analysis, changes in (A) Dundee Stress State Questionnaire (DSSQ)-engagement scores, (B) DSSQ-distress scores, and (C) DSSQ-worry scores with the movie and massive open online course (MOOC) intervention as well as changes in (D) DSSQ-engagement scores, (E) DSSQ-distress scores, and (F) DSSQ-worry scores with the simulation-based interprofessional education (SIMBIE) intervention. VR: virtual reality.

**Figure 5:**
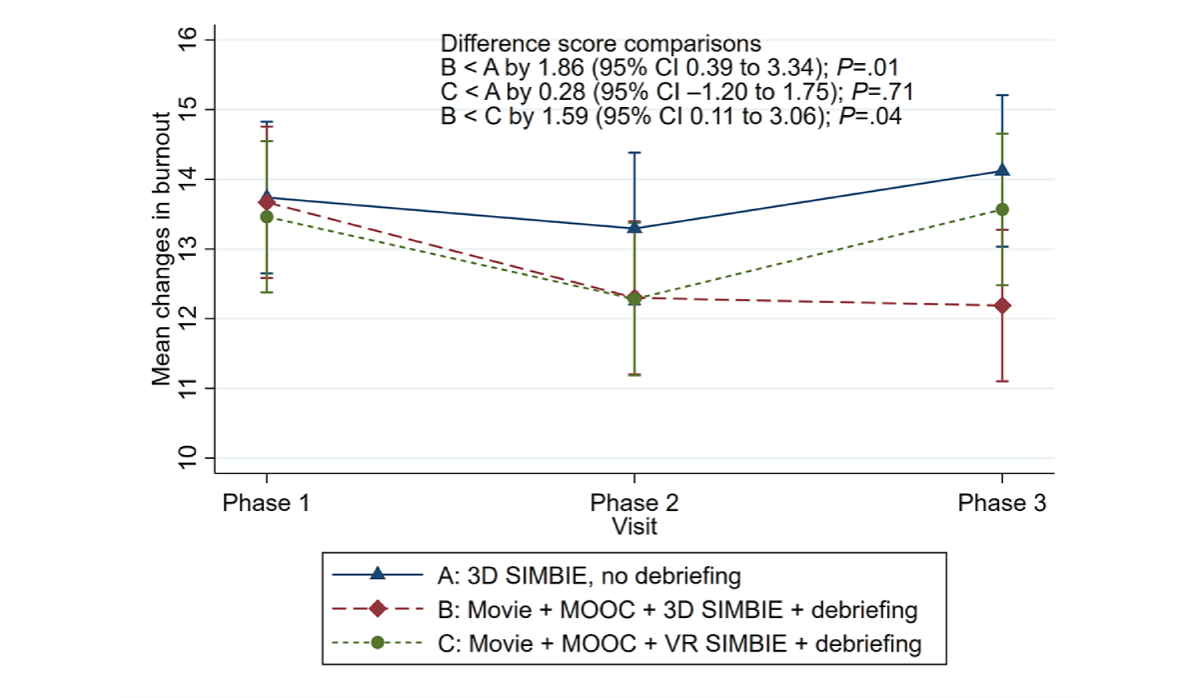
Changes in Copenhagen Burnout Inventory (CBI) scores based on intention-to-treat analysis using generalized estimating equations (GEEs), adjusted for anxiety traits as a control variable. MOOC: massive open online course; SIMBIE: simulation-based interprofessional education; VR: virtual reality.

The study aimed to assess individual state stress, as measured using the mean DSSQ scores, and burnout outcomes, as measured using the CBI, across Groups A, B, and C. Group A (control) participated in a 3D computer-based SIMBIE without oral debriefing. Group B received a medical movie, MOOC, 3D computer-based SIMBIE, and oral co-debriefing session. Group C received a medical movie, MOOC, 3D VR SIMBIE, and oral co-debriefing session. Statistical analysis was conducted using a GEE approach, with adjustments for confounding factors such as anxiety trait. Measurements were taken during 3 phases, as illustrated in Figure 1: phase 1 (movie and MOOC intervention), phase 2 (SIMBIE intervention), and phase 3 (6-week follow-up). These results were obtained through an ITT analysis (see Figure 4, Figure 5, and Table S3 in Multimedia Appendix 7). A PP analysis further corroborated the findings from the ITT analysis, demonstrating consistent results and interpretations for the mean change in DSSQ scores (see Table S4 in Multimedia Appendix 8, Figure S1 in Multimedia Appendix 9, and Figure S2 in Multimedia Appendix 10).

### Measuring State Stress With the DSSQ

#### Overview

The DSSQ [329] was selected as the primary tool in this study due to its ability to assess momentary stress states across 3 dimensions: task engagement, distress, and worry. Designed for real-time application, the DSSQ is particularly well-suited for simulation-based educational research [330]. It enabled the detection of subtle, immediate changes in learners’ stress responses before and after each ER-VIPE session. In contrast, the Perceived Stress Scale-10 [331] is a globally recognized instrument widely used in health care studies. It provides a reliable measure of general perceived stress over the past month and is appropriate for large-scale screening.

#### DSSQ-Engagement Scores Among Groups

Following exposure to a medical movie and MOOCs, Group B had significantly higher DSSQ engagement scores, adjusted for confounding factors, than Group A (mean difference 3.93; *P*<.001). Group C had nonsignificantly higher scores than Group A (mean difference 2.07; *P*=.08), and nonsignificantly lower scores than Group B (mean difference –1.86; *P*=.11). After participating in the SIMBIE process all 3 groups demonstrated positive trends in DSSQ engagement scores, adjusted for confounding factors. Group B scored higher than Group A, though the difference was not significant (mean difference –0.31; *P*=.77). Similarly, Group C had nonsignificantly higher scores than Group A (mean difference –1.17; *P*=.27) and nonsignificantly lower engagement scores than Group B (mean difference –0.86; *P*=.42). See Figure 4, Table S3 in Multimedia Appendix 7, Table S4 in Multimedia Appendix 8, and Figure S1 in Multimedia Appendix 9.

#### DSSQ-Distress Scores Among Groups

Focusing on DSSQ-distress following exposure to a medical movie and MOOCs, the results indicated negative trends across all 3 groups. Group B exhibited nonsignificantly higher distress scores than Group A, with a mean difference of 0.70 (*P*=.43). Similarly, Group C had nonsignificantly higher distress scores than Group A, with a mean difference of 0.82 (*P*=.36) and nonsignificantly lower distress scores than Group B, with a mean difference of 0.12 (*P*=.90). After participating in the SIMBIE process and adjusting for confounding factors, only Group B exhibited negative trends in DSSQ distress scores. However, the differences for all 3 groups were not significant. Group B scored higher than Group A, but the mean difference was not statistically significant (mean difference –0.69; *P*=.21). Similarly, Group C had nonsignificantly higher scores than Group A (mean difference –0.38; *P*=.49) and nonsignificantly lower scores than Group B (mean difference 0.31; *P*=.57). See Figure 4, Table S3 in Multimedia Appendix 7, Table S4 in Multimedia Appendix 8, and Figure S1 in Multimedia Appendix 9.

#### DSSQ-Worry Scores Among Groups

Regarding DSSQ-worry following exposure to a medical movie and MOOCs, all 3 groups exhibited positive trends. Group B had nonsignificantly lower worry scores than Group A, with a mean difference of –0.13 (*P*=.90). Group C also had nonsignificantly lower worry scores than Group A (mean difference –0.30; *P*=.77) and nonsignificantly higher scores than Group B (mean difference –0.17, *P*=.87). After participating in the SIMBIE process and adjusting for confounding factors, all 3 groups had negative trends in DSSQ-worry, though no significant differences were detected. Group B had nonsignificantly lower worry scores than Group A (mean difference 0.69; *P*=.45). Similarly, Group C had nonsignificantly lower worry scores than Group A (mean difference 0.97; *P*=.30) and nonsignificantly higher scores than Group B (mean difference 0.28; *P*=.77). See Figure 4, Table S3 in Multimedia Appendix 7, Table S4 in Multimedia Appendix 8, and Figure S1 in Multimedia Appendix 9.

### Measuring Burnout: Comparative Outcomes by Group

Our analysis of the CBI scores revealed discrepancies in results and interpretations between the ITT and PP analyses. In the ITT analysis comparing burnout outcomes between the pre-intervention assessment (phase 1) and the final assessment (phase 3), Group B had significantly lower burnout scores than Group A (mean difference –1.86, 95% CI 0.39 to 3.34; *P*=.01). Similarly, Group B had significantly lower burnout scores than Group C, with a mean difference of 1.59 (95% CI 0.11 to 3.06; *P*=.04). No statistically significant differences were observed between Group C and Group A, with a mean difference of –0.28 (95% CI –1.20 to 1.75; *P*=.07). See Figure 5, Table S3 in Multimedia Appendix 7, Table S4 in Multimedia Appendix 8, and Figure S2 in Multimedia Appendix 10.

In the PP analysis comparing burnout outcomes between the pre-intervention assessment (phase 1) and the final assessment (phase 3), Group B again had significantly lower burnout scores than Group A, with a mean difference of –2.02 (*P*=.02). However, the mean difference between Group B and Group C was not significant (mean difference 1.6; *P*=.07). Similarly, no statistically significant differences were observed between Group C and Group A, with a mean difference of –0.42 (*P*=.61). See Table S3 in Multimedia Appendix 7, Table S4 in Multimedia Appendix 8, and Figure S2 in Multimedia Appendix 10.

### Effect Sizes for Burnout Change Across the Multimodal Interventions

The ER-VIPE computer-based intervention (Group B) at 8 weeks demonstrated the largest reduction, with a small-to-moderate effect size (*d* =–0.31, 95% CI –0.78 to 0.15). Similarly, the combined medical movie and MOOC intervention (Groups B and C, pre-SIMBIE) at 2 weeks yielded a small-to-moderate effect (*d*=–0.30, 95% CI –0.63 to 0.03). In contrast, the computer-based SIMBIE with co-debriefing intervention (Group B) at 4 weeks showed a slight increase in burnout, with a small effect size (*d*=0.09, 95% CI –0.50 to 0.67). The ER-VIPE VR-based intervention (Group C) at 8 weeks was associated with an increase in burnout, with a moderate-to-large effect size (*d*=0.45, 95% CI –0.25 to 1.15). Notably, the VR-based SIMBIE with co-debriefing (Group C) at 4 weeks resulted in a substantial increase in burnout, demonstrating a large effect size (*d*=0.83, 95% CI 0.22 to 1.44), as shown in Table 2.

**Table 2:** Effect sizes for changes in burnout across multimodal interventions.

Group(s)	Intervention					Phase comparison		Duration (weeks)		Effect size, Cohen *d* (95% CI)^a^	
	Medical movie + MOOCs^b^	Computer-based SIMBIE^c^	VR^d^-based SIMBIE	Co-debriefing							
B^e^	Yes	Yes	No	Yes	1^f^ vs 3^g^		8		–0.31 (–0.75 to 0.15)		
B, C^h^	Yes	No	No	No	1 vs 2^i^		2		–0.30 (–0.63 to 0.03)		
A^j^	No	Yes	No	No	2 vs 3		6		–0.19 (–0.75 to 0.36)		
B	No	Yes	No	Yes	2 vs 3		6		0.09 (–0.50 to 0.67)		
C	Yes	No	Yes	Yes	1 vs 3		8		0.45 (–0.25 to 1.15)		
C	No	No	Yes	Yes	2 vs 3		6		0.83 (0.22 to 1.14)		

^a^Negative values indicate reductions in burnout: positive values indicate increases in burnout.

^b^MOOCs: massive open online courses.

^c^SIMBIE: simulation-based interprofessional education.

^d^VR: virtual reality.

^e^Group B received the movie + MOOCs, 3D computer-based SIMBIE, and co-debriefing.

^f^Phase 1: baseline; before movie + MOOCs.

^g^Phase 3: 8-week follow-up from baseline.

^h^Group C received the movie + MOOCs, VR-based SIMBIE, and co-debriefing.

^i^Phase 2: before SIMBIE; 2-week follow-up.

^j^Group A (control) received only 3D computer-based SIMBIE without debriefing.

## Discussion

### ER-VIPE: An Innovative Approach to Reducing Stress and Burnout

This study evaluated the effectiveness and quantified the effect sizes of multimodal educational strategies—medical movies, MOOCs, and computer- or VR-based SIMBIE with co-debriefing (collectively termed ER-VIPE)—for reducing burnout among future health care professionals. The ER-VIPE computer-based intervention (Group B) demonstrated the most notable reduction in burnout, with a small-to-moderate effect size (*d*=–0.31), followed closely by the combined medical movie and MOOC intervention after 2 weeks (*d*=–0.30).

In contrast, the computer-based SIMBIE with co-debriefing intervention (Group B) was associated with a minimal increase in burnout, reflected by a small effect size (*d*=0.09). The ER-VIPE VR-based intervention (Group C) showed a moderate-to-large increase in burnout (*d*=0.45), while the VR-based SIMBIE after 4 weeks resulted in the largest increase (*d*=0.83).

These findings suggest that the ER-VIPE computer-based multimodal approach, particularly when combined with co-debriefing, is more effective at mitigating burnout than the VR-based ER-VIPE intervention or single-component SIMBIE interventions. Additionally, medical movies and MOOCs alone contributed to a modest reduction in burnout after 2 weeks.

### Discussion: On the Meaningfulness of Effect Sizes in Burnout Prevention

The ER-VIPE computer-based intervention (Group B) demonstrated a small-to-moderate effect on burnout reduction, with an SMD of 0.31 (95% CI –0.15 to 0.78). Although the CI includes 0, the upper bound suggests a potentially meaningful impact, warranting further investigation in larger samples.

The ER-VIPE VR-based intervention (Group C) demonstrated a small-to-moderate effect for increasing burnout, with an SMD of 0.45 (95% CI –0.25 to 1.15). Although the CI includes 0, the upper bound suggests a potentially meaningful impact, warranting further investigation in larger samples.

Group B (ER-VIPE computer-based) demonstrated significantly lower burnout scores than Group A (computer-based SIMBIE without co-debriefing), with a mean difference of –1.86 (95% CI 0.39 to 3.34; *P*=.01). Although the beta coefficient (β=1.8, 95% CI 0.39 to 3.34) indicates a statistically significant effect, these findings should be interpreted with caution. Sole reliance on *P* values can be misleading, as statistical significance does not necessarily imply clinical or practical relevance. To address this, we calculated effect sizes (Cohen *d*) to better understand the magnitude of the observed effects. The ER-VIPE computer-based intervention yielded a small-to-medium effect size (*d*=–0.31), supporting its potential educational and clinical value.

From a practical standpoint, even small to modest effect sizes may have meaningful implications, especially in the context of early burnout prevention. The CBI (maximum 30 points), which was used in this study, is a culturally validated and widely adopted tool for assessing prolonged physical and psychological exhaustion. Previous studies have linked CBI scores to future risks such as absenteeism, sleep disturbances, increased use of painkillers, and intention to leave the profession [322]. In our study, a maximum reduction of approximately 3.34 points across 6 items (an average change of 0.56 per item on a 5-point Likert scale for 6 items) may reflect a meaningful shift in participants’ experiences—for example, from reporting they “always” feel overwhelmed to “seldom” feeling that they “can’t take it anymore.” Such changes, particularly in at-risk populations, could represent significant psychological relief.

Nonetheless, these findings should be interpreted cautiously. Self-reported burnout scores may be influenced by short-term emotional states or response-shift bias. Although the observed trend in burnout reduction is promising, especially for the ER-VIPE computer-based group, we consider the results preliminary and recommend further exploration through longitudinal research and real-world implementation studies.

### Effectiveness of Preparatory Tools and Instructional Design Considerations

Medical movies and MOOCs were used as preparatory tools prior to SIMBIE participation. Although these online modalities demonstrated some positive effects, their impact was variable and appeared to depend on factors such as learner readiness, the presence of an instructor [332], and the incorporation of structured guidance to direct learner attention toward key instructional scenes [333]. Consistent with our findings, the integration of cinemeducation and simulation has been demonstrated to be both feasible and effective, producing synergistic and convergent benefits from the perspectives of preclinical medical students. This combined approach also helps address some limitations of simulation alone in replicating complex professional scenarios, as supported by survey responses [334]. These findings underscore the critical role of intentional instructional design for optimizing the effectiveness of online educational interventions. Among all the interventions examined, the ER-VIPE computer-based model produced the most pronounced reduction in stress and burnout. This outcome may be attributed to its congruence with evidence-based learning principles; the integration of thoughtful SIMBIE design elements; and the use of accessible, user-friendly technology.

### Applying Experiential, Flipped, and SIT Frameworks in Learning Design

The learning design in this study was grounded in 3 complementary educational frameworks: Kolb’s experiential learning cycle, the flipped classroom model, and SIT. According to [335], learning is most effective when it progresses through 4 stages: concrete experience, reflective observation, abstract conceptualization, and active experimentation. To support this cycle, MOOCs were used to deliver theoretical foundations, while medical movies promoted critical thinking and contextual understanding by translating abstract concepts into realistic scenarios. Mayer’s cognitive theory of multimedia learning suggests that such video-based materials enhance knowledge retention by presenting information in a more concrete and engaging format [336]. Furthermore, analyzing film content through the lens of learned theories supports both cognitive and emotional development [337], while audiovisual elements foster realism and a more immersive learning experience [338-340].

Learners initially engaged in a SIMBIE session to establish a baseline experience, followed by co-debriefing guided by trained facilitators. This reflective process emphasized TeamSTEPPS concepts and aligned with Kolb’s stages of reflective observation and abstract conceptualization. Learners then applied newly acquired insights in a subsequent SIMBIE session, fulfilling the active experimentation phase.

To enhance preparedness, a flipped classroom approach was used. Presession exposure to medical movies and MOOCs helped build interprofessional knowledge and promoted positive attitudes toward IPE, TeamSTEPPS, and stress coping strategies [307]. This model improved learner engagement and emotional investment while maximizing the effectiveness of in-session SIMBIE activities [308]. Importantly, integrating stress management into real-world clinical education has been recognized as essential to professional education [309].

SIT further supported the design by providing a structured approach to building stress resilience. SIT involves 3 phases: conceptualization (understanding stress and personal reactions) via cinemeducation and MOOCs, skill acquisition (developing coping strategies such as communication) via SIMBIE, and skills consolidation reinforced through co-debriefing and application (practicing these skills in simulated or realistic scenarios as second round). This approach enabled learners to build psychological readiness for high-stress clinical environments. Similarly, Liaw et al [160] demonstrated that computer-based virtual SIMBIE could positively influence stress responses, measured via blood pressure and heart rate, and enhance team rescue performance, showing no significant difference compared with physical simulations. These findings were particularly valuable during the COVID-19 pandemic, as desktop VR offered a safe and effective alternative. Building on this, our study contributes new evidence that desktop-based SIMBIE within the ER-VIPE framework not only supports immediate stress management but also leads to sustained reductions in burnout observed at the 8-week follow-up.

In contrast, an RCT by Blanchard et al [268] found that a tutorial module on stress and stress management, followed by repeated non-IPE VR simulations under moderate to high stress conditions, was a feasible instructional approach. Participants in the intervention group reported lower perceived stress; reduced electrodermal activity, a physiological marker of stress measured through changes in the electrical conductivity of sweat on the hands or feet; and greater perceived competence after completing the test module compared with the control group. The training also appeared to facilitate desensitization to stress in future simulated scenarios. The differing outcomes between our study and the RCT by Blanchard et al [268] may be attributed to key methodological and contextual differences. Although the study by Blanchard et al used a controlled design with repeated VR exposure under moderate stress, induced by ecologically salient auditory stimuli, our quasi-experimental study featured more complex IPE scenarios involving a greater number of multiprofessional participants and team-based stress dynamics. These high-stakes, emotionally charged simulations may have exceeded the optimal arousal level described by the Yerkes-Dodson law [244], potentially leading to heightened stress.

Interestingly, a multicenter prospective randomized trial involving EM residents examined the effects of brief mental skills training delivered 1 month prior to simulation. The study reported no significant differences in subjective or objective stress responses, measured using heart rate variability and the STAI, during a non-IPE, manikin-based resuscitation simulation. This lack of measurable impact may be attributed to the extended gap between the conceptual (didactic) phase and the skill acquisition (simulation) phase, potentially hindering effective learning transfer. Moreover, the high-stress experiences of real-life clinical pressure may have reduced the simulation’s ability to elicit authentic stress responses. Notably, the study did not describe the debriefing process (consolidation phase) and did not include a second simulation for deliberate practice (application phase), which may have further limited the effectiveness of the SIT framework.

### SIMBIE Design Elements Supporting Safe, Realistic, and Collaborative Learning

The SIMBIE platform in this study was intentionally designed to replicate realistic clinical scenarios involving diverse health care students, with a focus on psychological safety, structured learning, and interprofessional collaboration. The learning sequence began with a prebriefing to outline objectives, facilitate ice-breaking activities, foster familiarity among participants, and establish a safe learning climate. A unique HP bar feature was incorporated to provide peer support during gameplay, and the session concluded with structured debriefing led by trained facilitators using a no-blame, psychologically safe approach—all conducted within a clearly defined time frame.

These design features align with evidence emphasizing that high-quality simulation-based education should include structured scenario progression, adaptability to learner actions, and the identification of critical performance points. Time-conscious design and high-fidelity environments further enhance learner engagement and realism. Equally important is the role of facilitators, who not only guide learning but also ensure that cultural diversity, psychological safety, and shared understanding of team roles are respected. Their guidance reinforces learning outcomes, encourages reflection, and supports the development of interprofessional competencies [341,342].

Findings from this study align with those of previous literature suggesting that interprofessional simulation-based approaches, when combined with skilled debriefing, enhance active engagement, self-efficacy, realistic role enactment, and collaborative team-based learning [340]. These experiences help students build essential interprofessional skills prior to clinical practice [343], gain insight into other professional roles [344,345], and foster deeper participation, particularly when stress is acknowledged and validated by peers or facilitators [346].

### Enhancing Accessibility and Reducing Stress Through User-Friendly Technology

The use of a computer-based platform in SIMBIE significantly enhanced accessibility and ease of use compared with VR headsets, which often require more complex operation. Consistent with previous studies, computer-based simulations have been shown to reduce negative emotional responses during learning [124,347]. Moreover, cine-VR training—delivered through head-mounted displays or 360-degree video—has been shown to enhance empathy among health care professional students, with no reported technological issues nor adverse effects [348]. In contrast, VR-based simulations have been associated with higher levels of distress due to technostress (eg, ergonomics, cybersickness, visual fatigue), or technological barriers, including user unfamiliarity, discomfort, and reluctance to adopt new tools [349-351]. Poorly designed VR experiences may further limit accessibility and user comfort [352,353]. In light of these challenges, computer-based simulation emerges as a more practical, scalable, and cost-effective educational approach [334], particularly in contexts aiming to reduce stress and promote learner engagement.

### Limitations

This study has several limitations. Conducted in a single university-based setting, the findings may have limited generalizability to other settings that differ in context and available facilities. This study was conducted during the middle of the COVID-19 pandemic, limiting its comparability to conventional clinical teaching, which is primarily conducted in clinical settings. The exclusion of participants with pre-existing mental health conditions may limit the generalizability of findings to broader populations. Although anxiety traits were controlled as a confounding factor, other potential influences on stress and burnout, such as individual stressors, baseline coping mechanisms, and levels of social support, were not assessed in this study and may have influenced participants’ responses. As a result, the findings should be interpreted with caution, as unmeasured psychosocial factors may have moderated participants’ responses to the simulation-based intervention. Although the DSSQ specifically measures stress during assigned tasks and the CBI captures broader aspects of exhaustion, these tools may not fully reflect the complexity of stress experiences in all contexts.

Additionally, dropout rates of 24%, 44%, and 14% in Groups A, B, and C, respectively, during the transition from phase 2 to phase 3 were addressed. We did not know the exact reasons for participant dropout. However, potential contributing factors may include exam schedules, personal or academic stress, mental health concerns, and minor technical issues related to the online survey platform sent via email. Additionally, the delayed delivery of monetary incentives—provided only after the second simulation, several weeks before the final assessment—may have reduced participants’ motivation to complete the study. We used GEEs to minimize sensitivity to missing data. An ITT analysis with imputed data was also performed to validate the PP findings. This indicates that, even though the dropout rate was low, the results were likely not significantly different from the original findings.

### Future Study

Future research should investigate the effectiveness of movies, MOOCs and virtual SIMBIE for reducing stress and burnout among diverse groups, such as postgraduate professionals and those in intensive care or prehospital settings. Future studies should consider including participants with mild or well-managed mental health comorbidities to enhance the generalizability and applicability of the intervention to more diverse, real-world clinical settings. We also recommend conducting an RCT comparing the intervention with traditional educational approaches in a nonpandemic context, such as in-person clinical teaching. To strengthen the rigor of future research, objective measures of stress, such as heart rate variability, electrodermal activity, or electroencephalography, should be incorporated. Additionally, potential confounding factors, including individual stressors, baseline coping mechanisms, and levels of social support, should be carefully assessed and controlled. A mixed methods approach is suggested to explore the mechanisms of impact and identify areas for improvement. Furthermore, longitudinal studies using multiple stress and burnout assessment methods are necessary to comprehensively evaluate long-term outcomes.

### Conclusion

This study highlights the effectiveness of a novel multimodal learning approach that integrates movie-based education, MOOCs, and a virtual 3D computer-based SIMBIE with co-debriefing for reducing burnout and improving self-reported stress levels by enhancing engagement and reducing worry and distress among undergraduate clinical students. These innovative IPE strategies are designed to help multiprofessional students manage stress, reduce burnout, and develop collaborative problem-solving skills within authentic simulation environments. By alleviating the pressures and risks typically associated with clinical practice, the interactive and scalable 3D computer-based ER-VIPE platform supports 21st-century health care learners, where patient safety is paramount. Integrating these tools and strategies into IPE programs offers significant potential to enhance well-being and resilience while preparing health care students and early-career professionals for a smooth transition into clinical practice.

## Appendix

Multimedia Appendix 1 Electroencephalogram (EEG) Procedure.

Multimedia Appendix 2 CHERRIES (Checklist for Reporting Results of Internet E-Surveys).

Multimedia Appendix 3 CONSORT-eHEALTH checklist (V 1.6.1).

Multimedia Appendix 4 Measurement instruments for state stress, burnout, and anxiety trait.

Multimedia Appendix 5 Baseline burnout and Dundee Stress State Questionnaire (DSSQ) scores.

Multimedia Appendix 6 Time interval assessments.

Multimedia Appendix 7 Improvement in burnout and Dundee Stress State Questionnaire (DSSQ) scores: intention-to-treat analysis.

Multimedia Appendix 8 Per-protocol analysis for burnout and Dundee Stress State Questionnaire (DSSQ) improvements.

Multimedia Appendix 9 Dundee Stress State Questionnaire (DSSQ) changes based on per-protocol analysis.

Multimedia Appendix 10 Copenhagen Burnout Inventory (CBI) changes based on per-protocol analysis.

## Abbreviations

CBI: Copenhagen Burnout Inventory
CHERRIES: Checklist for Reporting Results of Internet E-Surveys
cMOOC: connectivist massive open online course
CONSORT-EHEALTH: Consolidated Standards of Reporting Trials of Electronic and Mobile Health Applications and Online Telehealth
DSSQ: Dundee Stress State Questionnaire
EM: emergency medicine
ER-VIPE: Emergency Room Virtual Simulation Interprofessional Education
GEE: generalized estimating equation
HCW: health care worker
HP: health point
iMOOC: integrated massive open online course
IPC: interprofessional collaboration
IPE: interprofessional education
ISBAR: Identify, Situation, Background, Assessment, and Recommendation
ITT: intention-to-treat
JD-R: Job Demands-Resources
MANOVA: multivariate analysis of variance
MOOC: massive open online course
PEARLS: Promoting Excellence and Reflective Learning in Simulation
PHQ-9: Patient Health Questionnaire-9
PP: per-protocol
PPE: personal protective equipment
RCT: randomized controlled trial
SIMBIE: simulation-based interprofessional education
SIT: stress inoculation training
SMD: standardized mean difference
STAI: State-Trait Anxiety Inventory
TeamSTEPPS: Team Strategies and Tools to Enhance Performance and Patient Safety
VR: virtual reality
WHO: World Health Organization
xMOOC: massive open online course that is an extension of something else
