# Supplementary material for: Comparing the Effectiveness of Multimodal Learning Using Computer-Based and Immersive Virtual Reality Simulation–Based Interprofessional Education With Co-Debriefing, Medical Movies, and Massive Online Open Courses for Mitigating Stress and Long-Term Burnout in Medical Training: Quasi-Experimental Study
Source: JMIR Med Educ. 2025 Sep 24;11:e70726. doi: 10.2196/70726 (PMC12508677; doi:10.2196/70726)
Supplement: Multimedia Appendix 1 [file mededu_v11i1e70726_app1.docx]

**Multimedia Appendix 8: Electroencephalogram (EEG) Procedure**

### **Electroencephalogram (EEG) Procedure as a Potential Confounding Factor**

During **Phase 1**, participants completed the pretest DSSQ, CBI, and STAI assessments before being fitted with a 64-electrode EEG cap using the Biosemi Active-Two amplifier system (Amsterdam, the Netherlands). The EEG cap installation process took approximately 60 minutes. Following installation, participants completed a 5-minute eye-closed resting state. Participants in Group A then had their EEG caps removed, while those in Groups B and C continued wearing the caps during the medical movie and MOOC sessions, which lasted 180 minutes. After these activities, participants completed the posttest DSSQ, and the EEG caps were removed.

During **Phase 2**, participants completed the pretest DSSQ and CBI assessments before being fitted with a 15-electrode EEG cap using the Open BCI EEG system (New York, United States). Installation took approximately 30 minutes. Participants wore the caps while completing the 30-minute SIMBIE simulation, with or without the 60-minute co-debriefing. The caps were removed after participants completed the posttest DSSQ.

Although the EEG procedure was primarily conducted as part of a separate study measuring team performance via EEG, it is described here due to its potential role as a confounding factor that may have influenced stress levels during the interventions.
