# Supplementary material for: Comparing the Effectiveness of Multimodal Learning Using Computer-Based and Immersive Virtual Reality Simulation–Based Interprofessional Education With Co-Debriefing, Medical Movies, and Massive Online Open Courses for Mitigating Stress and Long-Term Burnout in Medical Training: Quasi-Experimental Study
Source: JMIR Med Educ. 2025 Sep 24;11:e70726. doi: 10.2196/70726 (PMC12508677; doi:10.2196/70726)
Supplement: Multimedia Appendix 2 [file mededu_v11i1e70726_app2.docx]

## Comparing the Effectiveness of Multimodal Learning Using Computer-Based and Immersive Virtual Reality Simulation-Based Interprofessional Education with Co-Debriefing, Medical Movies, MOOCs in Mitigating Stress and Long-Term Burnout in Medical Training: A Quasi-Experimental Study

| 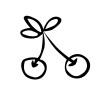 | **Checklist for Reporting Results of Internet E-Surveys (CHERRIES)** | |
| --- | --- | --- |
| ***Item Category*** | ***Checklist Item*** | ***Explanation*** |
| **Design** |  |  |
|  | A quasi-experimental study | A quasi-experimental study with a single-blinded statistician was conducted to compare stress and burnout levels among healthcare professional students, who were divided into 3 groups: two received novel educational interventions, and one served as the control group with traditional learning methods. The study was conducted at a 1500-bed university-affiliated hospital in Bangkok, Thailand, which served as a training site for multiple healthcare professional programs. A quasi-experimental design was used due to the COVID-19 pandemic practical challenges of randomization in a clinical setting. |
| **IRB (Institutional Review Board) approval and informed consent process** |  |  |
|  | IRB approval | The study protocol was approved by the Ethics Committee for Research in Human Subjects of the Faculty of Medicine, Chulalongkorn University, Thailand (IRB No. 0366/65).. |
|  | Informed consent | Participants were informed about the study's objectives, procedures, potential risks, and benefits. This information was provided both orally and in writing before obtaining informed consent. Participants were assured of their right to make voluntary decisions and withdraw from the study at any time. |
|  | Data protection | To protect the confidentiality of individual participants, all personal identifiers were removed and the data were fully anonymized. The dataset was securely stored on Google Drive with access granted solely to members of the research team. Strict access controls were implemented to prevent unauthorized individuals from accessing the data. |
| **Development and pre-testing** |  |  |
|  | Development and testing | Survey Development and InstrumentsThe Qualtrics-based survey was developed by a trained research assistant under the supervision of the research team. Prior to deployment, it was pilot-tested to assess usability and technical performance. Revisions were made based on feedback to improve clarity and system stability. The details of our survey are as follows:Burnout MeasurementBurnout was measured using the 6-item personal burnout subscale from the Copenhagen Burnout Inventory (CBI), which assesses physical and psychological exhaustion. Responses were recorded on a 5-point Likert scale (1 = never/almost never, 5 = always).State Stress MeasurementState stress was assessed using the 24-item short version of the Dundee Stress State Questionnaire (DSSQ), covering task engagement, distress, and worry. Items were rated on a 5-point Likert scale (1 = not at all, 5 = extremely). An example item is: “I was motivated to do the task.” The DSSQ, used with permission from Prof. Helton, is a validated and reliable tool (Cronbach’s α > 0.80). It was translated into Thai via back-translation by J. Chavanovanich, PhD (email, August 21, 2024). |
| **Recruitment process and description of the sample having access to the questionnaire** |  |  |
|  | Open survey versus closed survey | The closed survey targeted only the 90 participants who met the predefined inclusion and exclusion criteria. |
|  | Contact mode | After approval from the IRB committee, to facilitate participant recruitment, potential participants—including students of medicine, nursing, pharmacy, radiology, and medical technology—were contacted through multiple channels. These included verbal communication by the research team in coordination with representatives from each department, Line application, and printed posters. |
|  | Advertising the survey | The survey enrollment was promoted using posters and electronic communications containing a QR code that linked to a Google Form. The principal investigator’s name and telephone number were provided to facilitate further inquiries. The enrollment process was designed to avoid disruption to participants’ regular class schedules |
| **Survey administration** |  |  |
|  | Web/E-mail | The survey was administered electronically via a Qualtrics-based web link, with responses automatically captured and stored within the Qualtrics system. During the follow-up phase, the survey link was distributed via e-mail. |
|  | Context | The survey was not posted on a public website but distributed via internal academic channels, including LINE groups, and posters commonly used by students in medicine, nursing, pharmacy, radiological technology, and medical technology. These platforms share academic and research-related information. The survey link was accessed through a QR code directing to a Google Form. Since recruitment was limited to students within clinical programs at our institution, the distribution method is unlikely to pre-select the sample based on external interests or introduce topic-related bias. |
|  | Mandatory/voluntary | The survey was completely voluntary. It was not mandatory for visitors to access any website, nor was it required for any academic purpose. Participation was open to eligible students who chose to enroll via a QR code linked to a Google Form, shared through institutional communication channels such as LINE, and posters. No academic activities or website access were contingent upon survey completion. |
|  | Incentives | Yes, monetary incentives were offered. Participants received 500 Baht per session. This compensation was provided to acknowledge their time and contribution. No other prizes or non-monetary incentives (such as access to survey results) were offered. Participation was entirely voluntary and did not interfere with students’ regular academic schedules. |
|  | Time/Date | Data were collected between July 2022 and September 2023 |
|  | Randomization of items or questionnaires | Items were not fully randomized; however, to reduce response bias, the direction of some Likert-scale items in the questionairs was alternated (i.e., reverse-coded) to encourage thoughtful responses and minimize acquiescence bias. |
|  | Adaptive questioning | Adaptive DSSQ and CBI questioning was applied by shortening and simplifying some items, including the incorporation of negative items, to reduce the number and complexity of questions. |
|  | Number of Items | The DSSQ is a 24-item multidimensional measure comprising task engagement, distress, and worry. A 6-item personal burnout subscale from the CBI was used to assess personal burnout. |
|  | Number of screens (pages) | Three screens (pages) were used for the pre- and post-survey phases, and two screens (pages) were used for the follow-up phase. |
|  | Completeness check | No consistency or completeness checks (e.g., using JavaScript) were implemented before submission of the questionnaire. Additionally, the system did not perform post-submission completeness checks or highlight missing mandatory fields. The survey did not include explicit non-response options such as “not applicable” or “prefer not to say,” and there was no forced-response setting applied to any items. |
|  | Review step | Respondents were able to review and modify their answers prior to submission using the "Back" button. However, the survey did not include a final review step that summarized all responses for confirmation before submission. Once the questionnaire was submitted, respondents were no longer able to revise their answers or resubmit the form. |
| **Response rates** |  |  |
|  | Unique site visitor | View rates and participation rates were not calculated or reported in this study. |
|  | View rate (Ratio of unique survey visitors/unique site visitors) | View rates and participation rates were not calculated or reported in this study. |
|  | Participation rate (Ratio of unique visitors who agreed to participate/unique first survey page visitors) | Participation Rate:  The participation rate was calculated based on the number of unique individuals who registered to participate in the study (147 students) divided by the estimated number of unique visitors to the first survey page, which is approximated by the total eligible population of 533 undergraduate clinical students across five healthcare professions.  Participation rate = 147 / 533 ≈ 27.6%  This indicates a voluntary participation rate of approximately 28% among the target population. |
|  | Completion rate (Ratio of users who finished the survey/users who agreed to participate) | Out of 147 students who agreed to participate, a total of 63 participants (22 from Group A, 16 from Group B, and 25 from Group C) completed the full study protocol.  Completion rate = 63 / 147 ≈ 42.9% |
| **Preventing multiple entries from the same individual** |  |  |
|  | Cookies used | No cookies were used to assign a unique user identifier to each client computer. Duplicate entries were avoided by configuring the online survey platform to prevent users from accessing and submitting the questionnaire more than once. |
|  | IP check | The IP address of the client computer was not used to identify or restrict duplicate entries. |
|  | Log file analysis | No additional techniques were used to analyze the log file for identification of multiple entries. |
|  | Registration | In this closed survey, users were identified through their login credentials, and the survey system was designed to allow only one submission per user. If duplicate entries occurred, user identifiers (e.g., email or username) were used to detect and eliminate redundant responses. In such cases, only the first entry was retained for analysis, and subsequent submissions were excluded to ensure data integrity. |
| **Analysis** |  |  |
|  | Handling of incomplete questionnaires | Only fully completed questionnaires were included in the analysis. The survey system was designed such that participants were required to answer all questions before submission; incomplete questionnaires could not be submitted. |
|  | Questionnaires submitted with an atypical timestamp | No time-based cut-off point was applied. All submitted questionnaires were included in the analysis regardless of completion time, as response duration was not used as an exclusion criterion in this study. |
|  | Statistical correction | **Statistical Correction:**  No weighting of items or use of propensity scores was applied in this study to adjust for non-representative sampling. All analyses were conducted on the observed data without applying sampling weights. |
