# Supplementary material for: Comparing the Effectiveness of Multimodal Learning Using Computer-Based and Immersive Virtual Reality Simulation–Based Interprofessional Education With Co-Debriefing, Medical Movies, and Massive Online Open Courses for Mitigating Stress and Long-Term Burnout in Medical Training: Quasi-Experimental Study
Source: JMIR Med Educ. 2025 Sep 24;11:e70726. doi: 10.2196/70726 (PMC12508677; doi:10.2196/70726)
Supplement: Multimedia Appendix 3 [file mededu_v11i1e70726_app3.pdf]

CONSORT-EHEALTH (V 1.6.1) - Submission/Publication Form

The CONSORT-EHEALTH checklist is intended for authors of randomized trials evaluating web-based and Internet-based applications/interventions, including mobile interventions, electronic games (incl multiplayer games), social media, certain telehealth applications, and other interactive and/or networked electronic applications. Some of the items (e.g. all subitems under item 5 - description of the intervention) may also be applicable for other study designs.

The goal of the CONSORT EHEALTH checklist and guideline is to be
a) a guide for reporting for authors of RCTs,
b) to form a basis for appraisal of an ehealth trial (in terms of validity)

CONSORT-EHEALTH items/subitems are MANDATORY reporting items for studies published in the Journal of Medical Internet Research and other journals / scientific societies endorsing the checklist.

Items numbered 1., 2., 3., 4a., 4b etc are original CONSORT or CONSORT-NPT (non-pharmacologic treatment) items.
Items with Roman numerals (i., ii, iii, iv etc.) are CONSORT-EHEALTH extensions/clarifications.

As the CONSORT-EHEALTH checklist is still considered in a formative stage, we would ask that you also RATE ON A SCALE OF 1-5 how important/useful you feel each item is FOR THE PURPOSE OF THE CHECKLIST and reporting guideline (optional).

Mandatory reporting items are marked with a red *.
In the textboxes, either copy & paste the relevant sections from your manuscript into this form - please include any quotes from your manuscript in QUOTATION MARKS,
or answer directly by providing additional information not in the manuscript, or elaborating on why the item was not relevant for this study.

YOUR ANSWERS WILL BE PUBLISHED AS A SUPPLEMENTARY FILE TO YOUR PUBLICATION IN JMIR AND ARE CONSIDERED PART OF YOUR PUBLICATION (IF ACCEPTED).
Please fill in these questions diligently. Information will not be copyedited, so please use proper spelling and grammar, use correct capitalization, and avoid abbreviations.

DO NOT FORGET TO SAVE AS PDF _AND_ CLICK THE SUBMIT BUTTON SO YOUR ANSWERS ARE IN OUR DATABASE !!!

Citation Suggestion (if you append the pdf as Appendix we suggest to cite this paper in the caption):
Eysenbach G, CONSORT-EHEALTH Group
CONSORT-EHEALTH: Improving and Standardizing Evaluation Reports of Web-based and Mobile Health Interventions
J Med Internet Res 2011;13(4):e126
URL: http://www.jmir.org/2011/4/e126/
doi: 10.2196/jmir.1923
PMID: 22209829

khuansiri.n@chula.ac.th สลับบัญชี

ไม่ใช้ร่วมกัน

บันทึกฉบับร่างแล้ว

* ระบุว่าเป็นคำถามที่จำเป็น

Your name *

First Last

Khuansiri Narajeenron

Primary Affiliation (short), City, Country *

University of Toronto, Toronto, Canada

Department of Emergency, Medicine, Faculty o

Your e-mail address *

abc@gmail.com

khuansiri.n@chula.ac.th

Title of your manuscript *

Provide the (draft) title of your manuscript.

Comparing the Effectiveness of Multimodal Learning Using Computer-Based and Immersive Virtual Reality Simulation-Based Interprofessional Education with Co-Debriefing, Medical Movies, MOOCs in Mitigating Stress and Long-Term Burnout in Medical Training: A Quasi-Experimental Study

Name of your App/Software/Intervention *

If there is a short and a long/alternate name, write the short name first and add the long name in brackets.

ER-VIPE (Emergency Room Virtual Interprofess

Evaluated Version (if any)

e.g. "V1", "Release 2017-03-01", "Version 2.0.27913"

Version 0.3.14b_ EEG

Language(s) *

What language is the intervention/app in? If multiple languages are available, separate by comma (e.g. "English, French")

English

URL of your Intervention Website or App

e.g. a direct link to the mobile app on app in appstore (itunes, Google Play), or URL of the website. If the intervention is a DVD or hardware, you can also link to an Amazon page.

www.ervipe.com

URL of an image/screenshot (optional)

คำตอบของคุณ

Accessibility *

Can an enduser access the intervention presently?

- ○ access is free and open
- ◉ access only for special usergroups, not open
- ○ access is open to everyone, but requires payment/subscription/in-app purchases
- ○ app/intervention no longer accessible
- ○ อื่นๆ:

Primary Medical Indication/Disease/Condition *

e.g. "Stress", "Diabetes", or define the target group in brackets after the condition, e.g. "Autism (Parents of children with)", "Alzheimers (Informal Caregivers of)"

Stress in clinical healthcare students

Primary Outcomes measured in trial *

comma-separated list of primary outcomes reported in the trial

This study aimed to evaluate the effectiveness

Secondary/other outcomes

Are there any other outcomes the intervention is expected to affect?

No other outcomes were anticipated or assessed beyond the primary measures

Recommended "Dose" *

What do the instructions for users say on how often the app should be used?

- ○ Approximately Daily
- ○ Approximately Weekly
- ◉ Approximately Monthly
- ○ Approximately Yearly
- ○ "as needed"
- ○ อื่นๆ:

?

Approx. Percentage of Users (starters) still using the app as recommended after 3 months *

- [x] unknown / not evaluated
- [ ] 0-10%
- [ ] 11-20%
- [ ] 21-30%
- [ ] 31-40%
- [ ] 41-50%
- [ ] 51-60%
- [ ] 61-70%
- [ ] 71%-80%
- [ ] 81-90%
- [ ] 91-100%
- [ ] อื่นๆ:

Overall, was the app/intervention effective? *

- (selected) yes: all primary outcomes were significantly better in intervention group vs control
- partly: SOME primary outcomes were significantly better in intervention group vs control
- no statistically significant difference between control and intervention
- potentially harmful: control was significantly better than intervention in one or more outcomes
- inconclusive: more research is needed
- อื่นๆ:

Article Preparation Status/Stage *

At which stage in your article preparation are you currently (at the time you fill in this form)

- ( ) not submitted yet - in early draft status
- ( ) not submitted yet - in late draft status, just before submission
- ( ) submitted to a journal but not reviewed yet
- (•) submitted to a journal and after receiving initial reviewer comments
- ( ) submitted to a journal and accepted, but not published yet
- ( ) published
- ( ) อื่นๆ:

Journal *

If you already know where you will submit this paper (or if it is already submitted), please provide the journal name (if it is not JMIR, provide the journal name under "other")

- ◯ not submitted yet / unclear where I will submit this
- ◯ Journal of Medical Internet Research (JMIR)
- ◯ JMIR mHealth and UHealth
- ◯ JMIR Serious Games
- ◯ JMIR Mental Health
- ◯ JMIR Public Health
- ◯ JMIR Formative Research
- ◉ Other JMIR sister journal
- ◯ อื่นๆ:

Is this a full powered effectiveness trial or a pilot/feasibility trial? *

- ◯ Pilot/feasibility
- ◉ Fully powered

Manuscript tracking number *

If this is a JMIR submission, please provide the manuscript tracking number under "other" (The ms tracking number can be found in the submission acknowledgement email, or when you login as author in JMIR. If the paper is already published in JMIR, then the ms tracking number is the four-digit number at the end of the DOI, to be found at the bottom of each published article in JMIR)

- ○ no ms number (yet) / not (yet) submitted to / published in JMIR
- ◉ อื่นๆ: ID 70726

TITLE AND ABSTRACT

1a) TITLE: Identification as a randomized trial in the title

1a) Does your paper address CONSORT item 1a? *

I.e does the title contain the phrase "Randomized Controlled Trial"? (if not, explain the reason under "other")

- ○ yes
- ◉ อื่นๆ: A Quasi-Experimental Study

1a-i) Identify the mode of delivery in the title

Identify the mode of delivery. Preferably use “web-based” and/or “mobile” and/or “electronic game” in the title. Avoid ambiguous terms like “online”, “virtual”, “interactive”. Use “Internet-based” only if Intervention includes non-web-based Internet components (e.g. email), use “computer-based” or “electronic” only if offline products are used. Use “virtual” only in the context of “virtual reality” (3-D worlds). Use “online” only in the context of “online support groups”. Complement or substitute product names with broader terms for the class of products (such as “mobile” or “smart phone” instead of “iphone”), especially if the application runs on different platforms.

	1	2	3	4	5	
subitem not at all important	○	○	○	○	◉	essential

ล้างสิ่งที่เลือก

Does your paper address subitem 1a-i? *

Copy and paste relevant sections from manuscript title (include quotes in quotation marks "like this" to indicate direct quotes from your manuscript), or elaborate on this item by providing additional information not in the ms, or briefly explain why the item is not applicable/relevant for your study

"Comparing the Effectiveness of Multimodal Learning Using Computer-Based and Immersive Virtual Reality Simulation-Based Interprofessional Education with Co-Debriefing, Medical Movies, MOOCs in Mitigating Stress and Long-Term Burnout in Medical Training: A Quasi-Experimental Study"

1a-ii) Non-web-based components or important co-interventions in title

Mention non-web-based components or important co-interventions in title, if any (e.g., “with telephone support”).

	1	2	3	4	5	
subitem not at all important	○	○	○	○	◉	essential

ล้างสิ่งที่เลือก

Does your paper address subitem 1a-ii?

Copy and paste relevant sections from manuscript title (include quotes in quotation marks "like this" to indicate direct quotes from your manuscript), or elaborate on this item by providing additional information not in the ms, or briefly explain why the item is not applicable/relevant for your study

"Comparing the Effectiveness of Multimodal Learning Using Computer-Based and Immersive Virtual Reality Simulation-Based Interprofessional Education with Co-Debriefing, Medical Movies, MOOCs in Mitigating Stress and Long-Term Burnout in Medical Training: A Quasi-Experimental Study"

1a-iii) Primary condition or target group in the title

Mention primary condition or target group in the title, if any (e.g., “for children with Type I Diabetes”) Example: A Web-based and Mobile Intervention with Telephone Support for Children with Type I Diabetes: Randomized Controlled Trial

	1	2	3	4	5	
subitem not at all important	○	○	○	○	◉	essential

ล้างสิ่งที่เลือก

Does your paper address subitem 1a-iii? *

Copy and paste relevant sections from manuscript title (include quotes in quotation marks "like this" to indicate direct quotes from your manuscript), or elaborate on this item by providing additional information not in the ms, or briefly explain why the item is not applicable/relevant for your study

"Comparing the Effectiveness of Multimodal Learning Using Computer-Based and Immersive Virtual Reality Simulation-Based Interprofessional Education with Co-Debriefing, Medical Movies, MOOCs in Mitigating Stress and Long-Term Burnout in Medical Training: A Quasi-Experimental Study"

1b) ABSTRACT: Structured summary of trial design, methods, results, and conclusions

NPT extension: Description of experimental treatment, comparator, care providers, centers, and blinding status.

1b-i) Key features/functionalities/components of the intervention and comparator in the METHODS section of the ABSTRACT

Mention key features/functionalities/components of the intervention and comparator in the abstract. If possible, also mention theories and principles used for designing the site. Keep in mind the needs of systematic reviewers and indexers by including important synonyms. (Note: Only report in the abstract what the main paper is reporting. If this information is missing from the main body of text, consider adding it)

	1	2	3	4	5	
subitem not at all important	○	○	○	○	◉	essential

ล้างสิ่งที่เลือก

?

Does your paper address subitem 1b-i? *

Copy and paste relevant sections from the manuscript abstract (include quotes in quotation marks "like this" to indicate direct quotes from your manuscript), or elaborate on this item by providing additional information not in the ms, or briefly explain why the item is not applicable/relevant for your study

"Methods: A single-blinded quasi-experimental study was conducted at a university hospital from July 2022 to September 2023. A three-Group treatment design, Group A (control) participated in a 3D computer-based simulation-based interprofessional education (SIMBIE) without debriefing. Group B received a medical movie, MOOCs, a 3D computer-based SIMBIE, collectively referred to as ER-VIPE (Emergency Room Virtual Interprofessional Education). and an co-debriefing session. Group C received the same interventions as Group B, except the 3D computer-based SIMBIE was replaced with a 3D virtual reality SIMBIE. SIMBIE experiential learning activities involving a COVID-19 pneumonia crisis scenario. Outcome measures included the Dundee Stress State Questionnaire (DSSQ) and Copenhagen Burnout Inventory. Anxiety trait scores served as behavioral control. Self-reported stress and burnout levels were measured at baseline, pre-intervention, post-intervention, and one-month post-intervention. Generalized Estimating Equations (GEE) was employed to analyze variations in outcomes among the three groups. Statistical significance was set at $P<.05$."

1b-ii) Level of human involvement in the METHODS section of the ABSTRACT

Clarify the level of human involvement in the abstract, e.g., use phrases like “fully automated” vs. “therapist/nurse/care provider/physician-assisted” (mention number and expertise of providers involved, if any). (Note: Only report in the abstract what the main paper is reporting. If this information is missing from the main body of text, consider adding it)

	1	2	3	4	5	
subitem not at all important	○	○	○	○	◉	essential

ล้างสิ่งที่เลือก

Does your paper address subitem 1b-ii?

Copy and paste relevant sections from the manuscript abstract (include quotes in quotation marks "like this" to indicate direct quotes from your manuscript), or elaborate on this item by providing additional information not in the ms, or briefly explain why the item is not applicable/relevant for your study

"Methods: A single-blinded quasi-experimental study was conducted at a university hospital from July 2022 to September 2023. A three-Group treatment design, Group A (control) participated in a 3D computer-based simulation-based interprofessional education (SIMBIE) without debriefing. Group B received a medical movie, MOOCs, a 3D computer-based SIMBIE, collectively referred to as ER-VIPE (Emergency Room Virtual Interprofessional Education). and an co-debriefing session. Group C received the same interventions as Group B, except the 3D computer-based SIMBIE was replaced with a 3D virtual reality SIMBIE. SIMBIE experiential learning activities involving a COVID-19 pneumonia crisis scenario. Outcome measures included the Dundee Stress State Questionnaire (DSSQ) and Copenhagen Burnout Inventory. Anxiety trait scores served as behavioral control. Self-reported stress and burnout levels were measured at baseline, pre-intervention, post-intervention, and one-month post-intervention. Generalized Estimating Equations (GEE) was employed to analyze variations in outcomes among the three groups. Statistical significance was set at P<.05."

1b-iii) Open vs. closed, web-based (self-assessment) vs. face-to-face assessments in the METHODS section of the ABSTRACT

Mention how participants were recruited (online vs. offline), e.g., from an open access website or from a clinic or a closed online user group (closed usergroup trial), and clarify if this was a purely web-based trial, or there were face-to-face components (as part of the intervention or for assessment). Clearly say if outcomes were self-assessed through questionnaires (as common in web-based trials). Note: In traditional offline trials, an open trial (open-label trial) is a type of clinical trial in which both the researchers and participants know which treatment is being administered. To avoid confusion, use “blinded” or “unblinded” to indicated the level of blinding instead of “open”, as “open” in web-based trials usually refers to “open access” (i.e. participants can self-enrol). (Note: Only report in the abstract what the main paper is reporting. If this information is missing from the main body of text, consider adding it)

	1	2	3	4	5	
subitem not at all important	○	○	○	○	◉	essential

ล้างสิ่งที่เลือก

Does your paper address subitem 1b-iii?

Copy and paste relevant sections from the manuscript abstract (include quotes in quotation marks "like this" to indicate direct quotes from your manuscript), or elaborate on this item by providing additional information not in the ms, or briefly explain why the item is not applicable/relevant for your study

"Methods: A single-blinded quasi-experimental study was conducted at a university hospital from July 2022 to September 2023. A three-Group treatment design, Group A (control) participated in a 3D computer-based simulation-based interprofessional education (SIMBIE) without debriefing. Group B received a medical movie, MOOCs, a 3D computer-based SIMBIE, collectively referred to as ER-VIPE (Emergency Room Virtual Interprofessional Education). and an co-debriefing session. Group C received the same interventions as Group B, except the 3D computer-based SIMBIE was replaced with a 3D virtual reality SIMBIE. SIMBIE experiential learning activities involving a COVID-19 pneumonia crisis scenario. Outcome measures included the Dundee Stress State Questionnaire (DSSQ) and Copenhagen Burnout Inventory. Anxiety trait scores served as behavioral control. Self-reported stress and burnout levels were measured at baseline, pre-intervention, post-intervention, and one-month post-intervention. Generalized Estimating Equations (GEE) was employed to analyze variations in outcomes among the three groups. Statistical significance was set at P<.05."

1b-iv) RESULTS section in abstract must contain use data

Report number of participants enrolled/assessed in each group, the use/uptake of the intervention (e.g., attrition/adherence metrics, use over time, number of logins etc.), in addition to primary/secondary outcomes. (Note: Only report in the abstract what the main paper is reporting. If this information is missing from the main body of text, consider adding it)

	1	2	3	4	5	
subitem not at all important	○	○	○	○	◉	essential

ล้างสิ่งที่เลือก

Does your paper address subitem 1b-iv?

Copy and paste relevant sections from the manuscript abstract (include quotes in quotation marks "like this" to indicate direct quotes from your manuscript), or elaborate on this item by providing additional information not in the ms, or briefly explain why the item is not applicable/relevant for your study

"Results: Eighty-seven clinical undergraduate students from various professional programs participated in the study, with 29 students in each group (A, B, and C). Groups showed no significant differences in age, gender, or academic standing, with an average age of 21.87 years, and 71% female. Most nursing, radiological technology, and medical technology students were in their fourth year, while medical and pharmacy students were in their fifth and sixth years, respectively. After a 1-month post-SIMBIE follow-up, adjusted analyses revealed positive trends in DSSQ-engagement across all groups, with Group B showing a significant increase compared to Group A (mean difference=3.93; P=.001). DSSQ-worry and DSSQ-distress scores decreased non-significantly across all groups. Burnout scores also improved across groups, with Group B showing a significantly lower score than Group A (mean difference=−2.02; P=.02). No significant burnout differences were found between Group C and Groups A or B."

1b-v) CONCLUSIONS/DISCUSSION in abstract for negative trials

Conclusions/Discussions in abstract for negative trials: Discuss the primary outcome - if the trial is negative (primary outcome not changed), and the intervention was not used, discuss whether negative results are attributable to lack of uptake and discuss reasons. (Note: Only report in the abstract what the main paper is reporting. If this information is missing from the main body of text, consider adding it)

	1	2	3	4	5	
subitem not at all important	(●)	○	○	○	○	essential

ล้างสิ่งที่เลือก

Does your paper address subitem 1b-v?

Copy and paste relevant sections from the manuscript abstract (include quotes in quotation marks "like this" to indicate direct quotes from your manuscript), or elaborate on this item by providing additional information not in the ms, or briefly explain why the item is not applicable/relevant for your study

This subitem is not applicable, as it was not relevant to the scope of our study

INTRODUCTION

2a) In INTRODUCTION: Scientific background and explanation of rationale

2a-i) Problem and the type of system/solution

Describe the problem and the type of system/solution that is object of the study: intended as stand-alone intervention vs. incorporated in broader health care program? Intended for a particular patient population? Goals of the intervention, e.g., being more cost-effective to other interventions, replace or complement other solutions? (Note: Details about the intervention are provided in “Methods” under 5)

	1	2	3	4	5	
subitem not at all important	○	○	○	○	◉	essential

ล้างสิ่งที่เลือก

Does your paper address subitem 2a-i? *

Copy and paste relevant sections from the manuscript (include quotes in quotation marks "like this" to indicate direct quotes from your manuscript), or elaborate on this item by providing additional information not in the ms, or briefly explain why the item is not applicable/relevant for your study

"Background: Burnout among emergency room healthcare workers (HCWs) has reached critical levels, with up to 43% of all HCWs and 35% of emergency medicine personnel affected during the COVID-19 pandemic. Nurses were the most impacted, followed by physicians, leading to absenteeism, diminished care quality, and high turnover rates—reaching 78% in some settings, such as Thailand. Beyond workforce instability, burnout significantly compromises patient safety. Each one-unit increase in emotional exhaustion has been associated with a 2.63-fold increase in reports of poor care quality, a 30% rise in patient falls, a 47% increase in medication errors, and a 32% increase in healthcare-associated infections. Burnout is also linked to declining job satisfaction, worsening mental health, and increased intent to leave the profession. These findings highlight the urgent need for effective strategies to reduce stress and burnout in emergency care settings."

2a-ii) Scientific background, rationale: What is known about the (type of) system

Scientific background, rationale: What is known about the (type of) system that is the object of the study (be sure to discuss the use of similar systems for other conditions/diagnoses, if appropiate), motivation for the study, i.e. what are the reasons for and what is the context for this specific study, from which stakeholder viewpoint is the study performed, potential impact of findings [2]. Briefly justify the choice of the comparator.

	1	2	3	4	5	
subitem not at all important	○	○	○	○	◉	essential

ล้างสิ่งที่เลือก

Does your paper address subitem 2a-ii? *

Copy and paste relevant sections from the manuscript (include quotes in quotation marks "like this" to indicate direct quotes from your manuscript), or elaborate on this item by providing additional information not in the ms, or briefly explain why the item is not applicable/relevant for your study

"Background: Burnout among emergency room healthcare workers (HCWs) has reached critical levels, with up to 43% of all HCWs and 35% of emergency medicine personnel affected during the COVID-19 pandemic. Nurses were the most impacted, followed by physicians, leading to absenteeism, diminished care quality, and high turnover rates—reaching 78% in some settings, such as Thailand. Beyond workforce instability, burnout significantly compromises patient safety. Each one-unit increase in emotional exhaustion has been associated with a 2.63-fold increase in reports of poor care quality, a 30% rise in patient falls, a 47% increase in medication errors, and a 32% increase in healthcare-associated infections. Burnout is also linked to declining job satisfaction, worsening mental health, and increased intent to leave the profession. These findings highlight the urgent need for effective strategies to reduce stress and burnout in emergency care settings."

2b) In INTRODUCTION: Specific objectives or hypotheses

Does your paper address CONSORT subitem 2b? *

Copy and paste relevant sections from the manuscript (include quotes in quotation marks "like this" to indicate direct quotes from your manuscript), or elaborate on this item by providing additional information not in the ms, or briefly explain why the item is not applicable/relevant for your study

"Background: Burnout among emergency room healthcare workers (HCWs) has reached critical levels, with up to 43% of all HCWs and 35% of emergency medicine personnel affected during the COVID-19 pandemic. Nurses were the most impacted, followed by physicians, leading to absenteeism, diminished care quality, and high turnover rates—reaching 78% in some settings, such as Thailand. Beyond workforce instability, burnout significantly compromises patient safety. Each one-unit increase in emotional exhaustion has been associated with a 2.63-fold increase in reports of poor care quality, a 30% rise in patient falls, a 47% increase in medication errors, and a 32% increase in healthcare-associated infections. Burnout is also linked to declining job satisfaction, worsening mental health, and increased intent to leave the profession. These findings highlight the urgent need for effective strategies to reduce stress and burnout in emergency care settings."

METHODS

3a) Description of trial design (such as parallel, factorial) including allocation ratio

Does your paper address CONSORT subitem 3a? *

Copy and paste relevant sections from the manuscript (include quotes in quotation marks "like this" to indicate direct quotes from your manuscript), or elaborate on this item by providing additional information not in the ms, or briefly explain why the item is not applicable/relevant for your study

"Figure 1. Illustrates the CONSORT diagram [308] and participant flow throughout the study, which employed a three-Group treatment design."

3b) Important changes to methods after trial commencement (such as eligibility criteria), with reasons

Does your paper address CONSORT subitem 3b? *

Copy and paste relevant sections from the manuscript (include quotes in quotation marks "like this" to indicate direct quotes from your manuscript), or elaborate on this item by providing additional information not in the ms, or briefly explain why the item is not applicable/relevant for your study

"Figure 1. Illustrates the CONSORT diagram [308] and participant flow throughout the study, which employed a three-Group treatment design."

3b-i) Bug fixes, Downtimes, Content Changes

Bug fixes, Downtimes, Content Changes: ehealth systems are often dynamic systems. A description of changes to methods therefore also includes important changes made on the intervention or comparator during the trial (e.g., major bug fixes or changes in the functionality or content) (5-iii) and other "unexpected events" that may have influenced study design such as staff changes, system failures/downtimes, etc. [2].

	1	2	3	4	5	
subitem not at all important	(●)	○	○	○	○	essential

ล้างสิ่งที่เลือก

Does your paper address subitem 3b-i?

Copy and paste relevant sections from the manuscript (include quotes in quotation marks "like this" to indicate direct quotes from your manuscript), or elaborate on this item by providing additional information not in the ms, or briefly explain why the item is not applicable/relevant for your study

No major bug fixes, downtimes, or content changes occurred during the study; this subitem is not applicable to our intervention.

4a) Eligibility criteria for participants

Does your paper address CONSORT subitem 4a? *

Copy and paste relevant sections from the manuscript (include quotes in quotation marks "like this" to indicate direct quotes from your manuscript), or elaborate on this item by providing additional information not in the ms, or briefly explain why the item is not applicable/relevant for your study

"Figure 1. Illustrates the CONSORT diagram [308] and participant flow throughout the study, which employed a three-Group treatment design."

4a-i) Computer / Internet literacy

Computer / Internet literacy is often an implicit “de facto” eligibility criterion - this should be explicitly clarified.

	1	2	3	4	5	
subitem not at all important	◉	○	○	○	○	essential

ล้างสิ่งที่เลือก

Does your paper address subitem 4a-i?

Copy and paste relevant sections from the manuscript (include quotes in quotation marks "like this" to indicate direct quotes from your manuscript), or elaborate on this item by providing additional information not in the ms, or briefly explain why the item is not applicable/relevant for your study

This subitem is not applicable to our study as it does not involve the specific elements outlined in 4a-i.

4a-ii) Open vs. closed, web-based vs. face-to-face assessments:

Open vs. closed, web-based vs. face-to-face assessments: Mention how participants were recruited (online vs. offline), e.g., from an open access website or from a clinic, and clarify if this was a purely web-based trial, or there were face-to-face components (as part of the intervention or for assessment), i.e., to what degree got the study team to know the participant. In online-only trials, clarify if participants were quasi-anonymous and whether having multiple identities was possible or whether technical or logistical measures (e.g., cookies, email confirmation, phone calls) were used to detect/prevent these.

	1	2	3	4	5	
subitem not at all important	○	○	○	○	◉	essential

ล้างสิ่งที่เลือก

Does your paper address subitem 4a-ii? *

Copy and paste relevant sections from the manuscript (include quotes in quotation marks "like this" to indicate direct quotes from your manuscript), or elaborate on this item by providing additional information not in the ms, or briefly explain why the item is not applicable/relevant for your study

"After IRB approval, undergraduate clinical students from five healthcare disciplines (medicine, nursing, pharmacy, radiologic technology, and medical technology) were informed consent and recruited via announcements, Line, and posters. Interested students enrolled through a QR-linked Google Form. The principal investigator's contact was provided for inquiries. Participation was voluntary and scheduled outside of regular academic activities to avoid disruption."

4a-iii) Information giving during recruitment

Information given during recruitment. Specify how participants were briefed for recruitment and in the informed consent procedures (e.g., publish the informed consent documentation as appendix, see also item X26), as this information may have an effect on user self-selection, user expectation and may also bias results.

	1	2	3	4	5	
subitem not at all important	○	○	○	○	◉	essential

ล้างสิ่งที่เลือก

Does your paper address subitem 4a-iii?

Copy and paste relevant sections from the manuscript (include quotes in quotation marks "like this" to indicate direct quotes from your manuscript), or elaborate on this item by providing additional information not in the ms, or briefly explain why the item is not applicable/relevant for your study

"After IRB approval, undergraduate clinical students from five healthcare disciplines (medicine, nursing, pharmacy, radiologic technology, and medical technology) were informed consent and recruited via announcements, Line, and posters. Interested students enrolled through a QR-linked Google Form. The principal investigator's contact was provided for inquiries. Participation was voluntary and scheduled outside of regular academic activities to avoid disruption."

4b) Settings and locations where the data were collected

Does your paper address CONSORT subitem 4b? *

Copy and paste relevant sections from the manuscript (include quotes in quotation marks "like this" to indicate direct quotes from your manuscript), or elaborate on this item by providing additional information not in the ms, or briefly explain why the item is not applicable/relevant for your study

We extend our gratitude to the Chulalongkorn Healthcare Advanced Multi-Profession Simulation Center (CHAMPS) for their invaluable support

4b-i) Report if outcomes were (self-)assessed through online questionnaires

Clearly report if outcomes were (self-)assessed through online questionnaires (as common in web-based trials) or otherwise.

	1	2	3	4	5	
subitem not at all important	○	○	○	○	◉	essential

ล้างสิ่งที่เลือก

Does your paper address subitem 4b-i? *

Copy and paste relevant sections from the manuscript (include quotes in quotation marks "like this" to indicate direct quotes from your manuscript), or elaborate on this item by providing additional information not in the ms, or briefly explain why the item is not applicable/relevant for your study

"Outcome measures included the Dundee Stress State Questionnaire (DSSQ) and Copenhagen Burnout Inventory. Anxiety trait scores served as behavioral control. Self-reported stress and burnout levels were measured at baseline, pre-intervention, post-intervention, and one-month post-intervention. Generalized Estimating Equations (GEE) was employed to analyze variations in outcomes among the three groups. Statistical significance was set at P<.05."

4b-ii) Report how institutional affiliations are displayed

Report how institutional affiliations are displayed to potential participants [on ehealth media], as affiliations with prestigious hospitals or universities may affect volunteer rates, use, and reactions with regards to an intervention.(Not a required item – describe only if this may bias results)

	1	2	3	4	5	
subitem not at all important	○	○	○	○	◉	essential

ล้างสิ่งที่เลือก

Does your paper address subitem 4b-ii?

Copy and paste relevant sections from the manuscript (include quotes in quotation marks "like this" to indicate direct quotes from your manuscript), or elaborate on this item by providing additional information not in the ms, or briefly explain why the item is not applicable/relevant for your study

A single-blinded quasi-experimental study was conducted at a university hospital from July 2022 to September 2023.

5) The interventions for each group with sufficient details to allow replication, including how and when they were actually administered

5-i) Mention names, credential, affiliations of the developers, sponsors, and owners

Mention names, credential, affiliations of the developers, sponsors, and owners [6] (if authors/evaluators are owners or developer of the software, this needs to be declared in a “Conflict of interest” section or mentioned elsewhere in the manuscript).

	1	2	3	4	5	
subitem not at all important	○	○	○	○	◉	essential

ล้างสิ่งที่เลือก

Does your paper address subitem 5-i?

Copy and paste relevant sections from the manuscript (include quotes in quotation marks "like this" to indicate direct quotes from your manuscript), or elaborate on this item by providing additional information not in the ms, or briefly explain why the item is not applicable/relevant for your study

"Acknowledgments
This research was funded by the Second Century Fund, Chulalongkorn University. The authors declare no conflicts of interest. We extend our gratitude to the Chulalongkorn Healthcare Advanced Multi-Profession Simulation Center (CHAMPS) for their invaluable support, as well as Surachai Pianpetchlert, Thepwinphan Theppitak, and the research assistants—Kitnipat Boonydhammakul, Sutasinee Chaidej, Chayanit Trakulpipat, Sirisopha Suwanchinda, Thanyared Sangsawad, Chaiwat Takkanat, Thanes Yunirundorn, and Nuttarin Panswad—for their contributions to data collection, graphic design, and IT assistance.
We also thank the ER-VIPE study group for their collaboration in developing the movie, MOOCs, and SIMBIE platform. Special acknowledgment goes to Chanya Thanomlikhit, MNS, RN; Jiraphan Ritsamdang, B.Pharm, MD; Jennifer Chavanovanich, PhD; Kittisak Potisartra; Nattanun Chanchaochai, PhD; Nattawit Tanjapatkul; Navaporn Worasilchai, PhD; Pataraporn Kheawwan, PhD, RN; Porntiwa Sunpawut, MNS, RN; Sararas Kongwirojphan, PhD; Sawitree Suayod; Sopon Jakdetchai; Sujinat Jitwiriyanont, PhD; Suwimon Rojnawee, PhD; Thititip Tippayamontri, PhD; Tippayaporn Pavavimol; and Vishnu Kotrajaras, PhD, for their efforts in advancing this research."

5-ii) Describe the history/development process

Describe the history/development process of the application and previous formative evaluations (e.g., focus groups, usability testing), as these will have an impact on adoption/use rates and help with interpreting results.

	1	2	3	4	5	
subitem not at all important	○	○	○	○	◉	essential

ล้างสิ่งที่เลือก

Does your paper address subitem 5-ii?

Copy and paste relevant sections from the manuscript (include quotes in quotation marks "like this" to indicate direct quotes from your manuscript), or elaborate on this item by providing additional information not in the ms, or briefly explain why the item is not applicable/relevant for your study

"The 3D SIMBIE and 3D VR SIMBIE platforms were developed by our ER-VIPE team, which comprises professionals from various disciplines—including emergency physicians, nurses, pharmacists, medical technologists, radiologic technologists, communication arts specialists, instructional designers, architects, psychologists, and experts in the humanities and education. This interdisciplinary team collaborated with a specialized group of engineers in immersive learning technologies for healthcare education. Both platforms utilize advanced simulation software to create realistic, interactive environments that enable participants to engage in high-stakes medical scenarios."

5-iii) Revisions and updating

Revisions and updating. Clearly mention the date and/or version number of the application/intervention (and comparator, if applicable) evaluated, or describe whether the intervention underwent major changes during the evaluation process, or whether the development and/or content was “frozen” during the trial. Describe dynamic components such as news feeds or changing content which may have an impact on the replicability of the intervention (for unexpected events see item 3b).

	1	2	3	4	5	
subitem not at all important	(•)	( )	( )	( )	( )	essential

ล้างสิ่งที่เลือก

Does your paper address subitem 5-iii?

Copy and paste relevant sections from the manuscript (include quotes in quotation marks "like this" to indicate direct quotes from your manuscript), or elaborate on this item by providing additional information not in the ms, or briefly explain why the item is not applicable/relevant for your study

No, this item is not applicable because our intervention did not undergo major changes, version updates, or dynamic content modifications during the study period.

5-iv) Quality assurance methods

Provide information on quality assurance methods to ensure accuracy and quality of information provided [1], if applicable.

	1	2	3	4	5	
subitem not at all important	◉	○	○	○	○	essential

ล้างสิ่งที่เลือก

Does your paper address subitem 5-iv?

Copy and paste relevant sections from the manuscript (include quotes in quotation marks "like this" to indicate direct quotes from your manuscript), or elaborate on this item by providing additional information not in the ms, or briefly explain why the item is not applicable/relevant for your study

No, subitem 5-iv is not applicable because the intervention did not include adaptive components, tailored feedback, or real-time personalization during the study.

5-v) Ensure replicability by publishing the source code, and/or providing screenshots/screen-capture video, and/or providing flowcharts of the algorithms used

Ensure replicability by publishing the source code, and/or providing screenshots/screen-capture video, and/or providing flowcharts of the algorithms used. Replicability (i.e., other researchers should in principle be able to replicate the study) is a hallmark of scientific reporting.

	1	2	3	4	5	
subitem not at all important	◉	○	○	○	○	essential

ล้างสิ่งที่เลือก

Does your paper address subitem 5-v?

Copy and paste relevant sections from the manuscript (include quotes in quotation marks "like this" to indicate direct quotes from your manuscript), or elaborate on this item by providing additional information not in the ms, or briefly explain why the item is not applicable/relevant for your study

No, this item is not addressed in the manuscript because the platform source code and algorithm details are protected under intellectual property and patent confidentiality agreements.

5-vi) Digital preservation

Digital preservation: Provide the URL of the application, but as the intervention is likely to change or disappear over the course of the years; also make sure the intervention is archived (Internet Archive, webcitation.org, and/or publishing the source code or screenshots/videos alongside the article). As pages behind login screens cannot be archived, consider creating demo pages which are accessible without login.

	1	2	3	4	5	
subitem not at all important	◉	○	○	○	○	essential

ล้างสิ่งที่เลือก

Does your paper address subitem 5-vi?

Copy and paste relevant sections from the manuscript (include quotes in quotation marks "like this" to indicate direct quotes from your manuscript), or elaborate on this item by providing additional information not in the ms, or briefly explain why the item is not applicable/relevant for your study

No, this item is not addressed in the manuscript because the platform source code and algorithm details are protected under intellectual property and patent confidentiality agreements.

5-vii) Access

Access: Describe how participants accessed the application, in what setting/context, if they had to pay (or were paid) or not, whether they had to be a member of specific group. If known, describe how participants obtained “access to the platform and Internet” [1]. To ensure access for editors/reviewers/readers, consider to provide a “backdoor” login account or demo mode for reviewers/readers to explore the application (also important for archiving purposes, see vi).

	1	2	3	4	5	
subitem not at all important	(●)	○	○	○	○	essential

ล้างสิ่งที่เลือก

Does your paper address subitem 5-vii? *

Copy and paste relevant sections from the manuscript (include quotes in quotation marks "like this" to indicate direct quotes from your manuscript), or elaborate on this item by providing additional information not in the ms, or briefly explain why the item is not applicable/relevant for your study

No, this item is not addressed in the manuscript because the platform source code and algorithm details are protected under intellectual property and patent confidentiality agreements. Yes, monetary incentives were offered. Participants received 500 Baht per session. This compensation was provided to acknowledge their time and contribution. No other prizes or non-monetary incentives (such as access to survey results) were offered. Participation was entirely voluntary and did not interfere with students’ regular academic schedules

5-viii) Mode of delivery, features/functionalities/components of the intervention and comparator, and the theoretical framework

Describe mode of delivery, features/functionalities/components of the intervention and comparator, and the theoretical framework [6] used to design them (instructional strategy [1], behaviour change techniques, persuasive features, etc., see e.g., [7, 8] for terminology). This includes an in-depth description of the content (including where it is coming from and who developed it) [1]," whether [and how] it is tailored to individual circumstances and allows users to track their progress and receive feedback" [6]. This also includes a description of communication delivery channels and – if computer-mediated communication is a component – whether communication was synchronous or asynchronous [6]. It also includes information on presentation strategies [1], including page design principles, average amount of text on pages, presence of hyperlinks to other resources, etc. [1].

	1	2	3	4	5	
subitem not at all important	○	○	○	○	◉	essential

ล้างสิ่งที่เลือก

Does your paper address subitem 5-viii? *

Copy and paste relevant sections from the manuscript (include quotes in quotation marks "like this" to indicate direct quotes from your manuscript), or elaborate on this item by providing additional information not in the ms, or briefly explain why the item is not applicable/relevant for your study

"An interactive game-based interface further reinforced TeamSTEPPS competencies as our main learning objectives. Each participant was equipped with an HP Bar, a symbolic "health point" indicator representing emotional resilience under pressure. The HP Bar depleted over time, particularly during high-stakes decision points or delays in clinical action such as managing a “can’t intubate, can’t ventilate” situation, video laryngoscope battery failure, or sudden hypotension. Mutual support was emphasized as teammates could replenish each other’s HP by pressing a “plus” button—an interactive metaphor for peer support and collaborative coping. The HP Bar thus served both as a visual representation of stress load and as a positive reinforcement mechanism for TeamSTEPPS-aligned behaviors. These features were designed to enhance psychological fidelity and simulate cognitive load realism by integrating both clinical complexity and emotional dynamics. " "The 3D SIMBIE and 3D VR SIMBIE platforms were developed by our ER-VIPE team, which comprises professionals from various disciplines—including emergency physicians, nurses, pharmacists, medical technologists, radiologic technologists, communication arts specialists, instructional designers, architects, psychologists, and experts in the humanities and education. This interdisciplinary team collaborated with a specialized group of engineers in immersive learning technologies for healthcare education. Both platforms utilize advanced simulation software to create realistic, interactive environments that enable participants to engage in high-stakes medical scenarios." " The key distinction lay in the level of immersion: the 3D version utilized standard computer input devices (e.g., screen, keyboard, and mouse), while VR-SIMBIE employed headsets and controllers to deepen emotional engagement and presence. This allowed participants to physically move and interact within the environment in a fully immersive and realistic manner. The VR format enhances the sense of presence, making participants feel as though they are “inside” the clinical scenario, which may result in greater cognitive and emotional stress compared to the 3D desktop version. Figures 2 and 3 illustrate the user interface, visual comparisons, and experiential differences between both platforms. Each simulation concluded with a structured co-debriefing session, enabling participants to reflect on clinical decision-making and TeamSTEPPS performance, consolidating targeted non-technical teamwork competencies."

5-ix) Describe use parameters

Describe use parameters (e.g., intended “doses” and optimal timing for use). Clarify what instructions or recommendations were given to the user, e.g., regarding timing, frequency, heaviness of use, if any, or was the intervention used ad libitum.

	1	2	3	4	5	
subitem not at all important	○	○	○	○	◉	essential

ล้างสิ่งที่เลือก

Does your paper address subitem 5-ix?

Copy and paste relevant sections from the manuscript (include quotes in quotation marks "like this" to indicate direct quotes from your manuscript), or elaborate on this item by providing additional information not in the ms, or briefly explain why the item is not applicable/relevant for your study

"This study employed a two-arm design with Groups B and C as the experimental groups. Both groups underwent a series of interventions, including a combination of Medical Movie, a MOOCs, and a 3D SIMBIE Simulation, followed by an co-debriefing session, collectively referred to as ER-VIPE. Group B utilized a computer-based 3D SIMBIE for approximately 30 minutes, while Group C used a 3D virtual reality (VR) 3D SIMBIE for the same duration. Both groups participated in a 60-minute oral co-debriefing session (see Figure 1)."

5-x) Clarify the level of human involvement

Clarify the level of human involvement (care providers or health professionals, also technical assistance) in the e-intervention or as co-intervention (detail number and expertise of professionals involved, if any, as well as “type of assistance offered, the timing and frequency of the support, how it is initiated, and the medium by which the assistance is delivered”. It may be necessary to distinguish between the level of human involvement required for the trial, and the level of human involvement required for a routine application outside of a RCT setting (discuss under item 21 – generalizability).

	1	2	3	4	5	
subitem not at all important	○	○	○	○	◉	essential

ล้างสิ่งที่เลือก

Does your paper address subitem 5-x?

Copy and paste relevant sections from the manuscript (include quotes in quotation marks "like this" to indicate direct quotes from your manuscript), or elaborate on this item by providing additional information not in the ms, or briefly explain why the item is not applicable/relevant for your study

"This study employed a two-arm design with Groups B and C as the experimental groups. Both groups underwent a series of interventions, including a combination of Medical Movie, a MOOCs, and a 3D SIMBIE Simulation, followed by an co-debriefing session, collectively referred to as ER-VIPE. Group B utilized a computer-based 3D SIMBIE for approximately 30 minutes, while Group C used a 3D virtual reality (VR) 3D SIMBIE for the same duration. Both groups participated in a 60-minute oral co-debriefing session (see Figure 1)."

5-xi) Report any prompts/reminders used

Report any prompts/reminders used: Clarify if there were prompts (letters, emails, phone calls, SMS) to use the application, what triggered them, frequency etc. It may be necessary to distinguish between the level of prompts/reminders required for the trial, and the level of prompts/reminders for a routine application outside of a RCT setting (discuss under item 21 – generalizability).

	1	2	3	4	5	
subitem not at all important	○	○	○	○	◉	essential

ล้างสิ่งที่เลือก

Does your paper address subitem 5-xi? *

Copy and paste relevant sections from the manuscript (include quotes in quotation marks "like this" to indicate direct quotes from your manuscript), or elaborate on this item by providing additional information not in the ms, or briefly explain why the item is not applicable/relevant for your study

Yes, usability issues were explored through interviews, open-ended feedback, and satisfaction questionnaires as part of a mixed-methods study. These data were collected and analyzed to better understand users' experiences. The findings are currently under review for publication in the Journal of Medical Internet Research (JMIR).

5-xii) Describe any co-interventions (incl. training/support)

Describe any co-interventions (incl. training/support): Clearly state any interventions that are provided in addition to the targeted eHealth intervention, as ehealth intervention may not be designed as stand-alone intervention. This includes training sessions and support [1]. It may be necessary to distinguish between the level of training required for the trial, and the level of training for a routine application outside of a RCT setting (discuss under item 21 – generalizability.

	1	2	3	4	5	
subitem not at all important	◉	○	○	○	○	essential

ล้างสิ่งที่เลือก

Does your paper address subitem 5-xii? *

Copy and paste relevant sections from the manuscript (include quotes in quotation marks "like this" to indicate direct quotes from your manuscript), or elaborate on this item by providing additional information not in the ms, or briefly explain why the item is not applicable/relevant for your study

No co-interventions such as training or support were provided beyond the core eHealth intervention during this study.

6a) Completely defined pre-specified primary and secondary outcome measures, including how and when they were assessed

Does your paper address CONSORT subitem 6a? *

Copy and paste relevant sections from the manuscript (include quotes in quotation marks "like this" to indicate direct quotes from your manuscript), or elaborate on this item by providing additional information not in the ms, or briefly explain why the item is not applicable/relevant for your study

" Objective: This study aimed to evaluate the effectiveness and effect size of a multimodal learning approach—Emergency Room Virtual Simulation Interprofessional Education (ER-VIPE)—which integrates medical movies, MOOCs, and computer- or VR-based simulations with co-debriefing, in improving self-reported stress levels and reducing burnout among future healthcare professionals, compared to alternative methods lacking co-debriefing or using only movies and MOOCs.

Methods: A single-blinded quasi-experimental study was conducted at a university hospital from July 2022 to September 2023. A three-Group treatment design, Group A (control) participated in a 3D computer-based simulation-based interprofessional education (SIMBIE) without debriefing. Group B received a medical movie, MOOCs, a 3D computer-based SIMBIE, collectively referred to as ER-VIPE (Emergency Room Virtual Interprofessional Education). and an co-debriefing session. Group C received the same interventions as Group B, except the 3D computer-based SIMBIE was replaced with a 3D virtual reality SIMBIE. SIMBIE experiential learning activities involving a COVID-19 pneumonia crisis scenario. Outcome measures included the Dundee Stress State Questionnaire (DSSQ) and Copenhagen Burnout Inventory. Anxiety trait scores served as behavioral control. Self-reported stress and burnout levels were measured at baseline, pre-intervention, post-intervention, and one-month post-intervention. Generalized Estimating Equations (GEE) was employed to analyze variations in outcomes among the three groups. Statistical significance was set at P<.05."

6a-i) Online questionnaires: describe if they were validated for online use and apply CHERRIES items to describe how the questionnaires were designed/deployed

If outcomes were obtained through online questionnaires, describe if they were validated for online use and apply CHERRIES items to describe how the questionnaires were designed/deployed [9].

	1	2	3	4	5	
subitem not at all important	○	○	○	○	◉	essential

ล้างสิ่งที่เลือก

Does your paper address subitem 6a-i?

Copy and paste relevant sections from manuscript text

Yes, CHERRIES guidelines were applied to describe the design and deployment of the online questionnaires. Although the original instruments had been previously validated in traditional formats, their online use followed established CHERRIES protocols to ensure consistency, clarity, and respondent usability.

6a-ii) Describe whether and how “use” (including intensity of use/dosage) was defined/measured/monitored

Describe whether and how “use” (including intensity of use/dosage) was defined/measured/monitored (logins, logfile analysis, etc.). Use/adoption metrics are important process outcomes that should be reported in any ehealth trial.

	1	2	3	4	5	
subitem not at all important	◉	○	○	○	○	essential

ล้างสิ่งที่เลือก

Does your paper address subitem 6a-ii?

Copy and paste relevant sections from manuscript text

No, the intensity of use or engagement was not measured or monitored in this study.

6a-iii) Describe whether, how, and when qualitative feedback from participants was obtained

Describe whether, how, and when qualitative feedback from participants was obtained (e.g., through emails, feedback forms, interviews, focus groups).

	1	2	3	4	5	
subitem not at all important	○	○	○	○	○	essential

Does your paper address subitem 6a-iii?

Copy and paste relevant sections from manuscript text

Yes, qualitative feedback was obtained through focus group interviews and open-ended questions in post-intervention surveys; however, these findings are reported in a separate manuscript currently under review.

6b) Any changes to trial outcomes after the trial commenced, with reasons

Does your paper address CONSORT subitem 6b? *

Copy and paste relevant sections from the manuscript (include quotes in quotation marks "like this" to indicate direct quotes from your manuscript), or elaborate on this item by providing additional information not in the ms, or briefly explain why the item is not applicable/relevant for your study

No changes were made to the trial outcomes after the trial commenced.

7a) How sample size was determined

NPT: When applicable, details of whether and how the clustering by care provides or centers was addressed

7a-i) Describe whether and how expected attrition was taken into account when calculating the sample size

Describe whether and how expected attrition was taken into account when calculating the sample size.

	1	2	3	4	5	
subitem not at all important	○	○	○	○	◉	essential

ล้างสิ่งที่เลือก

Does your paper address subitem 7a-i?

Copy and paste relevant sections from manuscript title (include quotes in quotation marks "like this" to indicate direct quotes from your manuscript), or elaborate on this item by providing additional information not in the ms, or briefly explain why the item is not applicable/relevant for your study

"Additionally, dropout rates of 24%, 44%, and 14% in Groups A, B, and C, respectively, during the transition from Phase 2 to Phase 3 were addressed. We did not know the exact reasons for participant dropout. However, potential contributing factors may include exam schedules, personal or academic stress, mental health concerns, and minor technical issues related to the online survey platform sent via email. Additionally, the delayed delivery of monetary incentives—provided only after the second simulation, several weeks before the final assessment—may have reduced participants' motivation to complete the study. We using Generalized Estimating Equations (GEE) to minimize sensitivity to missing data. An intention-to-treat analysis with imputed data was also performed to validate the per-protocol findings. This indicates that even though the dropout rate was low, the results were likely not significantly different from the original findings. "

7b) When applicable, explanation of any interim analyses and stopping guidelines

Does your paper address CONSORT subitem 7b? *

Copy and paste relevant sections from the manuscript (include quotes in quotation marks "like this" to indicate direct quotes from your manuscript), or elaborate on this item by providing additional information not in the ms, or briefly explain why the item is not applicable/relevant for your study

There were no interim analyses or stopping guidelines applied during the study, as it followed a predefined protocol without planned interruptions.

8a) Method used to generate the random allocation sequence

NPT: When applicable, how care providers were allocated to each trial group

Does your paper address CONSORT subitem 8a? *

Copy and paste relevant sections from the manuscript (include quotes in quotation marks "like this" to indicate direct quotes from your manuscript), or elaborate on this item by providing additional information not in the ms, or briefly explain why the item is not applicable/relevant for your study

"Participants were allocated into three groups (A, B, and C) using a stratified convenience sampling method to ensure balance in baseline characteristics such as age, education level, and prior clinical experience. Each group was further divided into five interprofessional subgroups, with each subgroup consisting of one medical student, two nursing students, one pharmacy student, one medical technologist student, and one radiological technologist student. "

8b) Type of randomisation; details of any restriction (such as blocking and block size)

Does your paper address CONSORT subitem 8b? *

Copy and paste relevant sections from the manuscript (include quotes in quotation marks "like this" to indicate direct quotes from your manuscript), or elaborate on this item by providing additional information not in the ms, or briefly explain why the item is not applicable/relevant for your study

"Participants were allocated into three groups (A, B, and C) using a stratified convenience sampling method to ensure balance in baseline characteristics such as age, education level, and prior clinical experience. Each group was further divided into five interprofessional subgroups, with each subgroup consisting of one medical student, two nursing students, one pharmacy student, one medical technologist student, and one radiological technologist student. "

9) Mechanism used to implement the random allocation sequence (such as sequentially numbered containers), describing any steps taken to conceal the sequence until interventions were assigned

Does your paper address CONSORT subitem 9? *

Copy and paste relevant sections from the manuscript (include quotes in quotation marks "like this" to indicate direct quotes from your manuscript), or elaborate on this item by providing additional information not in the ms, or briefly explain why the item is not applicable/relevant for your study

No random allocation concealment was applied; the mechanism for sequence implementation was not used in this study.

10) Who generated the random allocation sequence, who enrolled participants, and who assigned participants to interventions

Does your paper address CONSORT subitem 10? *

Copy and paste relevant sections from the manuscript (include quotes in quotation marks "like this" to indicate direct quotes from your manuscript), or elaborate on this item by providing additional information not in the ms, or briefly explain why the item is not applicable/relevant for your study

This study did not involve random allocation; therefore, no individual was responsible for generating a random sequence, enrolling participants, or assigning them to intervention groups.

11a) If done, who was blinded after assignment to interventions (for example, participants, care providers, those assessing outcomes) and how
NPT: Whether or not administering co-interventions were blinded to group assignment

11a-i) Specify who was blinded, and who wasn't

Specify who was blinded, and who wasn't. Usually, in web-based trials it is not possible to blind the participants [1, 3] (this should be clearly acknowledged), but it may be possible to blind outcome assessors, those doing data analysis or those administering co-interventions (if any).

	1	2	3	4	5	
subitem not at all important	○	○	○	○	◉	essential

ล้างสิ่งที่เลือก

Does your paper address subitem 11a-i? *

Copy and paste relevant sections from the manuscript (include quotes in quotation marks "like this" to indicate direct quotes from your manuscript), or elaborate on this item by providing additional information not in the ms, or briefly explain why the item is not applicable/relevant for your study

"To minimize bias, data collection and analysis were performed by a single-blinded statistician."

11a-ii) Discuss e.g., whether participants knew which intervention was the “intervention of interest” and which one was the “comparator”

Informed consent procedures (4a-ii) can create biases and certain expectations - discuss e.g., whether participants knew which intervention was the “intervention of interest” and which one was the “comparator”.

	1	2	3	4	5	
subitem not at all important	○	○	○	○	◉	essential

ล้างสิ่งที่เลือก

Does your paper address subitem 11a-ii?

Copy and paste relevant sections from the manuscript (include quotes in quotation marks "like this" to indicate direct quotes from your manuscript), or elaborate on this item by providing additional information not in the ms, or briefly explain why the item is not applicable/relevant for your study

Participants were aware of their assigned intervention group (e.g., computer-based vs. VR headset), as it was not feasible to blind them due to the nature of the delivery platforms.

11b) If relevant, description of the similarity of interventions

(this item is usually not relevant for ehealth trials as it refers to similarity of a placebo or sham intervention to a active medication/intervention)

Does your paper address CONSORT subitem 11b? *

Copy and paste relevant sections from the manuscript (include quotes in quotation marks "like this" to indicate direct quotes from your manuscript), or elaborate on this item by providing additional information not in the ms, or briefly explain why the item is not applicable/relevant for your study

Answer: Not applicable to this study, as no placebo or sham intervention was used. Each intervention group involved clearly distinct formats—such as the use of VR headsets or 3D computer-based learning—making similarity between interventions irrelevant.

12a) Statistical methods used to compare groups for primary and secondary outcomes

NPT: When applicable, details of whether and how the clustering by care providers or centers was addressed

Does your paper address CONSORT subitem 12a? *

Copy and paste relevant sections from the manuscript (include quotes in quotation marks "like this" to indicate direct quotes from your manuscript), or elaborate on this item by providing additional information not in the ms, or briefly explain why the item is not applicable/relevant for your study

No clustering by care providers or centers was applicable or addressed in this study.

12a-i) Imputation techniques to deal with attrition / missing values

Imputation techniques to deal with attrition / missing values: Not all participants will use the intervention/comparator as intended and attrition is typically high in ehealth trials. Specify how participants who did not use the application or dropped out from the trial were treated in the statistical analysis (a complete case analysis is strongly discouraged, and simple imputation techniques such as LOCF may also be problematic [4]).

	1	2	3	4	5	
subitem not at all important	○	○	○	○	◉	essential

ล้างสิ่งที่เลือก

Does your paper address subitem 12a-i? *

Copy and paste relevant sections from the manuscript (include quotes in quotation marks "like this" to indicate direct quotes from your manuscript), or elaborate on this item by providing additional information not in the ms, or briefly explain why the item is not applicable/relevant for your study

"Both an intention-to-treat (ITT) analysis and a per-protocol (PP) analysis were performed. Missing data were imputed using the Last Observation Carried Forward (LOCF) method. Statistical significance was set at a two-tailed P value of <.05 for all analyses. Stata version 15 [331] was used for data analyses."

12b) Methods for additional analyses, such as subgroup analyses and adjusted analyses

Does your paper address CONSORT subitem 12b? *

Copy and paste relevant sections from the manuscript (include quotes in quotation marks "like this" to indicate direct quotes from your manuscript), or elaborate on this item by providing additional information not in the ms, or briefly explain why the item is not applicable/relevant for your study

"Statistical analysis was conducted using Generalized Estimating Equations (GEE), adjusted for anxiety traits as a control variable, reveals intervention effects based on an intention-to-treat analysis."

X26) REB/IRB Approval and Ethical Considerations [recommended as subheading under "Methods"] (not a CONSORT item)

X26-i) Comment on ethics committee approval

	1	2	3	4	5	
subitem not at all important	○	○	○	○	◉	essential

ล้างสิ่งที่เลือก

Does your paper address subitem X26-i?

Copy and paste relevant sections from the manuscript (include quotes in quotation marks "like this" to indicate direct quotes from your manuscript), or elaborate on this item by providing additional information not in the ms, or briefly explain why the item is not applicable/relevant for your study

"The study protocol was approved by the Ethics Committee for Research in Human Subjects of the Faculty of Medicine, Chulalongkorn University, Thailand (IRB No. 0366/65). Participants were informed about the study's objectives, procedures, potential risks, and benefits. This information was provided both orally and in writing before obtaining informed consent. Participants were assured of their right to make voluntary decisions and withdraw from the study at any time. Data was anonymized to maintain confidentiality."

x26-ii) Outline informed consent procedures

Outline informed consent procedures e.g., if consent was obtained offline or online (how? Checkbox, etc.?), and what information was provided (see 4a-ii). See [6] for some items to be included in informed consent documents.

	1	2	3	4	5	
subitem not at all important	○	○	○	○	◉	essential

ล้างสิ่งที่เลือก

Does your paper address subitem X26-ii?

Copy and paste relevant sections from the manuscript (include quotes in quotation marks "like this" to indicate direct quotes from your manuscript), or elaborate on this item by providing additional information not in the ms, or briefly explain why the item is not applicable/relevant for your study

"After IRB approval, undergraduate clinical students from five healthcare disciplines (medicine, nursing, pharmacy, radiologic technology, and medical technology) were informed consent and recruited via announcements, Line, and posters. Interested students enrolled through a QR-linked Google Form. The principal investigator's contact was provided for inquiries. Participation was voluntary and scheduled outside of regular academic activities to avoid disruption."

X26-iii) Safety and security procedures

Safety and security procedures, incl. privacy considerations, and any steps taken to reduce the likelihood or detection of harm (e.g., education and training, availability of a hotline)

	1	2	3	4	5	
subitem not at all important	○	○	○	○	○	essential

Does your paper address subitem X26-iii?

Copy and paste relevant sections from the manuscript (include quotes in quotation marks "like this" to indicate direct quotes from your manuscript), or elaborate on this item by providing additional information not in the ms, or briefly explain why the item is not applicable/relevant for your study

"Ethical Approval
The study protocol was approved by the Ethics Committee for Research in Human Subjects of the Faculty of Medicine, Chulalongkorn University, Thailand (IRB No. 0366/65). Participants were informed about the study's objectives, procedures, potential risks, and benefits. This information was provided both orally and in writing before obtaining informed consent. Participants were assured of their right to make voluntary decisions and withdraw from the study at any time. Data was anonymized to maintain confidentiality."

RESULTS

13a) For each group, the numbers of participants who were randomly assigned, received intended treatment, and were analysed for the primary outcome

NPT: The number of care providers or centers performing the intervention in each group and the number of patients treated by each care provider in each center

Does your paper address CONSORT subitem 13a? *

Copy and paste relevant sections from the manuscript (include quotes in quotation marks "like this" to indicate direct quotes from your manuscript), or elaborate on this item by providing additional information not in the ms, or briefly explain why the item is not applicable/relevant for your study

"A total of 87 undergraduate clinical students from various professional programs participated in the study, with 29 students in each group (A, B, and C)."

13b) For each group, losses and exclusions after randomisation, together with reasons

Does your paper address CONSORT subitem 13b? (NOTE: Preferably, this is shown in a CONSORT flow diagram) *

Copy and paste relevant sections from the manuscript (include quotes in quotation marks "like this" to indicate direct quotes from your manuscript), or elaborate on this item by providing additional information not in the ms, or briefly explain why the item is not applicable/relevant for your study

"Additionally, dropout rates of 24%, 44%, and 14% in Groups A, B, and C, respectively, during the transition from Phase 2 to Phase 3 were addressed. We did not know the exact reasons for participant dropout. However, potential contributing factors may include exam schedules, personal or academic stress, mental health concerns, and minor technical issues related to the online survey platform sent via email. Additionally, the delayed delivery of monetary incentives—provided only after the second simulation, several weeks before the final assessment—may have reduced participants' motivation to complete the study. "

13b-i) Attrition diagram

Strongly recommended: An attrition diagram (e.g., proportion of participants still logging in or using the intervention/comparator in each group plotted over time, similar to a survival curve) or other figures or tables demonstrating usage/dose/engagement.

	1	2	3	4	5	
subitem not at all important	○	○	○	○	◉	essential

ล้างสิ่งที่เลือก

Does your paper address subitem 13b-i?

Copy and paste relevant sections from the manuscript or cite the figure number if applicable (include quotes in quotation marks "like this" to indicate direct quotes from your manuscript), or elaborate on this item by providing additional information not in the ms, or briefly explain why the item is not applicable/relevant for your study

"Figure 1. Illustrates the CONSORT diagram [308] and participant flow throughout the study, which employed a three-Group treatment design. "

14a) Dates defining the periods of recruitment and follow-up

Does your paper address CONSORT subitem 14a? *

Copy and paste relevant sections from the manuscript (include quotes in quotation marks "like this" to indicate direct quotes from your manuscript), or elaborate on this item by providing additional information not in the ms, or briefly explain why the item is not applicable/relevant for your study

"Figure 1. Illustrates the CONSORT diagram [308] and participant flow throughout the study, which employed a three-Group treatment design. Group A (control) participated in a 3D computer-based SIMBIE without oral debriefing.)."

14a-i) Indicate if critical “secular events” fell into the study period

Indicate if critical “secular events” fell into the study period, e.g., significant changes in Internet resources available or “changes in computer hardware or Internet delivery resources”

	1	2	3	4	5	
subitem not at all important	○	○	○	○	◉	essential

ล้างสิ่งที่เลือก

Does your paper address subitem 14a-i?

Copy and paste relevant sections from the manuscript (include quotes in quotation marks "like this" to indicate direct quotes from your manuscript), or elaborate on this item by providing additional information not in the ms, or briefly explain why the item is not applicable/relevant for your study

"However, a quasi-experimental design was adopted due to practical constraints in randomizing participants within a clinical setting during the COVID-19 pandemic. "

14b) Why the trial ended or was stopped (early)

Does your paper address CONSORT subitem 14b? *

Copy and paste relevant sections from the manuscript (include quotes in quotation marks "like this" to indicate direct quotes from your manuscript), or elaborate on this item by providing additional information not in the ms, or briefly explain why the item is not applicable/relevant for your study

The trial was completed as planned upon reaching the target number of participants; it was not stopped early.

15) A table showing baseline demographic and clinical characteristics for each group

NPT: When applicable, a description of care providers (case volume, qualification, expertise, etc.) and centers (volume) in each group

Does your paper address CONSORT subitem 15? *

Copy and paste relevant sections from the manuscript (include quotes in quotation marks "like this" to indicate direct quotes from your manuscript), or elaborate on this item by providing additional information not in the ms, or briefly explain why the item is not applicable/relevant for your study

"Demographic
A total of 87 undergraduate clinical students from various professional programs participated in the study, with 29 students in each group (A, B, and C). The sample was predominantly female (71%) with a mean age of 21.87 years (SD = 1.13). No significant differences were found in demographic characteristics between the groups. However, analysis of debriefing duration revealed significant differences in the mean and standard deviation between Group B (mean = 50.93, SD = 12.61) and Group C (mean = 60.33, SD = 9.75), as shown in Table 1."

15-i) Report demographics associated with digital divide issues

In ehealth trials it is particularly important to report demographics associated with digital divide issues, such as age, education, gender, social-economic status, computer/Internet/ehealth literacy of the participants, if known.

	1	2	3	4	5	
subitem not at all important	○	○	○	◉	○	essential

ล้างสิ่งที่เลือก

Does your paper address subitem 15-i? *

Copy and paste relevant sections from the manuscript (include quotes in quotation marks "like this" to indicate direct quotes from your manuscript), or elaborate on this item by providing additional information not in the ms, or briefly explain why the item is not applicable/relevant for your study

"A total of 87 undergraduate clinical students from various professional programs participated in the study, with 29 students in each group (A, B, and C). The sample was predominantly female (71%) with a mean age of 21.87 years (SD = 1.13). "

16) For each group, number of participants (denominator) included in each analysis and whether the analysis was by original assigned groups

16-i) Report multiple “denominators” and provide definitions

Report multiple “denominators” and provide definitions: Report N’s (and effect sizes) “across a range of study participation [and use] thresholds” [1], e.g., N exposed, N consented, N used more than x times, N used more than y weeks, N participants “used” the intervention/comparator at specific pre-defined time points of interest (in absolute and relative numbers per group). Always clearly define “use” of the intervention.

	1	2	3	4	5	
subitem not at all important	○	○	○	○	◉	essential

ล้างสิ่งที่เลือก

Does your paper address subitem 16-i? *

Copy and paste relevant sections from the manuscript (include quotes in quotation marks "like this" to indicate direct quotes from your manuscript), or elaborate on this item by providing additional information not in the ms, or briefly explain why the item is not applicable/relevant for your study

"A total of 87 undergraduate clinical students from various professional programs participated in the study, with 29 students in each group (A, B, and C). " Yes, the analysis was conducted based on the originally assigned groups (intention-to-treat).

16-ii) Primary analysis should be intent-to-treat

Primary analysis should be intent-to-treat, secondary analyses could include comparing only “users”, with the appropriate caveats that this is no longer a randomized sample (see 18-i).

	1	2	3	4	5	
subitem not at all important	○	○	○	○	◉	essential

ล้างสิ่งที่เลือก

Does your paper address subitem 16-ii?

Copy and paste relevant sections from the manuscript (include quotes in quotation marks "like this" to indicate direct quotes from your manuscript), or elaborate on this item by providing additional information not in the ms, or briefly explain why the item is not applicable/relevant for your study

"These results were obtained through an intention-to-treat analysis (see Figure 4, Figure 5, and Table 3 in Multimedia Appendix 4). A per-protocol analysis further corroborated the findings from the intention-to-treat analysis, demonstrating consistent results and interpretations for the mean change in DSSQ scores (see Table 4 in Multimedia Appendix 5, Figure 1 in Multimedia Appendix 6, and Figure 2 in Multimedia Appendix 7)."

17a) For each primary and secondary outcome, results for each group, and the estimated effect size and its precision (such as 95% confidence interval)

Does your paper address CONSORT subitem 17a? *

Copy and paste relevant sections from the manuscript (include quotes in quotation marks "like this" to indicate direct quotes from your manuscript), or elaborate on this item by providing additional information not in the ms, or briefly explain why the item is not applicable/relevant for your study

"The ER-VIPE computer-based intervention (Group B) at 8 weeks demonstrated the largest reduction, with a small-to-moderate effect size (d = - 0.31; 95% CI: –.78 to .15).

17a-i) Presentation of process outcomes such as metrics of use and intensity of use

In addition to primary/secondary (clinical) outcomes, the presentation of process outcomes such as metrics of use and intensity of use (dose, exposure) and their operational definitions is critical. This does not only refer to metrics of attrition (13-b) (often a binary variable), but also to more continuous exposure metrics such as “average session length”. These must be accompanied by a technical description how a metric like a “session” is defined (e.g., timeout after idle time) [1] (report under item 6a).

	1	2	3	4	5	
subitem not at all important	(●)	( )	( )	( )	( )	essential

ล้างสิ่งที่เลือก

Does your paper address subitem 17a-i?

Copy and paste relevant sections from the manuscript (include quotes in quotation marks "like this" to indicate direct quotes from your manuscript), or elaborate on this item by providing additional information not in the ms, or briefly explain why the item is not applicable/relevant for your study

No process outcomes such as use or intensity metrics were collected or analyzed in this study.

17b) For binary outcomes, presentation of both absolute and relative effect sizes is recommended

Does your paper address CONSORT subitem 17b? *

Copy and paste relevant sections from the manuscript (include quotes in quotation marks "like this" to indicate direct quotes from your manuscript), or elaborate on this item by providing additional information not in the ms, or briefly explain why the item is not applicable/relevant for your study

"The ER-VIPE computer-based intervention (Group B) at 8 weeks demonstrated the largest reduction, with a small-to-moderate effect size (d = - 0.31; 95% CI: −.78 to .15).

18) Results of any other analyses performed, including subgroup analyses and adjusted analyses, distinguishing pre-specified from exploratory

Does your paper address CONSORT subitem 18? *

Copy and paste relevant sections from the manuscript (include quotes in quotation marks "like this" to indicate direct quotes from your manuscript), or elaborate on this item by providing additional information not in the ms, or briefly explain why the item is not applicable/relevant for your study

"To assess changes over time and compare improvements in burnout and DSSQ scores between groups, GEE were employed, with adjustments for anxiety as a covariate. Both an intention-to-treat (ITT) analysis and a per-protocol (PP) analysis were performed." No, only exploratory analyses were conducted without pre-specified subgroup or adjusted analyses.

18-i) Subgroup analysis of comparing only users

A subgroup analysis of comparing only users is not uncommon in ehealth trials, but if done, it must be stressed that this is a self-selected sample and no longer an unbiased sample from a randomized trial (see 16-iii).

	1	2	3	4	5	
subitem not at all important	◉	○	○	○	○	essential

ล้างสิ่งที่เลือก

Does your paper address subitem 18-i?

Copy and paste relevant sections from the manuscript (include quotes in quotation marks "like this" to indicate direct quotes from your manuscript), or elaborate on this item by providing additional information not in the ms, or briefly explain why the item is not applicable/relevant for your study

"No, subgroup analysis comparing only users was not performed."

19) All important harms or unintended effects in each group

(for specific guidance see CONSORT for harms)

Does your paper address CONSORT subitem 19? *

Copy and paste relevant sections from the manuscript (include quotes in quotation marks "like this" to indicate direct quotes from your manuscript), or elaborate on this item by providing additional information not in the ms, or briefly explain why the item is not applicable/relevant for your study

No important harms or unintended effects were reported in any group. However, some participants experienced mild cybersickness, which was addressed in a separate study currently under review.

19-i) Include privacy breaches, technical problems

Include privacy breaches, technical problems. This does not only include physical “harm” to participants, but also incidents such as perceived or real privacy breaches [1], technical problems, and other unexpected/unintended incidents. “Unintended effects” also includes unintended positive effects [2].

	1	2	3	4	5	
subitem not at all important	◉	○	○	○	○	essential

ล้างสิ่งที่เลือก

Does your paper address subitem 19-i?

Copy and paste relevant sections from the manuscript (include quotes in quotation marks "like this" to indicate direct quotes from your manuscript), or elaborate on this item by providing additional information not in the ms, or briefly explain why the item is not applicable/relevant for your study

No privacy breaches or technical problems were reported.

19-ii) Include qualitative feedback from participants or observations from staff/researchers

Include qualitative feedback from participants or observations from staff/researchers, if available, on strengths and shortcomings of the application, especially if they point to unintended/unexpected effects or uses. This includes (if available) reasons for why people did or did not use the application as intended by the developers.

	1	2	3	4	5	
subitem not at all important	○	○	○	○	◉	essential

ล้างสิ่งที่เลือก

Does your paper address subitem 19-ii?

Copy and paste relevant sections from the manuscript (include quotes in quotation marks "like this" to indicate direct quotes from your manuscript), or elaborate on this item by providing additional information not in the ms, or briefly explain why the item is not applicable/relevant for your study

Yes, qualitative feedback from participants and observations from participants were collected and analyzed in a separate qualitative study, which is currently under peer review.

DISCUSSION

22) Interpretation consistent with results, balancing benefits and harms, and considering other relevant evidence

NPT: In addition, take into account the choice of the comparator, lack of or partial blinding, and unequal expertise of care providers or centers in each group

22-i) Restate study questions and summarize the answers suggested by the data, starting with primary outcomes and process outcomes (use)

Restate study questions and summarize the answers suggested by the data, starting with primary outcomes and process outcomes (use).

	1	2	3	4	5	
subitem not at all important	○	○	○	○	◉	essential

ล้างสิ่งที่เลือก

Does your paper address subitem 22-i? *

Copy and paste relevant sections from the manuscript (include quotes in quotation marks "like this" to indicate direct quotes from your manuscript), or elaborate on this item by providing additional information not in the ms, or briefly explain why the item is not applicable/relevant for your study

" These innovative IPE strategies are designed to help multiprofessional students manage stress, reduce burnout, and develop collaborative problem-solving skills within authentic simulation environments. By alleviating the pressures and risks typically associated with clinical practice, the interactive and scalable 3D computer-based ER-VIPE platform supports 21st-century healthcare learners, where patient safety is paramount. Integrating these tools and strategies into IPE programs offers significant potential to enhance well-being and resilience while preparing healthcare students and early-career professionals for a smooth transition into clinical practice."

22-ii) Highlight unanswered new questions, suggest future research

Highlight unanswered new questions, suggest future research.

	1	2	3	4	5	
subitem not at all important	○	○	○	○	◉	essential

ล้างสิ่งที่เลือก

Does your paper address subitem 22-ii?

Copy and paste relevant sections from the manuscript (include quotes in quotation marks "like this" to indicate direct quotes from your manuscript), or elaborate on this item by providing additional information not in the ms, or briefly explain why the item is not applicable/relevant for your study

". Future studies should consider including participants with mild or well-managed mental health comorbidities to enhance the generalizability and applicability of the intervention to more diverse, real-world clinical settings."

20) Trial limitations, addressing sources of potential bias, imprecision, and, if relevant, multiplicity of analyses

20-i) Typical limitations in ehealth trials

Typical limitations in ehealth trials: Participants in ehealth trials are rarely blinded. Ehealth trials often look at a multiplicity of outcomes, increasing risk for a Type I error. Discuss biases due to non-use of the intervention/usability issues, biases through informed consent procedures, unexpected events.

	1	2	3	4	5	
subitem not at all important	○	○	○	○	◉	essential

ล้างสิ่งที่เลือก

Does your paper address subitem 20-i? *

Copy and paste relevant sections from the manuscript (include quotes in quotation marks "like this" to indicate direct quotes from your manuscript), or elaborate on this item by providing additional information not in the ms, or briefly explain why the item is not applicable/relevant for your study

"This study was conducted during the mid-COVID-19 pandemic, limiting its comparability to conventional clinical teaching, which is primarily conducted in clinical settings."

21) Generalisability (external validity, applicability) of the trial findings

NPT: External validity of the trial findings according to the intervention, comparators, patients, and care providers or centers involved in the trial

21-i) Generalizability to other populations

Generalizability to other populations: In particular, discuss generalizability to a general Internet population, outside of a RCT setting, and general patient population, including applicability of the study results for other organizations

	1	2	3	4	5	
subitem not at all important	○	○	○	○	◉	essential

ล้างสิ่งที่เลือก

Does your paper address subitem 21-i?

Copy and paste relevant sections from the manuscript (include quotes in quotation marks "like this" to indicate direct quotes from your manuscript), or elaborate on this item by providing additional information not in the ms, or briefly explain why the item is not applicable/relevant for your study

"This study has several limitations. Conducted in a single university-based setting, the findings may have limited generalizability to other settings that differ in context and available facilities. "

21-ii) Discuss if there were elements in the RCT that would be different in a routine application setting

Discuss if there were elements in the RCT that would be different in a routine application setting (e.g., prompts/reminders, more human involvement, training sessions or other co-interventions) and what impact the omission of these elements could have on use, adoption, or outcomes if the intervention is applied outside of a RCT setting.

	1	2	3	4	5	
subitem not at all important	(●)	( )	( )	( )	( )	essential

ล้างสิ่งที่เลือก

Does your paper address subitem 21-ii?

Copy and paste relevant sections from the manuscript (include quotes in quotation marks "like this" to indicate direct quotes from your manuscript), or elaborate on this item by providing additional information not in the ms, or briefly explain why the item is not applicable/relevant for your study

No elements in this RCT were identified that would differ significantly in a routine application setting.

OTHER INFORMATION

23) Registration number and name of trial registry

Does your paper address CONSORT subitem 23? *

Copy and paste relevant sections from the manuscript (include quotes in quotation marks "like this" to indicate direct quotes from your manuscript), or elaborate on this item by providing additional information not in the ms, or briefly explain why the item is not applicable/relevant for your study

No trial registration number was obtained, as the study was not registered in a public trial registry.

24) Where the full trial protocol can be accessed, if available

Does your paper address CONSORT subitem 24? *

Cite a Multimedia Appendix, other reference, or copy and paste relevant sections from the manuscript (include quotes in quotation marks "like this" to indicate direct quotes from your manuscript), or elaborate on this item by providing additional information not in the ms, or briefly explain why the item is not applicable/relevant for your study

No, the full trial protocol is not publicly available.

25) Sources of funding and other support (such as supply of drugs), role of funders

Does your paper address CONSORT subitem 25? *

Copy and paste relevant sections from the manuscript (include quotes in quotation marks "like this" to indicate direct quotes from your manuscript), or elaborate on this item by providing additional information not in the ms, or briefly explain why the item is not applicable/relevant for your study

This research was funded by the Second Century Fund, Chulalongkorn University.

X27) Conflicts of Interest (not a CONSORT item)

X27-i) State the relation of the study team towards the system being evaluated

In addition to the usual declaration of interests (financial or otherwise), also state the relation of the study team towards the system being evaluated, i.e., state if the authors/evaluators are distinct from or identical with the developers/sponsors of the intervention.

	1	2	3	4	5	
subitem not at all important	○	○	○	○	◉	essential

ล้างสิ่งที่เลือก

Does your paper address subitem X27-i?

Copy and paste relevant sections from the manuscript (include quotes in quotation marks "like this" to indicate direct quotes from your manuscript), or elaborate on this item by providing additional information not in the ms, or briefly explain why the item is not applicable/relevant for your study

The authors declare no conflicts of interest.

About the CONSORT EHEALTH checklist

As a result of using this checklist, did you make changes in your manuscript? *

- ○ yes, major changes
- ◉ yes, minor changes
- ○ no

What were the most important changes you made as a result of using this checklist?

Participants and sampling
After IRB approval, undergraduate clinical students from five healthcare disciplines (medicine, nursing, pharmacy, radiologic technology, and medical technology) were recruited via announcements, Line, and posters. Interested students enrolled through a QR-linked Google Form. The principal investigator's contact was provided for inquiries. Participation was voluntary and scheduled outside of regular academic activities to avoid disruption.

How much time did you spend on going through the checklist INCLUDING making changes in your manuscript *

We spent approximately two hours reviewing the checklist and making the necessary revisions to the manuscript to ensure full alignment with the reporting standards.

As a result of using this checklist, do you think your manuscript has improved? *

- (selected) yes
- no
- อื่นๆ:

Would you like to become involved in the CONSORT EHEALTH group?

This would involve for example becoming involved in participating in a workshop and writing an "Explanation and Elaboration" document

- yes
- (selected) no
- อื่นๆ:

ล้างสิ่งที่เลือก

Any other comments or questions on CONSORT EHEALTH

คำตอบของคุณ

STOP - Save this form as PDF before you click submit

To generate a record that you filled in this form, we recommend to generate a PDF of this page (on a Mac, simply select "print" and then select "print as PDF") before you submit it.

When you submit your (revised) paper to JMIR, please upload the PDF as supplementary file.

Don't worry if some text in the textboxes is cut off, as we still have the complete information in our database. Thank you!

Final step: Click submit !

Click submit so we have your answers in our database!

ส่ง   ล้างแบบฟอร์ม

ห้ามส่งรหัสผ่านใน Google ฟอร์ม

แบบฟอร์มนี้ถูกสร้างขึ้นนอกโดเมนของคุณ - ติดต่อเจ้าของแบบฟอร์ม - ข้อกำหนดในการให้บริการ - นโยบายความเป็นส่วนตัว

แบบฟอร์มนี้ดูน่าสงสัยใช่ไหม รายงาน

Google ฟอร์ม
