# Supplementary material for: Comparing the Effectiveness of Multimodal Learning Using Computer-Based and Immersive Virtual Reality Simulation–Based Interprofessional Education With Co-Debriefing, Medical Movies, and Massive Online Open Courses for Mitigating Stress and Long-Term Burnout in Medical Training: Quasi-Experimental Study
Source: JMIR Med Educ. 2025 Sep 24;11:e70726. doi: 10.2196/70726 (PMC12508677; doi:10.2196/70726)
Supplement: Multimedia Appendix 4 [file mededu_v11i1e70726_app4.docx]

**Multimedia Appendix 1: Measurement Instruments for State Stress, Burnout, and Anxiety Trait**

**A measure of state stress:** The short-version Dundee Stress State Questionnaire (DSSQ; (1)) was used to assess the level of subjective stress state. Prof. Helton granted us permission for its use. The DSSQ is a 24-item multidimensional measurement comprising task engagement, distress, and worry. The items are presented in a 5-point Likert scale (1=not at all and 5=extremely).

Engagement are Items: 2, 5, 11, 12, 13, 17, 21, and 22

Distress are items: 1, 3, 4, 6, 7, 8, 9, and 10

Worry are items: 14, 15, 16, 18, 19, 20, 23, and 24


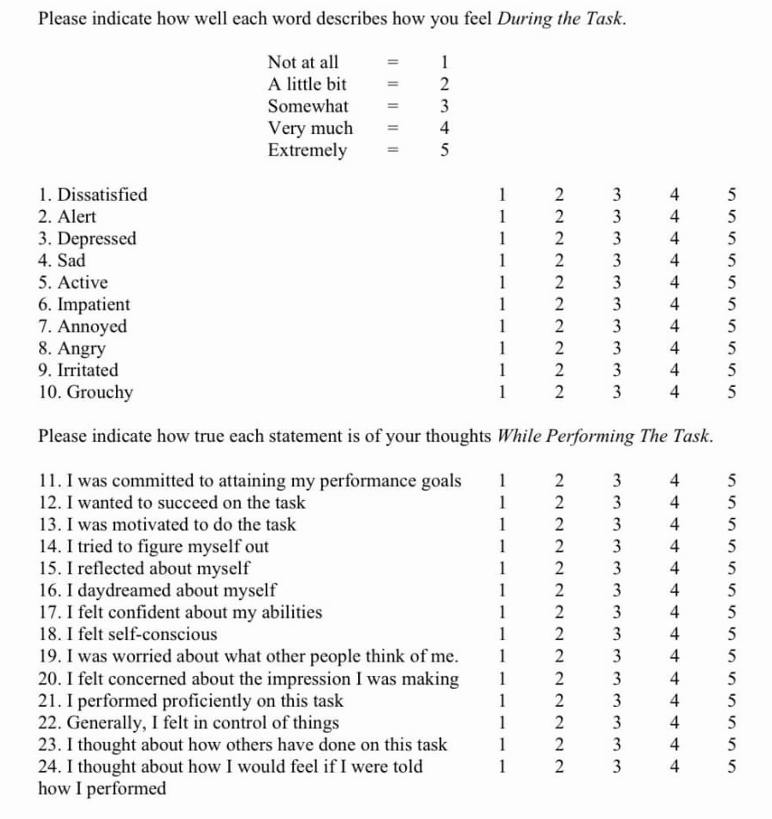


**A measure of burnout:** A 6-item personal burnout subscale from the Copenhagen Burnout Inventory (CBI; (2)) was used to assess the level of prolonged physical and psychological exhaustion. The items are presented in a 5-point Likert scale (1=never/almost never and 5=always). Please complete the following items. There are no right or wrong answers. In general,


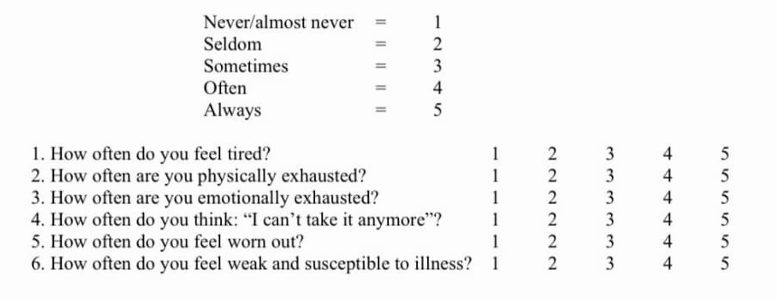


**A measure of anxiety trait:** The subscale of trait anxiety (STAI) from the short version of the Spielberger State-Trait Anxiety Inventory (STAI; (3)). has been ordered and paid for with the necessary permissions obtained. It is used to assess an individual propensity to anxiety. There are 5 items, and the items are presented in a 4-point Likert scale (1=not at all and 4=very much so).

**
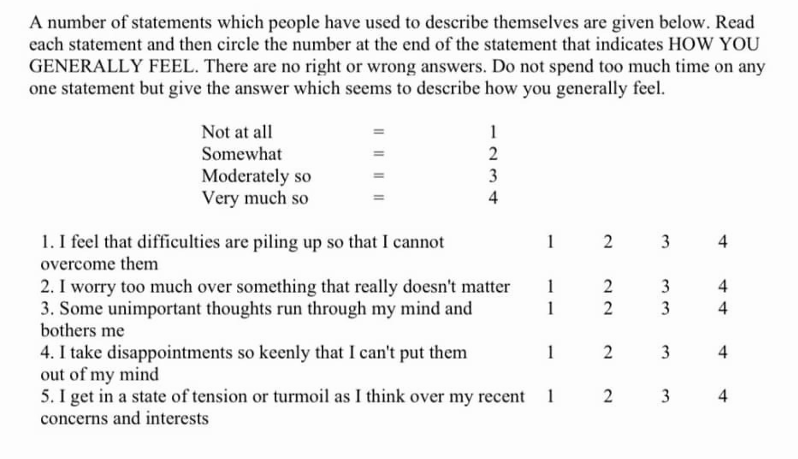
**
