# Supplementary material for: Comparing the Effectiveness of Multimodal Learning Using Computer-Based and Immersive Virtual Reality Simulation–Based Interprofessional Education With Co-Debriefing, Medical Movies, and Massive Online Open Courses for Mitigating Stress and Long-Term Burnout in Medical Training: Quasi-Experimental Study
Source: JMIR Med Educ. 2025 Sep 24;11:e70726. doi: 10.2196/70726 (PMC12508677; doi:10.2196/70726)
Supplement: Multimedia Appendix 5 [file mededu_v11i1e70726_app5.docx]

**Table S1. Baseline Burnout and DSSQ Scores (Engagement, Distress, Worry) Across Groups A, B, and C.**

| **Factor** | **Total (n=87)** | **Group A**^a^ **(n=29)** | **Group B**^b^ **(n=29)** | **Group C**^c^ **(n=29)** | *P* **value** |
| --- | --- | --- | --- | --- | --- |
| **Burnout** |  |  |  |  |  |
| Mean (SD) | 13.82 (4.11) | 14 (4.40) | 13.86 (3.62) | 13.59 (4.40) | - |
| Median (IQR) | 13 (11-16) | 14 (10-16) | 13 (11-16) | 12 (11-17) | .91^d^ |
| **DSSQ-Engagement** |  |  |  |  |  |
| Mean (SD) | 26.63 (5.04) | 25.55 (5.32) | 27.79 (3.69) | 26.55 (5.78) | - |
| Median (IQR) | 27 (23-30) | 26 (21-30) | 29 (26-30) | 27 (23-31) | .36^d^ |
| **DSSQ -Distress** |  |  |  |  |  |
| Mean (SD) | 10.11 (3.65) | 9.68 (2.89) | 10.41 (4.38) | 10.21 (3.59) | - |
| Median (IQR) | 9 (8-10) | 8.5 (8-10) | 9 (8-10) | 8.5 (8-10.5) | .73^d^ |
| **DSSQ -Worry** |  |  |  |  |  |
| Mean (SD) | 25.48 (6.70) | 26.93 (5.89) | 23.24 (5.92) | 26.31 (7.74) | - |
| Median (IQR) | 25 (20-30) | 27 (24-30) | 24 (19-26) | 28 (22-30) | .07^d^ |

**^a^ Group A** (control) participated in a 3D computer-based SIMBIE without oral debriefing; **^b^ Group B** received a medical movie, a MOOC, a 3D computer-based SIMBIE, and an oral co-debriefing session; **^c^ Group C** received a medical movie, a MOOC, a 3D virtual reality SIMBIE, and an oral co-debriefing session; **Statistical test:** ^d^ Kruskal-Wallis H test.
