# Supplementary material for: Comparing the Effectiveness of Multimodal Learning Using Computer-Based and Immersive Virtual Reality Simulation–Based Interprofessional Education With Co-Debriefing, Medical Movies, and Massive Online Open Courses for Mitigating Stress and Long-Term Burnout in Medical Training: Quasi-Experimental Study
Source: JMIR Med Educ. 2025 Sep 24;11:e70726. doi: 10.2196/70726 (PMC12508677; doi:10.2196/70726)
Supplement: Multimedia Appendix 6 [file mededu_v11i1e70726_app6.docx]

**Table S2: Time Interval Assessments Across Groups**

| **Factor** | **DSSQ Assessment Interval**  **(VR Sim-Movie MOOC, Days)** | | | | **Burnout Assessment Interval**  **(Follow up-VR Sim, Days)** | | | |  |
| --- | --- | --- | --- | --- | --- | --- | --- | --- | --- |
|  | **N** | **Mean (SD)** | **Median (IQR)** | **Min-Max** | **N** | **Mean (SD)** | **Median (IQR)** | **Min-Max** |  |
| **All** | 86 | 8.78 (12.44) | 6.50 (3-10) | 1-96 | 62 | 38.66 (15.24) | 34 (33-36) | 23-103 |  |
| **Group** |  |  |  |  |  |  |  |  |  |
| Group A^a^ | 29 | 11.52 (20.43) | 5 (3-9) | 1-96 | 22 | 33.82 (12.90) | 32 (29-35) | 23-87 |  |
| Group B^b^ | 28 | 7.61 (4.46) | 7.50 (3-10) | 1-17 | 15 | 48.20 (23.48) | 34 (33-58) | 33-103 |  |
| Group C^c^ | 29 | 7.17 (4.65) | 8 (3-10) | 1-19 | 25 | 37.20 (6.52) | 35 (34-36) | 32-62 |  |
| *P* value |  |  |  |  |  |  |  |  |  |
| **All** | - | - | 0.63^d^ | - | - | - | .002^d^ | - |  |
| A vs B | - | - | - | - | - | - | .005^e^ | - |  |
| A vs C | - | - | - | - | - | - | .003^e^ | - |  |
| B vs C | - | - | - | - | - | - | >.99^e^ | - |  |
| **Specialty** |  |  |  |  |  |  |  |  |  |
| Medicine | 15 | 8.13 (4.47) | 9 (4-10) | 1-16 | 8 | 35.63 (3.96) | 34.50 (33.50-37) | 31-44 |  |
| Pharmacy | 15 | 16.33 (27.76) | 4 (3-13) | 1-96 | 11 | 40.91 (19.33) | 34 (33-36) | 24-92 |  |
| Radiologist | 12 | 6.75 (2.67) | 7.50 (4-9) | 3-10 | 9 | 39.22 (8.97) | 37 (34-44) | 29-58 |  |
| Med Tech | 15 | 9.20 (4.89) | 9 (5-13) | 2-19 | 11 | 39.45 (17.40) | 35 (30-47) | 24-87 |  |
| Nurse-Air | 15 | 6.20 (4.16) | 5 (3-9) | 1-17 | 13 | 36.77(12.57) | 33 (32-36) | 23-76 |  |
| Nurse-Cir | 14 | 5.43 (4.50) | 3 (2-9) | 1-15 | 10 | 39.70 (22.51) | 33.50 (32-36) | 24-103 |  |

The time interval (in days) between the post-intervention DSSQ assessment in Phase 1 and the pre-intervention DSSQ assessment in Phase 2 was compared across Groups A, B, and C. Additionally, the time interval for burnout duration between the post-intervention assessment in Phase 2 and the final assessment in Phase 3 was evaluated. ^a^ **Group A** (control) participated in a 3D computer-based SIMBIE without oral debriefing; ^b^ **Group B** received a medical movie, a MOOC, a 3D computer-based SIMBIE, and an oral co-debriefing session; ^c^ **Group C** received a medical movie, a MOOC, a 3D virtual reality SIMBIE, and an oral co-debriefing session; **Statistical tests**: ^d^ Kruskal-Wallis H test, ^e^ Dunn’s test. **Abbreviations**: MOOC, Massive Open Online Course; SIMBIE, Simulation-Based Interprofessional Education: CBI, Copenhagen Burnout Inventory, DSSQ, Dundee Stress State Questionnaire.
