# Supplementary material for: Comparing the Effectiveness of Multimodal Learning Using Computer-Based and Immersive Virtual Reality Simulation–Based Interprofessional Education With Co-Debriefing, Medical Movies, and Massive Online Open Courses for Mitigating Stress and Long-Term Burnout in Medical Training: Quasi-Experimental Study
Source: JMIR Med Educ. 2025 Sep 24;11:e70726. doi: 10.2196/70726 (PMC12508677; doi:10.2196/70726)
Supplement: Multimedia Appendix 7 [file mededu_v11i1e70726_app7.doc]

**Table S3. Improvement in Burnout and DSSQ Scores: Intention-to-Treat Analysis**

| **Factor** | **Group** | | | | | **Pairwise comparisons** | | |
| --- | --- | --- | --- | --- | --- | --- | --- | --- |
|  | **Group A** | | **Group B** | | **Group C** | **B vs A** | **C vs A** | **C vs B** |
|  | **(n=29)** | | **(n=29)** | | **(n=29)** |  |  |  |
| **Burnout outcome** | |  | |  |  |  |  |  |
| Pre-Intervention  Assessment Phase 1 | | 13.74 (12.65, 14.83) | | 13.67 (12.58, 14.76) | 13.46 (12.38, 14.55) |  |  |  |
| Pre-Intervention  Assessment Phase 2 | | 13.29 (12.20, 14.38) | | 12.30 (11.20, 13.40) | 12.28 (11.19, 13.38) |  |  |  |
| Final  Assessment Phase 3 | | 14.12 (13.03, 15.21) | | 12.19 (11.10, 13.28) | 13.57 (12.48, 14.65) |  |  |  |
| *P* value | | .29 | | .01 | .03 |  |  |  |
| **Pairwise comparisons** | |  | |  |  |  |  |  |
| Phase 2 vs Phase 1 | | −0.42 (−1.48, 0.64) | | −1.35 (−2.41, −0.29) | −1.16 (−2.22, −0.10) | −0.93 (−2.41, 0.56) | −0.74 (−2.21, 0.74) | 0.19 (−1.30, 1.68) |
| *P* value | | .43 | | .01 | .03 | 0.22 | 0.33 | 0.80 |
| Phase 3 vs Phase 1 | | 0.42 (−0.62, 1.47) | | −1.44 (−2.48, −0.39) | 0.15 (−0.90, 1.19) | −1.86 (−3.34, −0.39) | −0.28 (−1.75, 1.20) | 1.59 (0.11, 3.06) |
| *P* value | | .43 | | .007 | .78 | .01 | .71 | .04 |
| Phase 3 vs Phase 2 | | 0.85 (−0.21, 1.90) | | −0.09 (−1.15, 0.97) | 1.31 (0.25, 2.36) | −0.94 (−2.43, 0.55) | 0.46 (−1.02, 1.94) | 1.40 (−0.09, 2.89) |
| *P* value | | .12 | | .88 | .02 | .22 | .54 | .07 |
| **DSSQ-Engagement** | |  | |  |  |  |  |  |
| Phase 1 | |  | |  |  |  |  |  |
| Pre | | 25.56 (23.72, 27.39) | | 27.79 (25.96, 29.63) | 26.55 (24.71, 28.38) |  |  |  |
| Post | | 23.42 (21.58, 25.26) | | 29.59 (27.75, 31.42) | 26.48 (24.64, 28.31) |  |  |  |
| Post vs Pre | | −2.21 (−3.83, −0.58) | | 1.72 (0.10, 3.35) | −0.14 (−1.76, 1.48) | 3.93 (1.64, 6.22) | 2.07 (−0.22, 4.36) | −1.86 (−4.16, 0.43) |
| *P* value | | .008 | | .04 | .87 | .001 | .08 | .11 |
| Phase 2 | |  | |  |  |  |  |  |
| Pre | | 23.75 (21.89, 25.60) | | 26.86 (25.01, 28.72) | 26.15 (24.29, 28.00) |  |  |  |
| Post | | 26.64 (24.79, 28.50) | | 29.45 (27.59, 31.31) | 27.87 (26.02, 29.73) |  |  |  |
| Post vs Pre | | 2.92 (1.45, 4.40) | | 2.61 (1.14, 4.09) | 1.75 (0.27, 3.23) | −0.31 (−2.40, 1.78) | −1.17 (−3.26, 0.92) | −0.86 (−2.95, 1.23) |
| *P* value | | <.001 | | .001 | .02 | .77 | .27 | .42 |
| **DSSQ-Distress** | |  | |  |  |  |  |  |
| Phase 1 | |  | |  |  |  |  |  |
| Pre | | 9.74 (8.83, 10.65) | | 10.40 (9.51, 11.30) | 10.20 (9.29, 11.11) |  |  |  |
| Post | | 8.55 (7.66, 9.45) | | 9.91 (9.02, 10.81) | 9.83 (8.93, 10.72) |  |  |  |
| Post vs Pre | | −1.22 (−2.46, 0.02) | | −0.52 (−1.74, 0.71) | −0.40 (−1.64, 0.84) | 0.70 (−1.04, 2.44) | 0.82 (−0.93, 2.57) | 0.12 (−1.62, 1.86) |
| *P* value | | .05 | | .41 | .53 | .43 | .36 | .90 |
| Phase 2 | |  | |  |  |  |  |  |
| Pre | | 9.01 (8.14, 9.88) | | 9.86 (8.98, 10.73) | 10.06 (9.19, 10.94) |  |  |  |
| Post | | 9.60 (8.73, 10.47) | | 9.75 (8.88, 10.63) | 10.27 (9.40, 11.14) |  |  |  |
| Post vs Pre | | 0.55 (−0.21, 1.30) | | −0.14 (−0.90, 0.61) | 0.17 (−0.59, 0.92) | −0.69 (−1.76, 0.38) | −0.38 (−1.45, 0.69) | 0.31 (−0.76, 1.38) |
| *P* value | | .16 | | .71 | .67 | .21 | .49 | .57 |
| **DSSQ-Worry** | |  | |  |  |  |  |  |
| Phase 1 | |  | |  |  |  |  |  |
| Pre | | 26.95 (24.81, 29.10) | | 23.23 (21.10, 25.36) | 26.24 (24.11, 28.37) |  |  |  |
| Post | | 28.90 (26.77, 31.03) | | 25.05 (22.93, 27.18) | 27.90 (25.77, 30.03) |  |  |  |
| Post vs Pre | | 2.04 (0.61, 3.47) | | 1.92 (0.51, 3.33) | 1.74 (0.34, 3.15) | −0.13 (−2.13, 1.88) | −0.30 (−2.30, 1.71) | −0.17 (−2.16, 1.82) |
| *P* value | | .005 | | .008 | .02 | .90 | .77 | .87 |
| Phase 2 | |  | |  |  |  |  |  |
| Pre | | 29.14 (27.14, 31.15) | | 24.88 (22.87, 26.89) | 26.36 (24.35, 28.36) |  |  |  |
| Post | | 28.04 (26.04, 30.04) | | 24.47 (22.46, 26.48) | 26.22 (24.21, 28.22) |  |  |  |
| Post vs Pre | | −1.07 (−2.34, 0.21) | | −0.38 (−1.65, 0.90) | −0.10 (−1.38, 1.18) | 0.69 (−1.11, 2.50) | 0.97 (−0.84, 2.77) | 0.28 (−1.53, 2.08) |
| *P* value | | .10 | | .56 | .88 | .45 | .30 | .77 |

**Group A** (control) with 3D computer-based SIMBIE without oral debriefing, **Group B** with a medical movie, MOOC, 3D computer-based SIMBIE, and oral co-debriefing, and **Group C** with a medical movie, MOOC, 3D VR SIMBIE, and oral co-debriefing. **Burnout** is assessed across three phases: **Phase 1** (pre-Movie and MOOC, before EEG cap fitting), **Phase 2** (pre-SIMBIE intervention), and **Phase 3** (six-week follow-up). **DSSQ** measures engagement, distress, and worry pre- and post-intervention. **Statistical analysis** using Generalized Estimating Equations (GEE), adjusted for anxiety traits as a control variable, reveals intervention effects based on an intention-to-treat analysis. **Abbreviations:** GEE: Generalized Estimating Equations, DSSQ: Dundee Stress State Questionnaire.
