# Supplementary material for: Comparing the Effectiveness of Multimodal Learning Using Computer-Based and Immersive Virtual Reality Simulation–Based Interprofessional Education With Co-Debriefing, Medical Movies, and Massive Online Open Courses for Mitigating Stress and Long-Term Burnout in Medical Training: Quasi-Experimental Study
Source: JMIR Med Educ. 2025 Sep 24;11:e70726. doi: 10.2196/70726 (PMC12508677; doi:10.2196/70726)
Supplement: Multimedia Appendix 8 [file mededu_v11i1e70726_app8.docx]

**Table S4. Improvement of Burnout and DSSQ Scores (Per-protocol Analysis)**

| **Factor** | **Group** | | | **Pairwise comparisons** | | |
| --- | --- | --- | --- | --- | --- | --- |
|  | **Group A (n=29)** | **Group B  (n=29)** | **Group C  (n=29)** | **B vs A** | **C vs A** | **C vs B** |
| **Burnout outcome** |  |  |  |  |  |  |
| Pre-Intervention Assessment Phase 1 | 13.59 (12.54, 14.65) | 13.53 (12.48, 14.58) | 13.34 (12.29, 14.38) |  |  |  |
| Pre-Intervention Assessment Phase 2 | 13.22 (12.17, 14.27) | 12.19 (11.13, 13.25) | 12.23 (11.17, 13.28) |  |  |  |
| Final Assessment Phase 3 | 14.53 (13.36, 15.70) | 12.45 (11.13, 13.77) | 13.85 (12.75, 14.96) |  |  |  |
| *P* value | .08 | .045 | .01 |  |  |  |
| **Pairwise comparisons** |  |  |  |  |  |  |
| Phase 2 vs Phase 1 | −0.34 (−1.40, 0.72) | −1.31 (−2.37, −0.24) | −1.07 (−2.13, −0.01) | −0.97 (−2.46, 0.52) | −0.73 (−2.21, 0.75) | 0.23 (−1.26, 1.72) |
| *P* value | .53 | .02 | .048 | .20 | .33 | .76 |
| Phase 3 vs Phase 1 | 1.00 (−0.17, 2.17) | −1.02 (−2.34, 0.30) | 0.58 (−0.52, 1.69) | −2.02 (−3.77, −0.27) | −0.42 (−2.03, 1.19) | 1.60 (−0.13, 3.33) |
| *P* value | .09 | .13 | .30 | .02 | .61 | .07 |
| Phase 3 vs Phase 2 | 1.34 (0.16, 2.52) | 0.29 (−1.05, 1.62) | 1.65 (0.54, 2.77) | −1.06 (−2.82, 0.71) | 0.31 (−1.30, 1.92) | 1.37 (−0.37, 3.11) |
| *P* value | .03 | .68 | .004 | .24 | .70 | .12 |
| **DSSQ-Engagement** |  |  |  |  |  |  |
| Phase 1 |  |  |  |  |  |  |
| Pre | 25.56 (23.72, 27.39) | 27.79 (25.96, 29.63) | 26.55 (24.71, 28.38) |  |  |  |
| Post | 23.42 (21.58, 25.26) | 29.59 (27.75, 31.42) | 26.48 (24.64, 28.31) |  |  |  |
| Post vs Pre | −2.21 (−3.83,−0.58) | 1.72 (0.10, 3.35) | −0.14 (−1.76, 1.48) | 3.93 (1.64, 6.22) | 2.07 (−0.22, 4.36) | −1.86 (−4.16,0.43) |
| *P* value | .008 | .04 | .87 | .001 | .08 | .11 |
| Phase 2 |  |  |  |  |  |  |
| Pre | 23.77 (21.97, 25.57) | 26.65 (24.81, 28.48) | 25.91 (24.09, 27.73) |  |  |  |
| Post | 26.95 (25.13, 28.77) | 29.14 (27.28, 30.99) | 28.01 (26.19, 29.84) |  |  |  |
| Post vs Pre | 3.20 (1.69, 4.71) | 2.51 (0.97, 4.04) | 2.12 (0.59, 3.65) | −0.69 (−2.84, 1.46) | −1.08 (−3.23, 1.07) | −0.39 (−2.55, 1.78) |
| *P* value | <.001 | .001 | .007 | .53 | .33 | .73 |
| **DSSQ-Distress** |  |  |  |  |  |  |
| Phase 1 |  |  |  |  |  |  |
| Pre | 9.72 (8.81, 10.63) | 10.38 (9.49, 11.28) | 10.18 (9.27, 11.09) |  |  |  |
| Post | 8.54 (7.65, 9.43) | 9.90 (9.01, 10.79) | 9.68 (8.77, 10.59) |  |  |  |
| Post vs Pre | −1.21 (−2.46, 0.03) | −0.51 (−1.75, 0.72) | −0.53 (−1.79, 0.73) | 0.70 (−1.05, 2.45) | 0.68 (−1.09, 2.45) | −0.02 (−1.78, 1.75) |
| *P* value | .06 | .41 | .41 | .43 | .45 | .99 |
| Phase 2 |  |  |  |  |  |  |
| Pre | 9.01 (8.13, 9.89) | 9.92 (9.01, 10.83) | 10.07 (9.19, 10.95) |  |  |  |
| Post | 9.60 (8.70, 10.50) | 9.84 (8.95, 10.74) | 10.28 (9.40, 11.16) |  |  |  |
| Post vs Pre | 0.54 (−0.25, 1.32) | −0.13 (−0.91, 0.66) | 0.16 (−0.60, 0.93) | −0.66 (−1.77, 0.45) | −0.37 (−1.47, 0.72) | 0.29 (−0.81, 1.38) |
| *P* value | .18 | .76 | .67 | .24 | .50 | .61 |
| **DSSQ-Worry** |  |  |  |  |  |  |
| Phase 1 |  |  |  |  |  |  |
| Pre | 26.95 (24.81, 29.10) | 23.23 (21.10, 25.36) | 26.24 (24.11, 28.37) |  |  |  |
| Post | 28.90 (26.77, 31.03) | 25.05 (22.93, 27.18) | 27.90 (25.77, 30.03) |  |  |  |
| Post vs Pre | 2.04 (0.61, 3.47) | 1.92 (0.51, 3.33) | 1.74 (0.34, 3.15) | −0.13 (−2.13, 1.88) | −0.30 (−2.30, 1.71) | −0.17 (−2.16, 1.82) |
| *P* value | .005 | .008 | .02 | .90 | .77 | .87 |
| Phase 2 |  |  |  |  |  |  |
| Pre | 29.14 (27.20, 31.08) | 25.29 (23.31, 27.28) | 26.36 (24.41, 28.30) |  |  |  |
| Post | 27.93 (25.97, 29.89) | 24.92 (22.94, 26.90) | 26.18 (24.23, 28.13) |  |  |  |
| Post vs Pre | −1.14 (−2.48, 0.20) | −0.30 (−1.64, 1.03) | −0.11 (−1.42, 1.21) | 0.84 (−1.05, 2.72) | 1.04 (−0.83, 2.90) | 0.20 (−1.67, 2.07) |
| *P* value | .10 | .66 | .87 | .39 | .28 | .84 |

**Group A** (control) with 3D computer-based SIMBIE without oral debriefing, **Group B** with a medical movie, MOOC, 3D computer-based SIMBIE, and oral co-debriefing, and **Group C** with a medical movie, MOOC, 3D VR SIMBIE, and oral co-debriefing. Burnout is assessed across three phases: **Phase 1** (pre-Movie and MOOC, before EEG cap fitting), **Phase 2** (pre-SIMBIE intervention), and **Phase 3** (six-week follow-up). DSSQ measures engagement, distress, and worry pre- and post-intervention. **Statistical analysis** using Generalized Estimating Equations (GEE), adjusted for anxiety traits as a control variable, reveals intervention effects based on a per-protocol analysis. **Abbreviations:** GEE: Generalized Estimating Equations, DSSQ: Dundee Stress State Questionnaire.
