# Supplementary material for: Comparing the Effectiveness of Multimodal Learning Using Computer-Based and Immersive Virtual Reality Simulation–Based Interprofessional Education With Co-Debriefing, Medical Movies, and Massive Online Open Courses for Mitigating Stress and Long-Term Burnout in Medical Training: Quasi-Experimental Study
Source: JMIR Med Educ. 2025 Sep 24;11:e70726. doi: 10.2196/70726 (PMC12508677; doi:10.2196/70726)
Supplement: Multimedia Appendix 9 [file mededu_v11i1e70726_app9.docx]

**
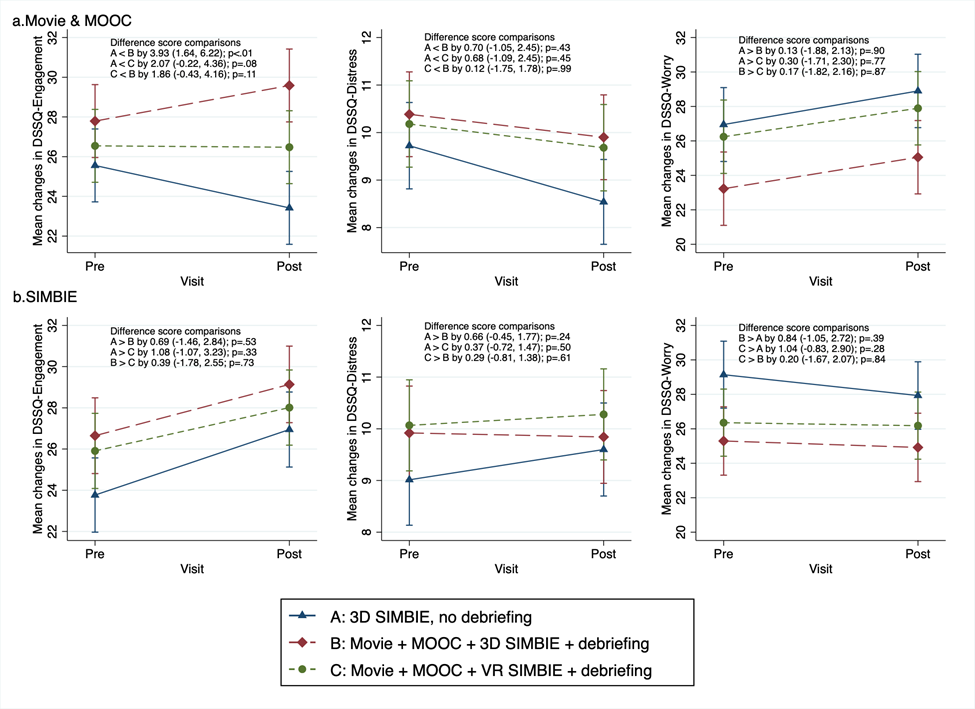
**

**Figure S1.** Illustrates changes in DSSQ scores based on per-protocol analysis. **The Y-axis** represents the mean change in DSSQ scores, while the **X-axis** shows pre- and post-intervention comparisons. **Group A** (control) used 3D computer-based SIMBIE without oral debriefing; **Group B** used a medical movie, MOOC, 3D computer-based SIMBIE, and oral co-debriefing; and **Group C** used a medical movie, MOOC, 3D VR SIMBIE, and oral co-debriefing. **Panel A** depicts the pre- and post-intervention effects of medical movies and MOOCs on DSSQ-Engagement (left), DSSQ-Distress (middle), and DSSQ-Worry (right). **Panel B** illustrates the effects of the SIMBIE process on the same DSSQ components. **Statistical analysis**, conducted using Generalized Estimating Equations (GEE) and adjusted for anxiety traits as a control variable, reveals intervention effects based on a per-protocol analysis. **Abbreviations:** MOOC, Massive Open Online Course; SIMBIE, Simulation-Based Interprofessional Education.
