# Supplementary material for: Comparing the Effectiveness of Multimodal Learning Using Computer-Based and Immersive Virtual Reality Simulation–Based Interprofessional Education With Co-Debriefing, Medical Movies, and Massive Online Open Courses for Mitigating Stress and Long-Term Burnout in Medical Training: Quasi-Experimental Study
Source: JMIR Med Educ. 2025 Sep 24;11:e70726. doi: 10.2196/70726 (PMC12508677; doi:10.2196/70726)
Supplement: Multimedia Appendix 10 [file mededu_v11i1e70726_app10.docx]

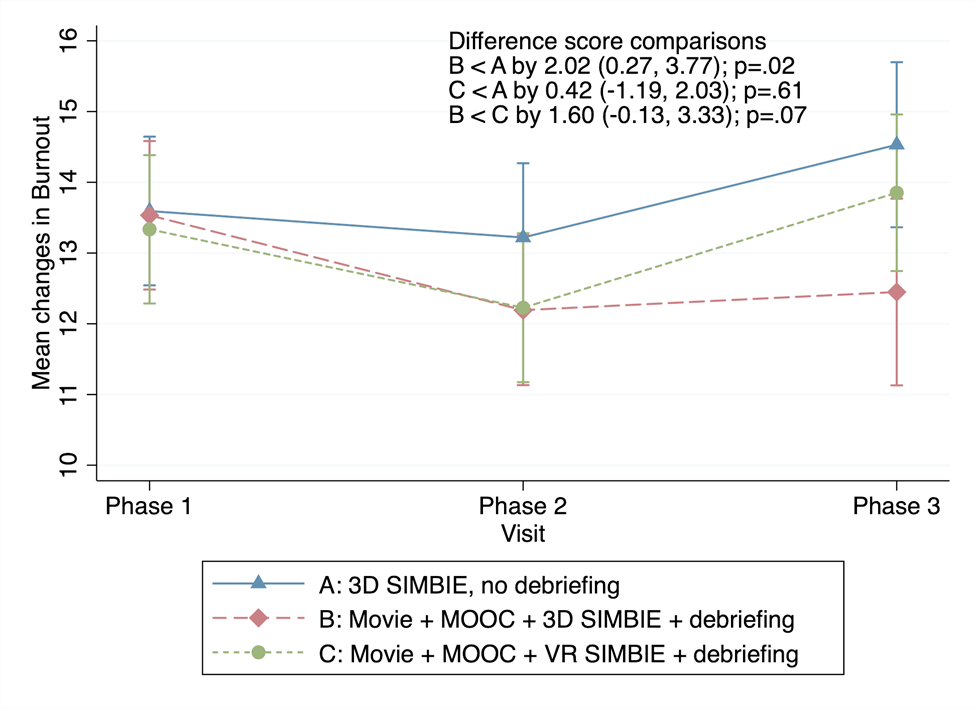


**Figure S2** Changes in Copenhagen Burnout Inventory (CBI) scores based on per-protocol analysis. **The Y-axis** represents the mean change in burnout levels, while the **X-axis** denotes three distinct phases: Phase 1 (pre-Movie and MOOC, before EEG cap fitting), Phase 2 (pre-SIMBIE intervention), and Phase 3 (six-week follow-up). **Group A** (control) used 3D computer-based SIMBIE without oral debriefing; **Group B** used a medical movie, MOOC, 3D computer-based SIMBIE, and oral co-debriefing; and **Group C** used a medical movie, MOOC, 3D VR SIMBIE, and oral co-debriefing. **Statistical analysis** was conducted using Generalized Estimating Equations (GEE), adjusted for anxiety traits as a control variable, reveals intervention effects based on a **per-protocol analysis**.
